# Supplementary material for: Second-line TKI after first-line immunotherapy-based treatment in advanced HCC: Reconstructed IPD meta-analysis
Source: JHEP Rep. 2026 May 12;8(8):101893. doi: 10.1016/j.jhepr.2026.101893 (PMC13352052; doi:10.1016/j.jhepr.2026.101893)
Supplement: Multimedia component 1 [file mmc1.pdf]

Second-line TKI after first-line immunotherapy-based treatment in advanced HCC: Reconstructed IPD meta-analysis

Erman Akkus, Christian Hobeika, Julien Edeline, Clémence Hollande, Manon Allaire, Giuliana Amaddeo, Hélène Regnault, Marie Lequoy, Jean Charles Nault, Mohamed Bouattour

Table of contents

Supplementary figures……………………………………………………………...2

Supplementary tables…………………………………………………………..19

Supplementary figures

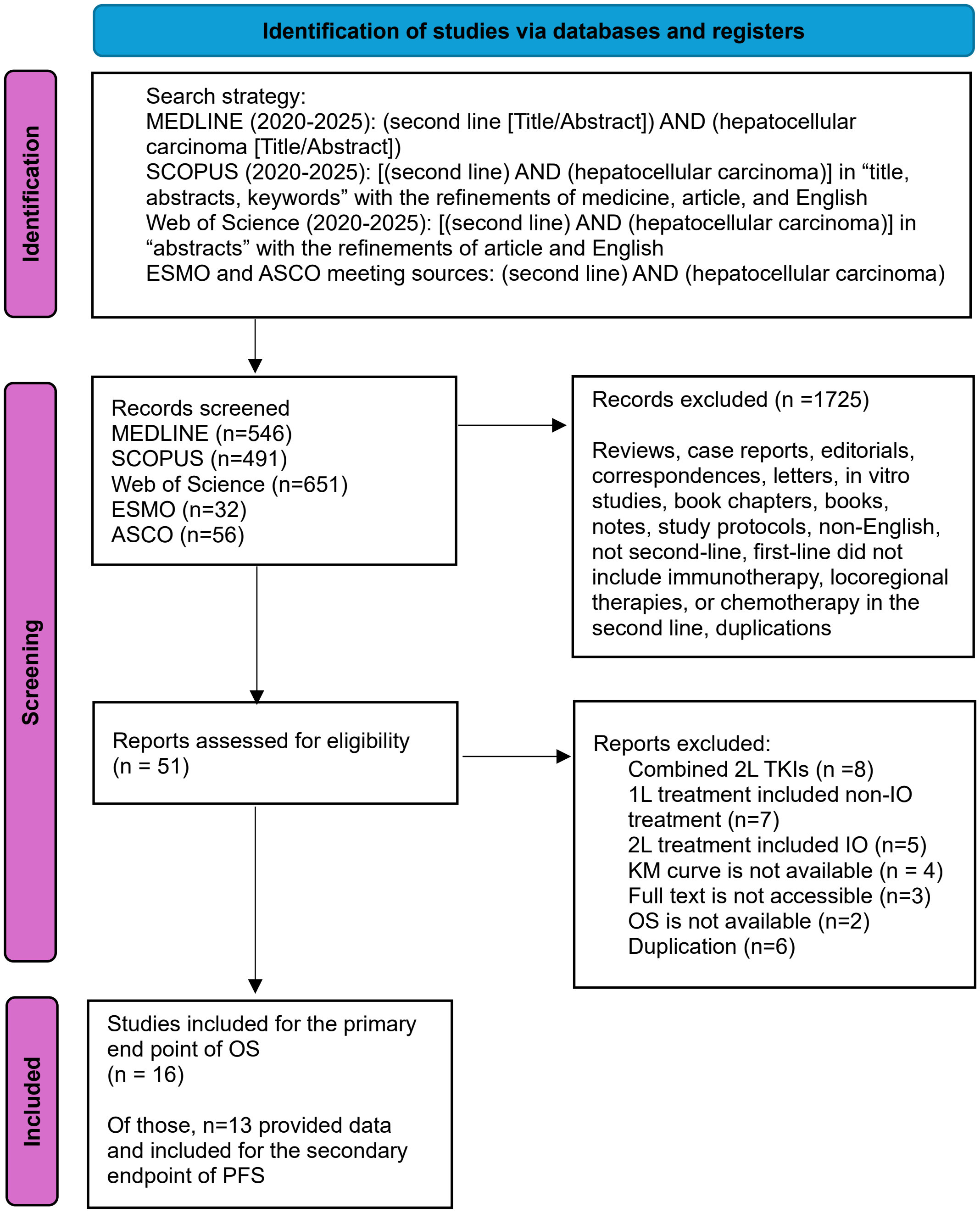

Fig. S1. PRISMA diagram of the study selection. ESMO: European Society of Medical Oncology, ASCO: American Society of Clinical Oncology, OS: Overall survival, PFS: Progression-free survival, TKI: tyrosine-kinase inhibitor, 1L: first-line, 2L: second-line, IO: immunotherapy, KM: Kaplan-Meier

Original OS graph:

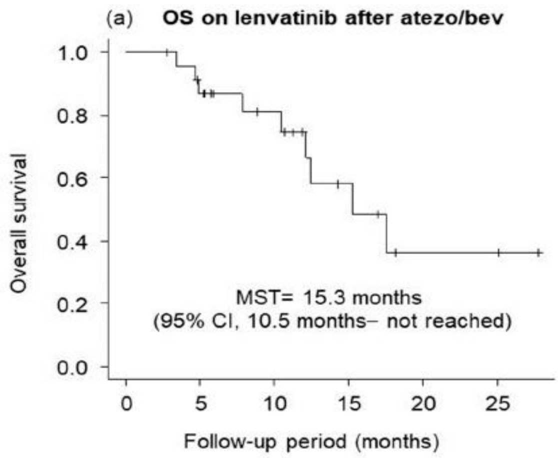

Reconstructed OS graph:

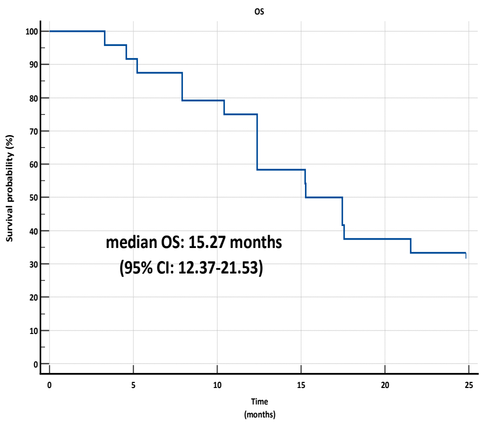

Fig. S2. Sample representation of reconstructed IPD data from published KM curves. Study: Yano S. et al, 2023, Lenvatinib

a) Percentage of patients with ***ECOG PS-0.*** *No difference.*

Study or Subgroup	Events	Total	Weight	IV, Random, 95% CI
**Subgroup = Lenva**				
Chon et al. 2023	26	40	5.9%	0.65 [0.48; 0.79]
Hiraoka et al. 2023	76	101	6.5%	0.75 [0.66; 0.83]
Lee et al. 2025	77	154	6.6%	0.50 [0.42; 0.58]
Lombardi et al. 2025	69	125	6.6%	0.55 [0.46; 0.64]
Muto et al. 2023	12	20	5.2%	0.60 [0.36; 0.81]
Piusieux et al. 2025	0	8	3.8%	0.00 [0.00; 0.37]
Yano S. et al. 2023	19	24	5.4%	0.79 [0.58; 0.93]
Yoo et al. 2024	9	50	6.1%	0.18 [0.09; 0.31]
Yoo et al. 2021	6	19	5.1%	0.32 [0.13; 0.57]
**Total (95% CI)**		**541**	**51.3%**	**0.49 [0.35; 0.64]**
Heterogeneity: $Tau^2 = 0.0389$; $Chi^2 = 77.11$, df = 8 (P < 0.0001); $I^2 = 89.6\%$				
**Subgroup = Sora**				
Chon et al. 2023	43	86	6.4%	0.50 [0.39; 0.61]
Lee et al. 2025	124	324	6.8%	0.38 [0.33; 0.44]
Lombardi et al. 2025	33	105	6.5%	0.31 [0.23; 0.41]
Piusieux et al. 2025	5	41	5.9%	0.12 [0.04; 0.26]
Yoo et al. 2021	5	29	5.6%	0.17 [0.06; 0.36]
**Total (95% CI)**		**585**	**31.3%**	**0.30 [0.20; 0.42]**
Heterogeneity: $Tau^2 = 0.0146$; $Chi^2 = 25.8$, df = 4 (P < 0.0001); $I^2 = 84.5\%$				
**Subgroup = Regora**				
Cheon et al. 2025	24	40	5.9%	0.60 [0.43; 0.75]
Lee et al. 2025	15	36	5.8%	0.42 [0.26; 0.59]
Piusieux et al. 2025	1	29	5.6%	0.03 [0.00; 0.18]
**Total (95% CI)**		**105**	**17.4%**	**0.32 [0.04; 0.70]**
Heterogeneity: $Tau^2 = 0.1022$; $Chi^2 = 30.79$, df = 2 (P < 0.0001); $I^2 = 93.5\%$				
**Total (95% CI)**		**1231**	**100.0%**	**0.40 [0.30; 0.50]**
**Prediction interval**				**[0.06; 0.81]**
Heterogeneity: $Tau^2 = 0.0368$; $Chi^2 = 176.39$, df = 16 (P < 0.0001); $I^2 = 90.9\%$				
Test for subgroup differences: $Chi^2 = 4.16$, df = 2 (P = 0.1250)				

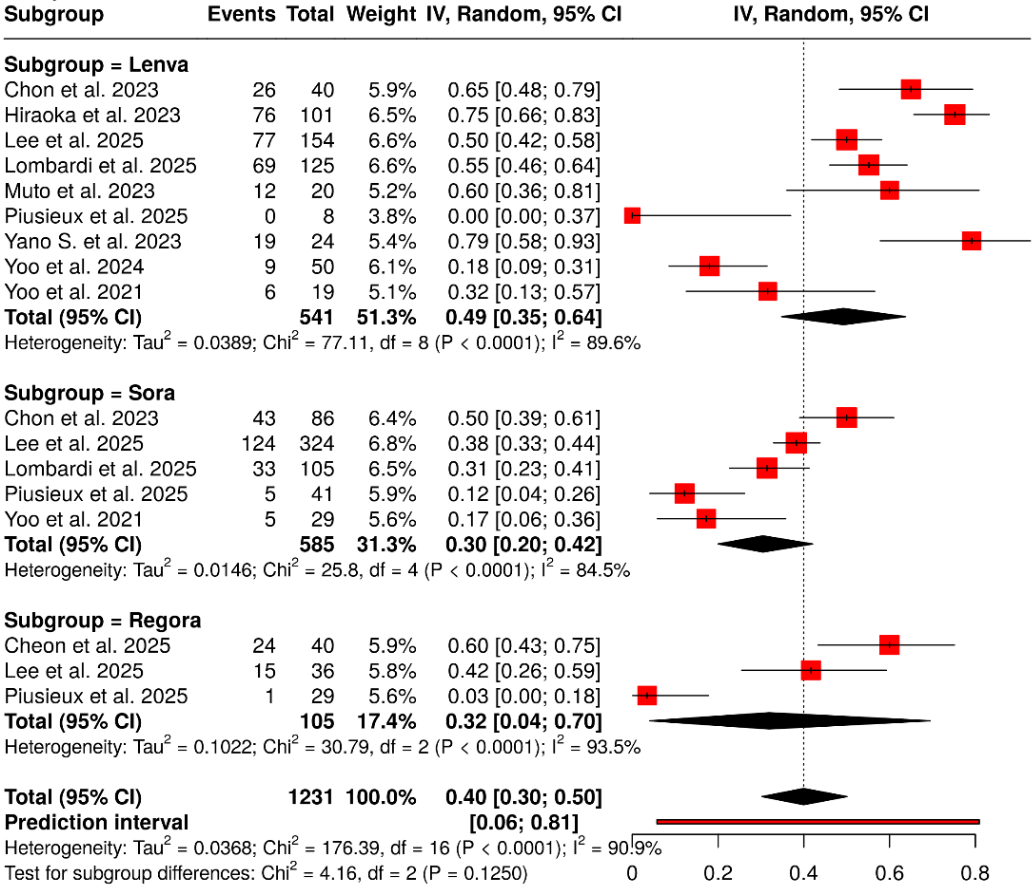

b) Percentage of patients with *Child-Pugh Class A.* *The regorafenib group had a significantly higher rate of CP-A*.

Study or Subgroup	Events	Total	Weight	IV, Random, 95% CI
**Subgroup = Lenva**				
Chen et al. 2022	6	9	3.5%	0.67 [0.30; 0.93]
Chon et al. 2023	37	40	5.2%	0.93 [0.80; 0.98]
Hiraoka et al. 2023	82	101	5.7%	0.81 [0.72; 0.88]
Lee et al. 2025	135	154	5.8%	0.88 [0.81; 0.92]
Muto et al. 2023	16	20	4.6%	0.80 [0.56; 0.94]
Persano et al. 2024	85	86	5.7%	0.99 [0.94; 1.00]
Piusieux et al. 2025	3	8	3.4%	0.38 [0.09; 0.76]
Qin et al. 2022	12	20	4.6%	0.60 [0.36; 0.81]
Yano S. et al. 2023	16	24	4.8%	0.67 [0.45; 0.84]
Yoo et al. 2024	50	50	5.4%	1.00 [0.93; 1.00]
Yoo et al. 2021	19	19	4.5%	1.00 [0.82; 1.00]
**Total (95% CI)**		**531**	**53.0%**	**0.86 [0.76; 0.94]**
Heterogeneity: $Tau^2 = 0.0352$; $Chi^2 = 73.06$, df = 10 ($P < 0.0001$); $I^2 = 86.3\%$				
**Subgroup = Sora**				
Chen et al. 2022	15	19	4.5%	0.79 [0.54; 0.94]
Chon et al. 2023	54	86	5.7%	0.63 [0.52; 0.73]
Lee et al. 2025	235	324	6.0%	0.73 [0.67; 0.77]
Persano et al. 2024	48	51	5.4%	0.94 [0.84; 0.99]
Piusieux et al. 2025	34	41	5.2%	0.83 [0.68; 0.93]
Yoo et al. 2021	29	29	4.9%	1.00 [0.88; 1.00]
**Total (95% CI)**		**550**	**31.7%**	**0.84 [0.71; 0.94]**
Heterogeneity: $Tau^2 = 0.0288$; $Chi^2 = 44.1$, df = 5 ($P < 0.0001$); $I^2 = 88.7\%$				
**Subgroup = Regora**				
Cheon et al. 2025	40	40	5.2%	1.00 [0.91; 1.00]
Lee et al. 2025	34	36	5.1%	0.94 [0.81; 0.99]
Piusieux et al. 2025	27	29	4.9%	0.93 [0.77; 0.99]
**Total (95% CI)**		**105**	**15.3%**	**0.97 [0.90; 1.00]**
Heterogeneity: $Tau^2 = 0.0069$; $Chi^2 = 3.94$, df = 2 ($P = 0.1396$); $I^2 = 49.2\%$				
**Total (95% CI)**		**1186**	**100.0%**	**0.88 [0.80; 0.93]**
**Prediction interval**				**[0.50; 1.00]**
Heterogeneity: $Tau^2 = 0.0358$; $Chi^2 = 170.09$, df = 19 ($P < 0.0001$); $I^2 = 88.8\%$				
Test for subgroup differences: $Chi^2 = 6.10$, df = 2 ($P = 0.0473$)				

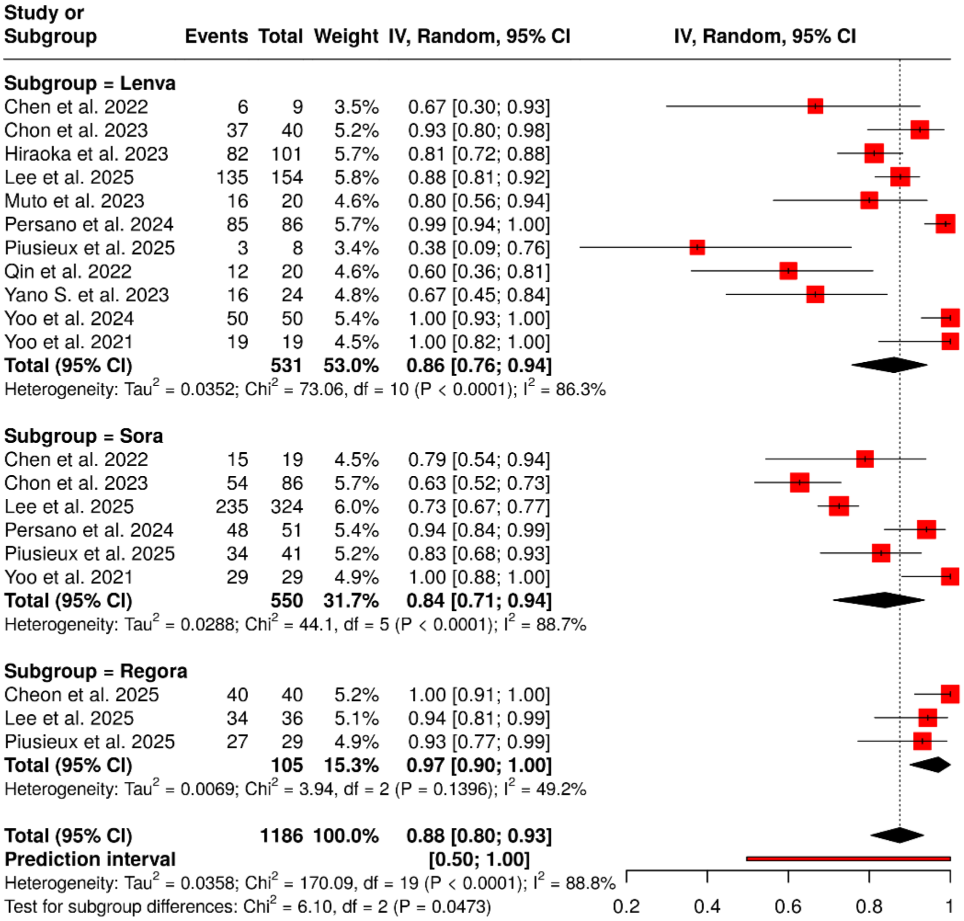

c) Percentage of patients with ***BCLC stage C.*** *No difference.*

Study or Subgroup	Events	Total	Weight	IV, Random, 95% CI
**Subgroup = Lenva**				
Chon et al. 2023	36	40	5.0%	0.90 [0.76; 0.97]
Hiraoka et al. 2023	65	101	5.6%	0.64 [0.54; 0.74]
Lee et al. 2025	126	154	5.7%	0.82 [0.75; 0.88]
Lombardi et al. 2025	56	125	5.7%	0.45 [0.36; 0.54]
Muto et al. 2023	14	20	4.3%	0.70 [0.46; 0.88]
Persano et al. 2024	53	86	5.5%	0.62 [0.51; 0.72]
Piusieux et al. 2025	6	8	3.1%	0.75 [0.35; 0.97]
Qin et al. 2022	20	20	4.3%	1.00 [0.83; 1.00]
Yano S. et al. 2023	16	24	4.5%	0.67 [0.45; 0.84]
Yoo et al. 2024	38	50	5.2%	0.76 [0.62; 0.87]
Yoo et al. 2021	19	19	4.3%	1.00 [0.82; 1.00]
**Total (95% CI)**		**647**	**53.3%**	**0.78 [0.66; 0.88]**
Heterogeneity: Tau^2 = 0.0381; Chi^2 = 94.53, df = 10 (P < 0.0001); I^2 = 89.4%				
**Subgroup = Sora**				
Chon et al. 2023	73	86	5.5%	0.85 [0.76; 0.92]
Lee et al. 2025	246	324	5.9%	0.76 [0.71; 0.80]
Lombardi et al. 2025	71	105	5.6%	0.68 [0.58; 0.76]
Persano et al. 2024	34	51	5.2%	0.67 [0.52; 0.79]
Piusieux et al. 2025	39	41	5.0%	0.95 [0.83; 0.99]
Yoo et al. 2021	19	29	4.7%	0.66 [0.46; 0.82]
**Total (95% CI)**		**636**	**32.0%**	**0.77 [0.68; 0.85]**
Heterogeneity: Tau^2 = 0.0107; Chi^2 = 23.68, df = 5 (P = 0.0003); I^2 = 78.9%				
**Subgroup = Regora**				
Cheon et al. 2025	39	40	5.0%	0.97 [0.87; 1.00]
Lee et al. 2025	12	36	4.9%	0.33 [0.19; 0.51]
Piusieux et al. 2025	27	29	4.7%	0.93 [0.77; 0.99]
**Total (95% CI)**		**105**	**14.7%**	**0.80 [0.33; 1.00]**
Heterogeneity: Tau^2 = 0.1710; Chi^2 = 50.18, df = 2 (P < 0.0001); I^2 = 96%				
**Total (95% CI)**		**1388**	**100.0%**	**0.78 [0.70; 0.85]**
**Prediction interval**				**[0.40; 1.00]**
Heterogeneity: Tau^2 = 0.0309; Chi^2 = 174.65, df = 19 (P < 0.0001); I^2 = 89.1%				
Test for subgroup differences: Chi^2 = 0.01, df = 2 (P = 0.9941)				

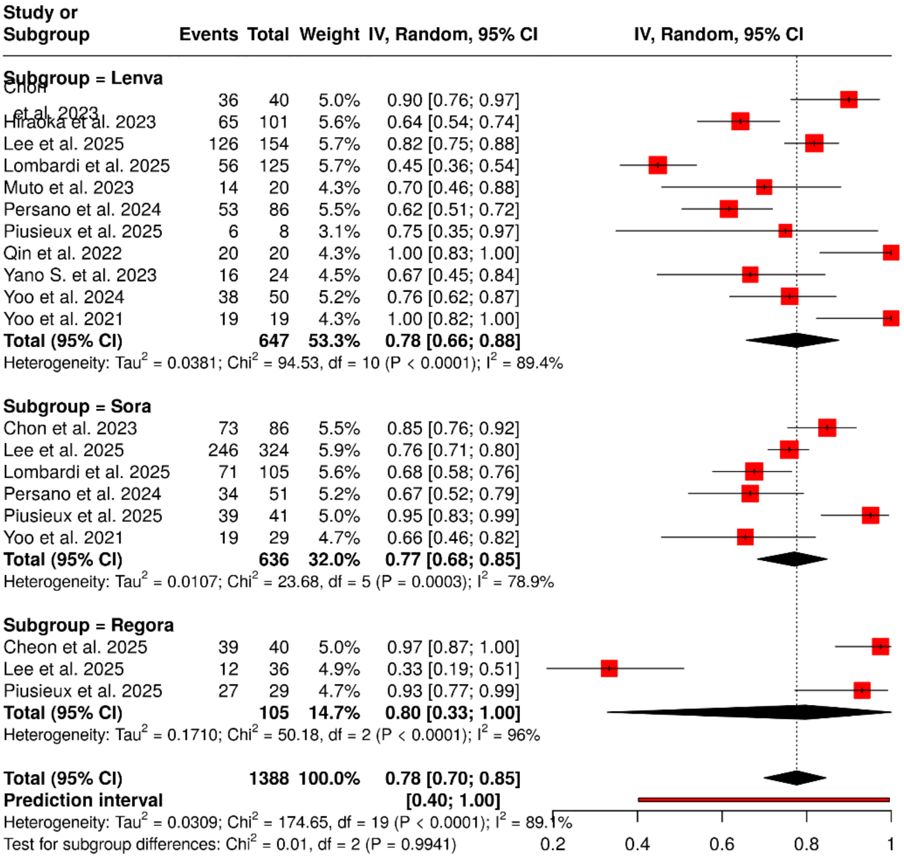

d) Percentage of patients with *ALBI grade 1. No difference.*

Study or Subgroup	Events	Total	Weight	IV, Random, 95% CI
**Subgroup = Lenva**				
Chen et al. 2022	1	9	7.4%	0.11 [0.00; 0.48]
Hiraoka et al. 2023	24	101	8.8%	0.24 [0.16; 0.33]
Lombardi et al. 2025	92	125	8.8%	0.74 [0.65; 0.81]
Muto et al. 2023	6	20	8.1%	0.30 [0.12; 0.54]
Persano et al. 2024	82	86	8.8%	0.95 [0.89; 0.99]
Piusieux et al. 2025	1	8	7.2%	0.12 [0.00; 0.53]
**Total (95% CI)**		**349**	**49.1%**	**0.44 [0.14; 0.77]**
Heterogeneity: $Tau^2 = 0.1545$; $Chi^2 = 162.59$, df = 5 ($P < 0.0001$); $I^2 = 96.9\%$				
**Subgroup = Sora**				
Chen et al. 2022	5	19	8.1%	0.26 [0.09; 0.51]
Lombardi et al. 2025	49	105	8.8%	0.47 [0.37; 0.57]
Persano et al. 2024	49	51	8.6%	0.96 [0.87; 1.00]
Piusieux et al. 2025	6	41	8.5%	0.15 [0.06; 0.29]
**Total (95% CI)**		**216**	**34.0%**	**0.48 [0.12; 0.85]**
Heterogeneity: $Tau^2 = 0.1571$; $Chi^2 = 94.21$, df = 3 ($P < 0.0001$); $I^2 = 96.8\%$				
**Subgroup = Regora**				
Cheon et al. 2025	17	40	8.5%	0.42 [0.27; 0.59]
Piusieux et al. 2025	6	29	8.4%	0.21 [0.08; 0.40]
**Total (95% CI)**		**69**	**16.9%**	**0.32 [0.13; 0.54]**
Heterogeneity: $Tau^2 = 0.0187$; $Chi^2 = 3.55$, df = 1 ($P = 0.0595$); $I^2 = 71.8\%$				
**Total (95% CI)**		**634**	**100.0%**	**0.44 [0.24; 0.64]**
**Prediction interval**				**[0.00; 1.00]**
Heterogeneity: $Tau^2 = 0.1206$; $Chi^2 = 280.17$, df = 11 ($P < 0.0001$); $I^2 = 96.1\%$				
Test for subgroup differences: $Chi^2 = 0.71$, df = 2 ($P = 0.7023$)				

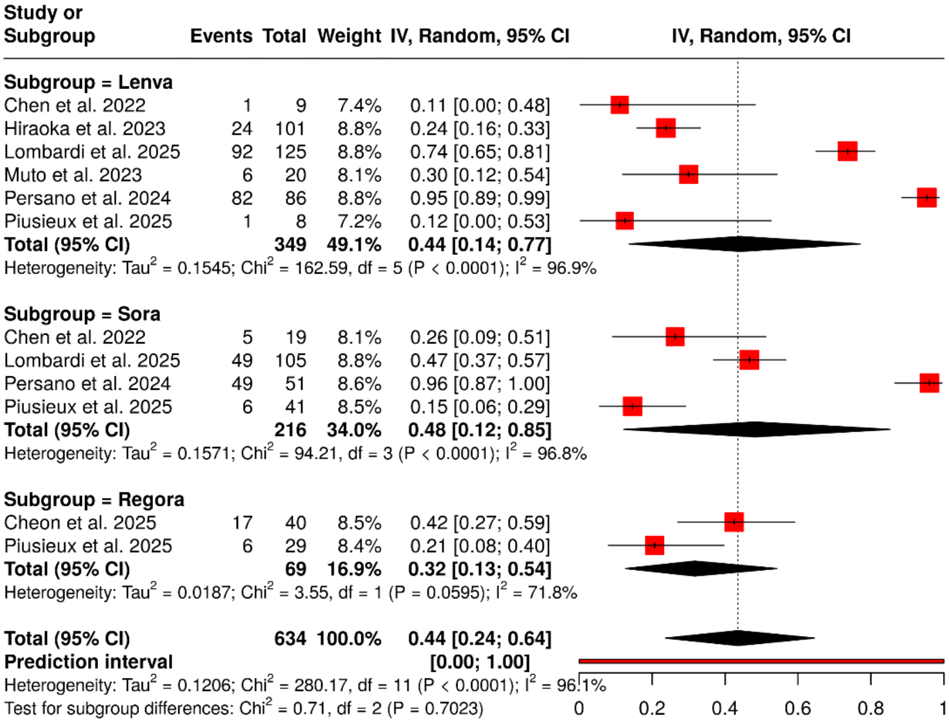

e) Percentage of patients with *macrovascular invasion (MVI).* *The regorafenib group had significantly less MVI.*

Study or Subgroup	Events	Total	Weight	IV, Random, 95% CI
**Subgroup = Lenva**				
Chen et al. 2022	9	9	3.4%	1.00 [0.66; 1.00]
Chon et al. 2023	17	40	6.6%	0.42 [0.27; 0.59]
Hiraoka et al. 2023	30	101	7.9%	0.30 [0.21; 0.40]
Lee et al. 2025	42	154	8.3%	0.27 [0.20; 0.35]
Piusieux et al. 2025	2	8	3.2%	0.25 [0.03; 0.65]
Yano S. et al. 2023	8	24	5.6%	0.33 [0.16; 0.55]
Yoo et al. 2024	12	50	7.0%	0.24 [0.13; 0.38]
Yoo et al. 2021	9	19	5.1%	0.47 [0.24; 0.71]
**Total (95% CI)**		**405**	**47.0%**	**0.38 [0.27; 0.51]**
Heterogeneity: $Tau^2 = 0.0201$; $Chi^2 = 32.09$, df = 7 (P < 0.0001); $I^2 = 78.2\%$				
**Subgroup = Sora**				
Chen et al. 2022	14	19	5.1%	0.74 [0.49; 0.91]
Chon et al. 2023	41	86	7.7%	0.48 [0.37; 0.59]
Lee et al. 2025	90	324	8.7%	0.28 [0.23; 0.33]
Piusieux et al. 2025	20	41	6.6%	0.49 [0.33; 0.65]
Yoo et al. 2021	9	29	6.0%	0.31 [0.15; 0.51]
**Total (95% CI)**		**499**	**34.1%**	**0.44 [0.29; 0.59]**
Heterogeneity: $Tau^2 = 0.0227$; $Chi^2 = 28.55$, df = 4 (P < 0.0001); $I^2 = 86\%$				
**Subgroup = Regora**				
Cheon et al. 2025	10	40	6.6%	0.25 [0.13; 0.41]
Lee et al. 2025	5	36	6.4%	0.14 [0.05; 0.29]
Piusieux et al. 2025	8	29	6.0%	0.28 [0.13; 0.47]
**Total (95% CI)**		**105**	**18.9%**	**0.22 [0.14; 0.31]**
Heterogeneity: $Tau^2 = 0.0006$; $Chi^2 = 2.17$, df = 2 (P = 0.3387); $I^2 = 7.6\%$				
**Total (95% CI)**		**1009**	**100.0%**	**0.36 [0.29; 0.44]**
**Prediction interval**				**[0.12; 0.65]**
Heterogeneity: $Tau^2 = 0.0158$; $Chi^2 = 69.43$, df = 15 (P < 0.0001); $I^2 = 78.4\%$				
Test for subgroup differences: $Chi^2 = 8.75$, df = 2 (P = 0.0126)				

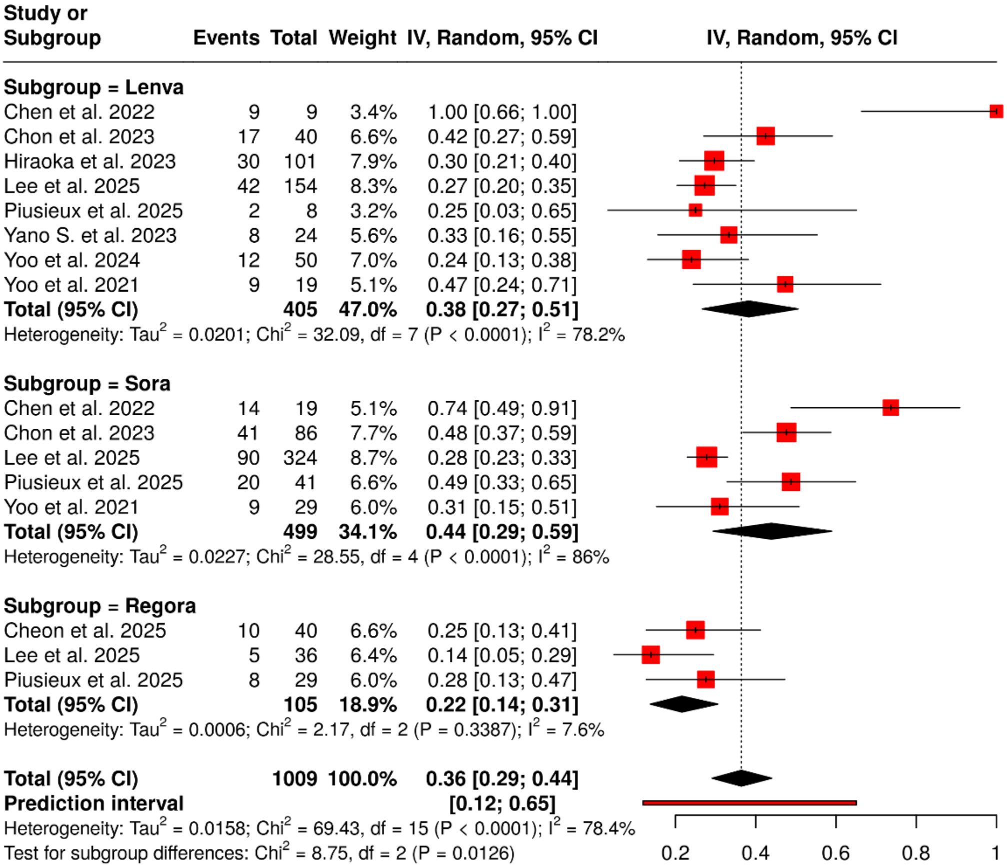

f) Percentage of patients with ***extrahepatic spread.*** *No difference.*

Study or Subgroup	Events	Total	Weight	IV, Random, 95% CI
**Subgroup = Lenva**				
Chen et al. 2022	5	9	3.2%	0.56 [0.21; 0.86]
Chon et al. 2023	29	40	6.1%	0.72 [0.56; 0.85]
Hiraoka et al. 2023	45	101	7.2%	0.45 [0.35; 0.55]
Lee et al. 2025	106	154	7.6%	0.69 [0.61; 0.76]
Lombardi et al. 2025	60	125	7.4%	0.48 [0.39; 0.57]
Muto et al. 2023	9	20	4.8%	0.45 [0.23; 0.68]
Piusieux et al. 2025	4	8	3.0%	0.50 [0.16; 0.84]
Qin et al. 2022	11	20	4.8%	0.55 [0.32; 0.77]
Yano S. et al. 2023	11	24	5.2%	0.46 [0.26; 0.67]
**Total (95% CI)**		**501**	**49.4%**	**0.55 [0.45; 0.64]**
Heterogeneity: Tau^2 = 0.0111; Chi^2 = 25.82, df = 8 (P = 0.0011); I^2 = 69%				
**Subgroup = Sora**				
Chen et al. 2022	13	19	4.7%	0.68 [0.43; 0.87]
Chon et al. 2023	58	86	7.1%	0.67 [0.56; 0.77]
Lee et al. 2025	193	324	7.9%	0.60 [0.54; 0.65]
Lombardi et al. 2025	47	105	7.3%	0.45 [0.35; 0.55]
Piusieux et al. 2025	34	41	6.1%	0.83 [0.68; 0.93]
**Total (95% CI)**		**575**	**33.1%**	**0.64 [0.52; 0.75]**
Heterogeneity: Tau^2 = 0.0129; Chi^2 = 22.57, df = 4 (P = 0.0002); I^2 = 82.3%				
**Subgroup = Regora**				
Cheon et al. 2025	34	40	6.1%	0.85 [0.70; 0.94]
Lee et al. 2025	11	36	5.9%	0.31 [0.16; 0.48]
Piusieux et al. 2025	25	29	5.5%	0.86 [0.68; 0.96]
**Total (95% CI)**		**105**	**17.5%**	**0.69 [0.31; 0.97]**
Heterogeneity: Tau^2 = 0.1057; Chi^2 = 31.78, df = 2 (P < 0.0001); I^2 = 93.7%				
**Total (95% CI)**		**1181**	**100.0%**	**0.60 [0.53; 0.68]**
**Prediction interval**				**[0.31; 0.86]**
Heterogeneity: Tau^2 = 0.0169; Chi^2 = 86.06, df = 16 (P < 0.0001); I^2 = 81.4%				
Test for subgroup differences: Chi^2 = 1.75, df = 2 (P = 0.4164)				

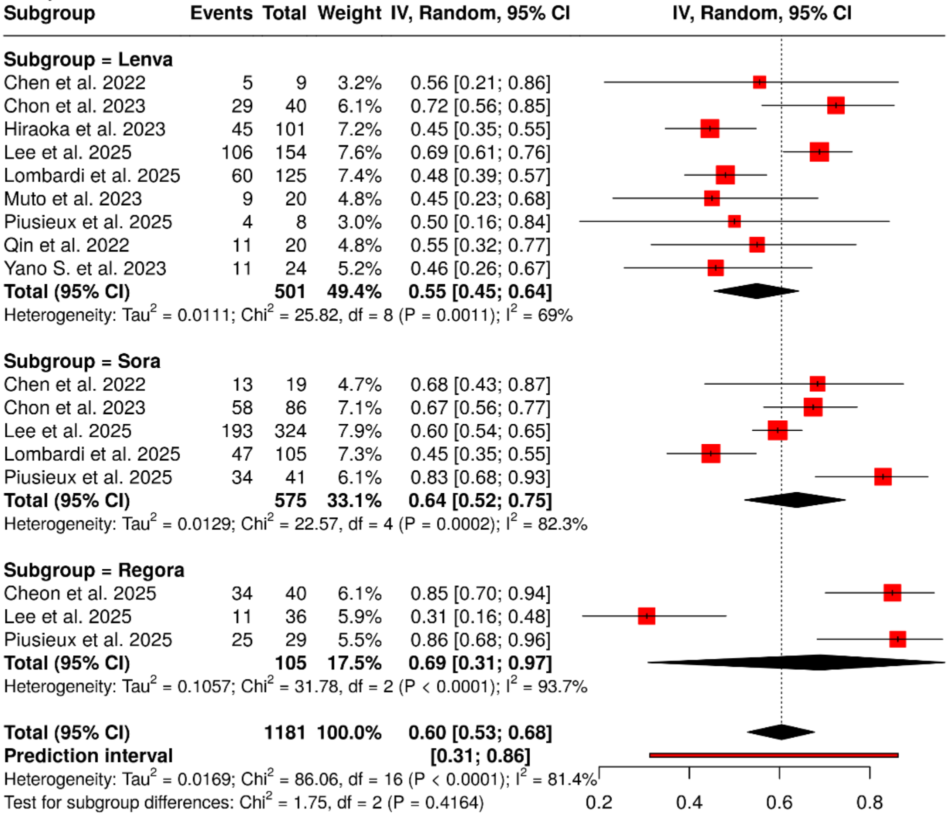

Fig. S3. Pooled baseline characteristics and comparison between Lenva (lenvatinib), Sora (sorafenib), and Regora (regorafenib). a*) European Cooperation Oncology Group performance score (ECOG-PS) 0, b) Child-Pugh (CP) A, c) Barcelona Clinic Liver Cancer (BCLC) C, d) ALBI grade 1, e) Macrovascular invasion (MVI), f) Extrahepatic spread*

OS

Chisq: 17.7

df: 2

p: 0.00014

PFS

Chisq: 28.8

df: 2

p: 5.7e-07

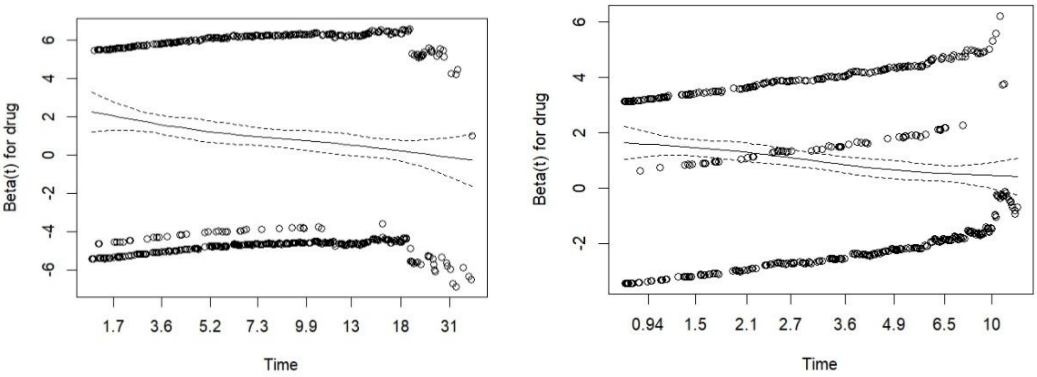

Fig. S4. Proportional hazards assumption, Schoenfeld residuals testing for OS and PFS among lenvatinib, sorafenib and regorafenib

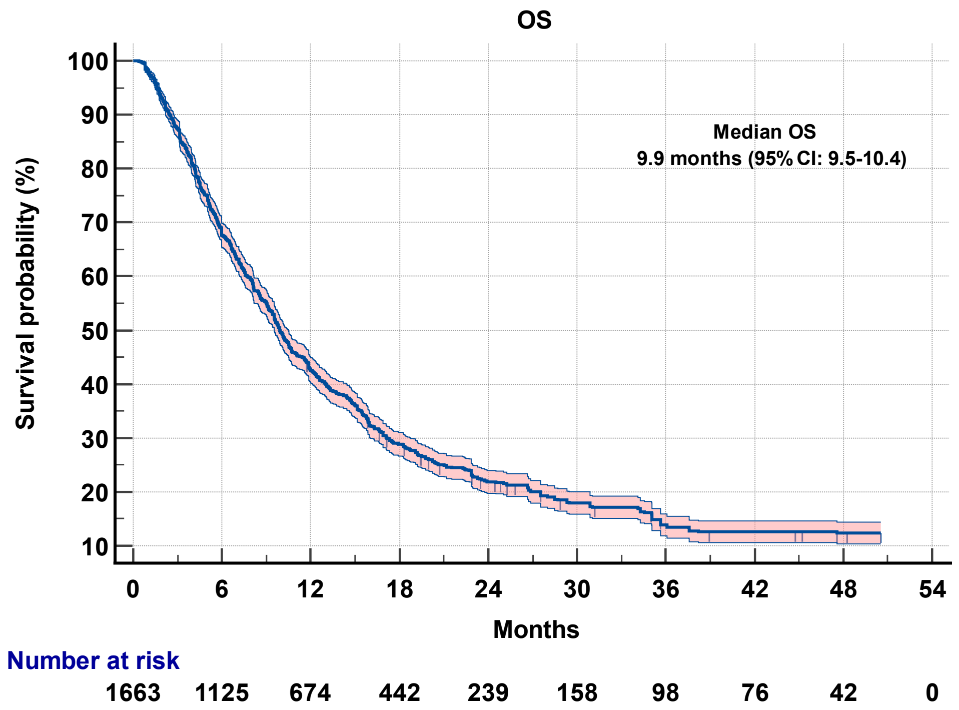

Fig. S5. Pooled overall survival (OS) with second-line tyrosine kinase inhibitors (TKIs) (lenvatinib, sorafenib, regorafenib, cabozantinib) in advanced hepatocellular carcinoma (HCC)

a)

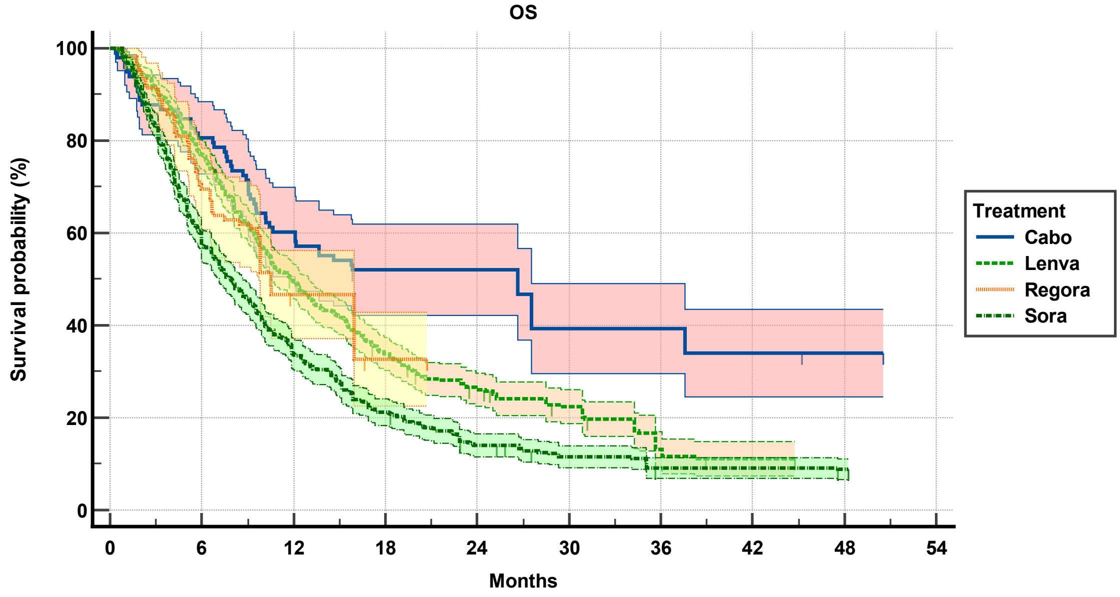

Number at risk

Group	0	6	12	18	24	30	36	42	48	54
Group: Cabo	98	79	59	49	49	37	37	32	20	0
Group: Lenva	691	532	326	221	95	58	26	9	0	0
Group: Regora	105	73	30	9	0	0	0	0	0	0
Group: Sora	769	441	259	163	95	63	35	35	22	0

Treatment	Median OS (months)	95% CI	Comparison	HR	95% CI
**Cabo**	26.6	10.6-27.5	**Lenva vs Sora**	**0.68**	**0.61-0.77**
**Lenva**	11.9	10.5-12.9	**Regora vs Sora**	**0.69**	**0.54-0.89**
**Regora**	10.4	9.6-15.9	**Cabo vs. Sora**	**0.42**	**0.34-0.51**
**Sora**	7.9	7.1-8.6	**Lenva vs. Regora**	0.98	0.77-1.25
**P<0.001**			**Lenva vs. Cabo**	**1.62**	**1.33-1.98**
			**Regora vs Cabo**	**1.65**	**1.23-2.21**

b)

Treatment	RMST 12-month OS (month difference, 95% CI)	*p*	RMST 20-month OS (month difference, 95% CI)	*p*
**Lenva vs Sora**	**1.49 (1.10-1.88)**	**<0.001**	**2.58 (1.90-3.26)**	**<0.001**
**Regora vs Sora**	**1.16 (0.41-1.96)**	**0.002**	**2.30 (0.90-3.70)**	**0.001**
**Cabo vs. Sora**	**1.88 (1.09-2.66)**	**<0.001**	**4.15 (2.67-5.62)**	**<0.001**
**Lenva vs. Regora**	0.32 (-0.42-1.07)	0.393	0.27 (-1.12-1.68)	0.697
**Lenva vs. Cabo**	-0.38 (-1.16- 0.39)	0.332	**-1.57 (-3.05- -0.08)**	**0.037**
**Regora vs Cabo**	-0.71 (-1.73-0.30)	0.169	-1.85 (-3.77- 0.07)	0.059

Fig. S6. a) Comparison of OS across TKIs in the second-line treatment of advanced HCC b) RMST analyses for 12-month and 20-month OS. OS: Overall survival, Cabo: cabozantinib, Lenva: Lenvatinib, Regora: Regorafenib, Sora: Sorafenib, CI: confidence interval, HR: Hazard ratio, RMST: Restricted mean survival time, TKIs: tyrosine kinase inhibitors

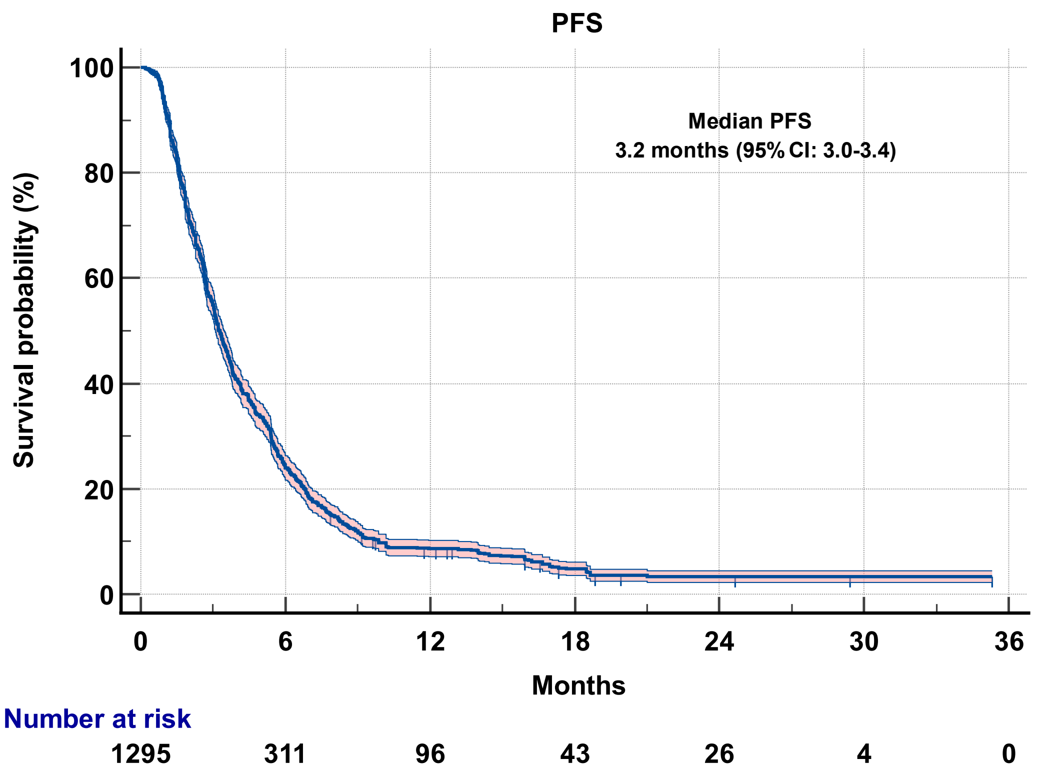

Fig. S7. Pooled progression-free survival (PFS) with second-line tyrosine kinase inhibitors (TKIs) in advanced hepatocellular carcinoma (HCC)

a)

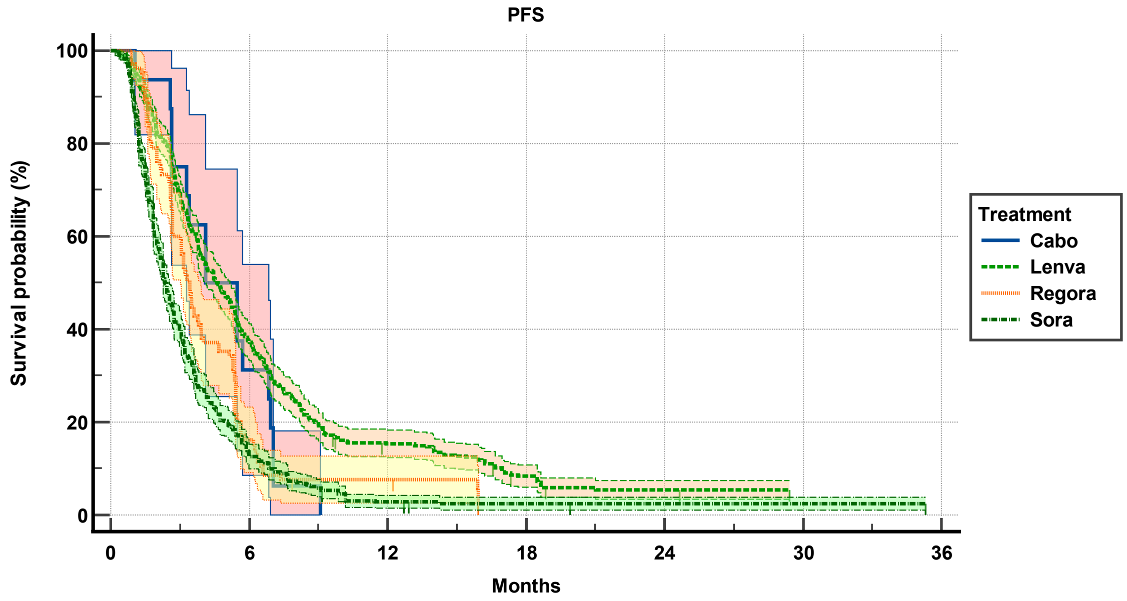

Number at risk

Group: Cabo

16	5	0	0	0	0	0

Group: Lenva

570	213	75	37	22	0	0

Group: Regora

105	17	6	0	0	0	0

Group: Sora

604	76	15	6	4	4	0

Treatment	Median PFS (months)	95% CI	Comparison	HR	95% CI
**Cabo**	4.1	2.6-6.8	**Lenva vs Sora**	**0.51**	**0.45-0.58**
**Lenva**	4.5	4.0-5.1	**Regora vs Sora**	**0.76**	**0.61-0.96**
**Regora**	3.4	2.7-3.9	Cabo vs. Sora	0.63	0.39-1.02
**Sora**	2.3	2.2-2.5	**Lenva vs. Regora**	**0.66**	**0.53-0.83**
**P<0.001**			Lenva vs. Cabo	0.80	0.50-1.30
			Regora vs Cabo	1.21	0.72-2.01

b)

Treatment	RMST 6-month PFS (month difference, 95% CI)	*p*	RMST 9-month PFS (month difference, 95% CI)	*p*
**Lenva vs Sora**	**1.28 (1.08-1.49)**	**<0.001**	**1.84 (1.54-2.13)**	**<0.001**
**Regora vs Sora**	**0.73 (0.38-1.09)**	**<0.001**	**0.74 (0.28-1.19)**	**0.001**
**Cabo vs. Sora**	**1.48 (0.69-2.27)**	**<0.001**	**1.64 (0.59-2.69)**	**0.002**
**Lenva vs. Regora**	**0.54 (0.19-0.90)**	**0.002**	**1.10 (0.62-1.57)**	**<0.001**
**Lenva vs. Cabo**	-0.19 (-0.98- 0.58)	0.622	0.19 (-0.86-1.25)	0.718
**Regora vs Cabo**	-0.74 (-1.58-0.09)	0.080	-0.90 (-2.01- 0.20)	0.110

Fig. S8. a) Comparison of PFS across TKIs in the second-line treatment of advanced HCC b) RMST analyses for 6-month and 9-month PFS. PFS: Progression-free survival, Cabo: cabozantinib, Lenva: Lenvatinib, Regora: Regorafenib, Sora: Sorafenib, CI: confidence interval, HR: Hazard ratio, RMST: Restricted mean survival time, TKIs: tyrosine kinase inhibitors

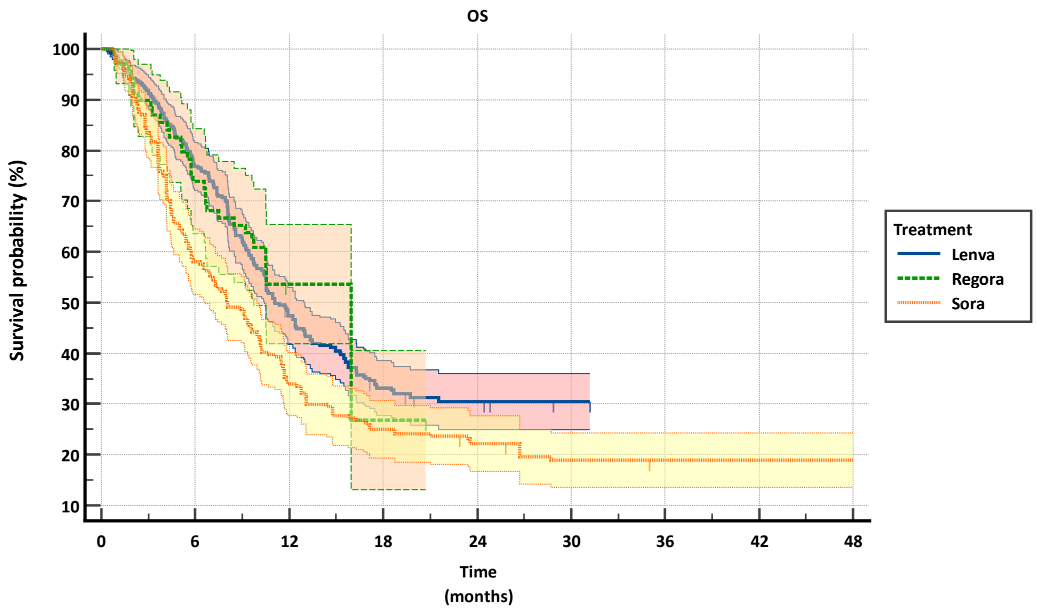

Number at risk

**Group: Lenva**									
	307	236	130	89	38	12	0	0	0
**Group: Regora**									
	69	51	18	9	0	0	0	0	0
**Group: Sora**									
	224	130	76	56	45	29	22	22	22

Treatment	Median OS (months)	95% CI	Comparison	HR	95% CI
**Lenva**	11.0	10.3-12.9	**Lenva vs Sora**	**0.70**	**0.57-0.86**
**Regora**	15.9	NC	**Regora vs Sora**	**0.68**	**0.49-0.95**
**Sora**	8	6.5-9.9	**Lenva vs. Regora**	1.01	0.74-1.39

Treatment	RMST 12-month OS (month difference, 95% CI)	*p*	RMST 20-month OS (month difference, 95% CI)	*p*
**Lenva vs Sora**	**1.42 (0.77-2.07)**	**<0.001**	**2.24 (1.08-3.39)**	**<0.001**
**Regora vs Sora**	**1.39 (0.37-2.40)**	**0.007**	**2.38 (0.56-4.20)**	**0.010**
**Lenva vs. Regora**	0.02 (-0.92-0.98)	0.951	-0.14 (-1.89-1.60)	0.870

Fig. S9. OS sensitivity analyses by excluding multicountry studies. a) Comparison of OS across TKIs in the second-line treatment of advanced HCC b) RMST analyses for 12-month and 20-month OS. OS: Overall survival, Cabo: cabozantinib, Lenva: Lenvatinib, Regora: Regorafenib, Sora: Sorafenib, CI: confidence interval, HR: Hazard ratio, RMST: Restricted mean survival time, TKIs: tyrosine kinase inhibitors

a) Hand-foot syndrome (any grade)

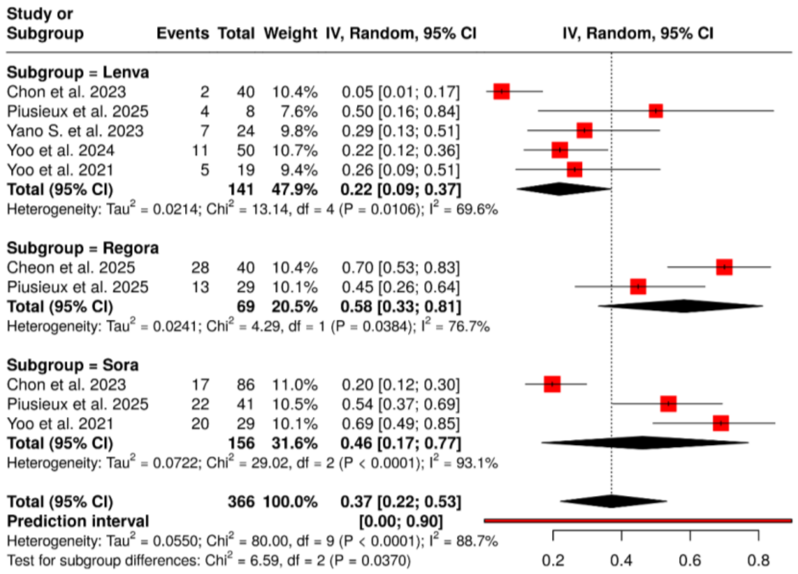

b) Fatigue (any grade)

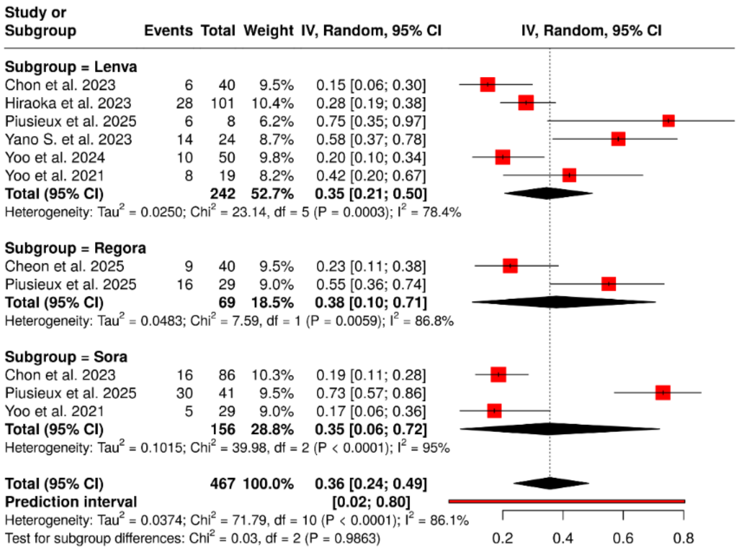

c) Hypertension (Any grade)

Study or Subgroup	Events	Total	Weight	IV, Random, 95% CI
**Subgroup = Lenva**				
Chon et al. 2023	17	40	9.5%	0.42 [0.27; 0.59]
Hiraoka et al. 2023	19	101	10.7%	0.19 [0.12; 0.28]
Piusieux et al. 2025	2	8	5.7%	0.25 [0.03; 0.65]
Yano S. et al. 2023	15	24	8.6%	0.62 [0.41; 0.81]
Yoo et al. 2024	11	50	9.9%	0.22 [0.12; 0.36]
Yoo et al. 2021	8	19	8.0%	0.42 [0.20; 0.67]
**Total (95% CI)**		**242**	**52.4%**	**0.34 [0.20; 0.49]**
Heterogeneity: $Tau^2 = 0.0243$; $Chi^2 = 22.65$, df = 5 (P = 0.0004); $I^2 = 77.9\%$				
**Subgroup = Regora**				
Cheon et al. 2025	6	40	9.5%	0.15 [0.06; 0.30]
Piusieux et al. 2025	1	29	9.0%	0.03 [0.00; 0.18]
**Total (95% CI)**		**69**	**18.5%**	**0.09 [0.01; 0.23]**
Heterogeneity: $Tau^2 = 0.0101$; $Chi^2 = 2.38$, df = 1 (P = 0.1226); $I^2 = 58.1\%$				
**Subgroup = Sora**				
Chon et al. 2023	5	86	10.5%	0.06 [0.02; 0.13]
Piusieux et al. 2025	7	41	9.6%	0.17 [0.07; 0.32]
Yoo et al. 2021	5	29	9.0%	0.17 [0.06; 0.36]
**Total (95% CI)**		**156**	**29.1%**	**0.12 [0.04; 0.22]**
Heterogeneity: $Tau^2 = 0.0089$; $Chi^2 = 5.32$, df = 2 (P = 0.0699); $I^2 = 62.4\%$				
**Total (95% CI)**		**467**	**100.0%**	**0.22 [0.13; 0.32]**
**Prediction interval**				**[0.00; 0.61]**
Heterogeneity: $Tau^2 = 0.0286$; $Chi^2 = 57.18$, df = 10 (P < 0.0001); $I^2 = 82.5\%$				
Test for subgroup differences: $Chi^2 = 9.30$, df = 2 (P = 0.0096)				

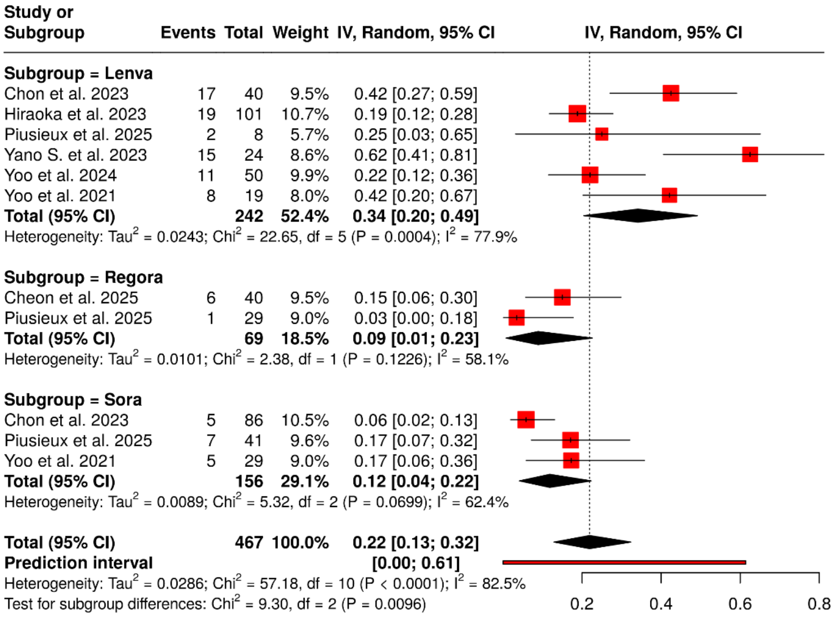

d) Diarrhea (Any grade)

Study or Subgroup	Events	Total	Weight	IV, Random, 95% CI
**Subgroup = Lenva**				
Chon et al. 2023	16	40	11.3%	0.40 [0.25; 0.57]
Piusieux et al. 2025	1	8	3.3%	0.12 [0.00; 0.53]
Yano S. et al. 2023	10	24	7.9%	0.42 [0.22; 0.63]
Yoo et al. 2024	16	50	12.9%	0.32 [0.20; 0.47]
Yoo et al. 2021	5	19	6.7%	0.26 [0.09; 0.51]
**Total (95% CI)**		**141**	**42.1%**	**0.34 [0.26; 0.42]**
Heterogeneity: $Tau^2 = 0$; $Chi^2 = 3.25$, df = 4 (P = 0.5174); $I^2 = 0\%$				
**Subgroup = Regora**				
Cheon et al. 2025	6	40	11.3%	0.15 [0.06; 0.30]
Piusieux et al. 2025	4	29	9.1%	0.14 [0.04; 0.32]
**Total (95% CI)**		**69**	**20.3%**	**0.14 [0.07; 0.24]**
Heterogeneity: $Tau^2 = 0$; $Chi^2 = 0.01$, df = 1 (P = 0.9204); $I^2 = 0\%$				
**Subgroup = Sora**				
Chon et al. 2023	20	86	17.1%	0.23 [0.15; 0.34]
Piusieux et al. 2025	10	41	11.4%	0.24 [0.12; 0.40]
Yoo et al. 2021	6	29	9.1%	0.21 [0.08; 0.40]
**Total (95% CI)**		**156**	**37.6%**	**0.23 [0.17; 0.30]**
Heterogeneity: $Tau^2 = 0$; $Chi^2 = 0.11$, df = 2 (P = 0.9474); $I^2 = 0\%$				
**Total (95% CI)**		**366**	**100.0%**	**0.25 [0.19; 0.31]**
**Prediction interval**				**[0.12; 0.40]**
Heterogeneity: $Tau^2 = 0.0035$; $Chi^2 = 13.49$, df = 9 (P = 0.1417); $I^2 = 33.3\%$				
Test for subgroup differences: $Chi^2 = 10.12$, df = 2 (P = 0.0063)				

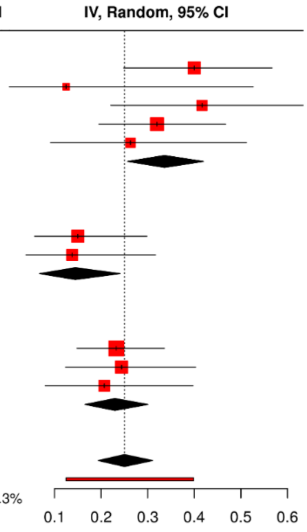

e) Proteinuria with lenvatinib (Any grade)

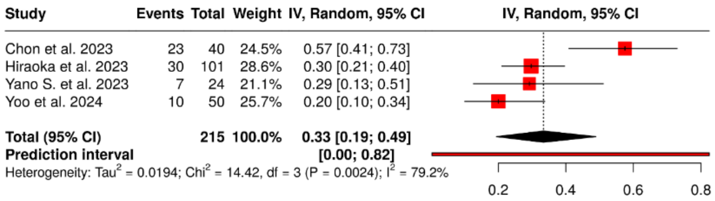

Fig. S10. Pooled and comparative analyses of adverse events.

Supplementary tables

Table S1. Risk of bias assessment of treatment arms (ROB-ME tool and modified Cowley’s criteria scoring)

Study	Treatment	Number of participants analysed	Result available for OS	Result available for PFS	Result available for ECOG-0	Result available for CP-A	Result available for BCLC-C	Result available for ALBI Grade 1	Result available for MVI	Result available for extrahepatic spread	Result available for toxicities	Result available for response	Modified Cowley’s criteria score	Risk group modified Cowley’s criteria
**Chen et al, 2022**	Lenva	9	✓	✓	~	✓	~	✓	✓	✓	~	✓	21	Moderate
**Chon et al, 2023**	Lenva	40	✓	✓	✓	✓	✓	~	✓	✓	✓	✓	24	Low
**Decreacker et al, 2025**	Lenva	35	✓	~	~	~	~	~	~	~	~	~	22	Moderate
**Hiraoka,2023**	Lenva	101	✓	✓	✓	✓	✓	✓	✓	✓	✓	✓	22	Moderate
**Lee et al, 2025**	Lenva	154	✓	✓	✓	✓	✓	~	✓	✓	~	✓	22	Moderate
**Lombardi et al, 2025**	Lenva	125	✓	✓	✓	~	✓	✓	~	✓	~	~	21	Moderate
**Muto et al, 2023**	Lenva	20	✓	✓	✓	✓	✓	✓	~	✓	~	✓	21	Moderate
**Persano et al, 2024**	Lenva	86	✓	~	~	✓	✓	✓	~	~	~	✓	20	Moderate
**Piusieux et al, 2025**	Lenva	8	✓	✓	✓	✓	✓	✓	✓	✓	✓	~	23	Moderate
**Qin et al, 2022**	Lenva	20	✓	✓	~	✓	✓	~	~	✓	~	✓	19	High
**Yano S. et al, 2023**	Lenva	24	✓	✓	✓	✓	✓	~	✓	✓	✓	✓	22	Moderate
**Yoo et al, 2024**	Lenva	50	✓	✓	✓	✓	✓	~	✓	~	✓	✓	25	Low
**Yoo et al, 2021**	Lenva	19	✓	✓	✓	✓	✓	~	✓	~	✓	✓	25	Low
**Chen et al, 2022**	Sora	19	✓	✓	~	✓	~	~	✓	✓	~	✓	22	Moderate
**Chon et al, 2023**	Sora	86	✓	✓	✓	✓	✓	~	✓	✓	✓	✓	24	Low
**Decreacker et al, 2025**	Sora	78	✓	~	~	~	~	~	~	~	~	~	22	Moderate
**Lee et al, 2025**	Sora	324	✓	✓	✓	✓	✓	~	✓	✓	~	✓	22	Moderate
**Lombardi et al, 2025**	Sora	105	✓	✓	✓	~	✓	✓	~	✓	~	~	22	Moderate
**Möhring et al, 2025**	Sora	36	✓	~	~	~	~	~	~	~	~	~	20	Moderate
**Persano et al, 2024**	Sora	51	✓	~	~	✓	✓	✓	~	~	~	✓	20	Moderate
**Piusieux et al, 2025**	Sora	41	✓	✓	✓	✓	✓	✓	✓	✓	✓	~	23	Moderate
**Yoo et al, 2021**	Sora	29	✓	✓	✓	✓	✓	~	✓	~	✓	✓	25	Low
**Cheon et al., 2025**	Regora	40	✓	✓	✓	✓	✓	✓	✓	✓	✓	✓	25	Low
**Lee et al, 2025**	Regora	36	✓	✓	✓	✓	✓	~	✓	✓	~	✓	22	Moderate
**Piusieux et al, 2025**	Regora	29	✓	✓	✓	✓	✓	✓	✓	✓	✓	~	23	Moderate
**Ahn et al., 2025 (1L only IO)**	Cabo	28	✓	?	~	~	~	~	~	~	~	~	18	High
**Ahn et al., 2025 (1KL IO-antiVEGF)**	Cabo	54	✓	?	~	~	~	~	~	~	~	~	18	High
**Lee et al, 2025**	Cabo	12	✓	✓	✓	✓	✓	~	✓	✓	~	✓	22	Moderate
**Piusieux et al, 2025**	Cabo	4	✓	✓	✓	✓	✓	✓	✓	✓	✓	~	23	Moderate
Risk of bias judgment	-	-	***Low***	***Some concern***	***Some concern***	***Some concern***	***Some concern***	***Some concern***	***Some concern***	***Some concern***	***High***	***High***	**-**	**-**

✓: A study result is available for inclusion in the meta-analysis, ~No study result is available for inclusion in the meta-analysis, for a reason unrelated to the P value, magnitude or direction of the result, ?: Unclear whether an eligible study result was generated, X: No study result is available for inclusion in the meta-analysis, likely because of the P value, magnitude or direction of the result generated

OS: Overall survival, PFS: Progression-free survival, Lenva: Lenvatinib, Sora: Sorafenib, Regora: Regorafenib, Cabo: Cabozantinib, ECOG-PS: European Cooperation Oncology Group performance score, CP: Child-Pugh, c) BCLC: Barcelona Clinic Liver Cancer, MVI: Macrovascular invasion

Table S2. Adverse events (any grade, observed in >5% of the patients) with the second-line tyrosine kinase inhibitors (TKIs)

Study	Year	Treatment	Sample size	Hand-foot syndrome	Anorexia	Fatigue	Hyperbilirubinemia	AST increased	Nausea	Thrombocytopenia	Hypertension	Diarrhea	Proteinuria	Pruritus	ALT increased	Hypothyroidism	Anemia	Oral mucositis	Neutropenia	GI bleeding	Constipation
**Piusieux et al.**	2025	Cabo	4	1	NA	2	NA	NA	0	NA	1	1	NA	NA	NA	NA	NA	NA	NA	NA	NA
**Chon et al.**	2023	Lenva	40	2	16	6	16	20	10	20	17	16	23	4	15	14	10	6	4	3	3
**Hiraoka et al.**	2023	Lenva	101	NA	37	28	NA	NA	NA	NA	19	NA	30	NA	NA	NA	NA	NA	NA	NA	NA
**Piusieux et al.**	2025	Lenva	8	4	NA	6	NA	NA	2	NA	2	1	NA	NA	NA	NA	NA	NA	NA	NA	NA
**Yano S. et al.**	2023	Lenva	24	7	11	14	NA	NA		2	15	10	7	NA	NA	5	NA	NA	NA	NA	NA
**Yoo et al.**	2024	Lenva	50	11	15	10	7	5	10	5	11	16	10	6	NA	15	NA	NA	NA	NA	NA
**Yoo et al.**	2021	Lenva	19	5	4	8	2	NA		3	8	5	NA	NA	NA	NA	2	NA	NA	NA	NA
**Cheon et al.**	2025	Regora	40	28	10	9	9	8	7	7	6	6	6	5	5	3	NA	NA	NA	NA	NA
**Piusieux et al.**	2025	Regora	29	13	NA	16	NA	NA	1	NA	1	4	NA	NA	NA	NA	NA	NA	NA	NA	NA
**Chon et al.**	2023	Sora	86	17	17	16	47	45	11	19	5	20	3	6	22	1	8	6	5	3	1
**Piusieux et al.**	2025	Sora	41	22	NA	30	NA	NA	3	NA	7	10	NA	NA	NA	NA	NA	NA	NA	NA	NA
**Yoo et al.**	2021	Sora	29	20	2	5	4	NA		6	5	6	NA	NA	NA	NA	1	NA	NA	NA	NA

NA: Not available, Cabo: Cabozantinib, Lenva: Lenvatinib, Regora: Regorafenib, Sora: Sorafenib
