# Supplementary material for: Second-line TKI after first-line immunotherapy-based treatment in advanced HCC: Reconstructed IPD meta-analysis
Source: JHEP Rep. 2026 May 12;8(8):101893. doi: 10.1016/j.jhepr.2026.101893 (PMC13352052; doi:10.1016/j.jhepr.2026.101893)
Supplement: Multimedia component 4 [file mmc4.pdf]

Research article

JHEP|Reports

Second-line TKI after first-line immunotherapy-based treatment in advanced HCC: Reconstructed IPD meta-analysis

Authors

Erman Akkus, Christian Hobeika, Julien Edeline, ..., Marie Lequoy, Jean Charles Nault, Mohamed Bouattour

Correspondence

mohamed.bouattour@aphp.fr (M. Bouattour).

Graphical abstract

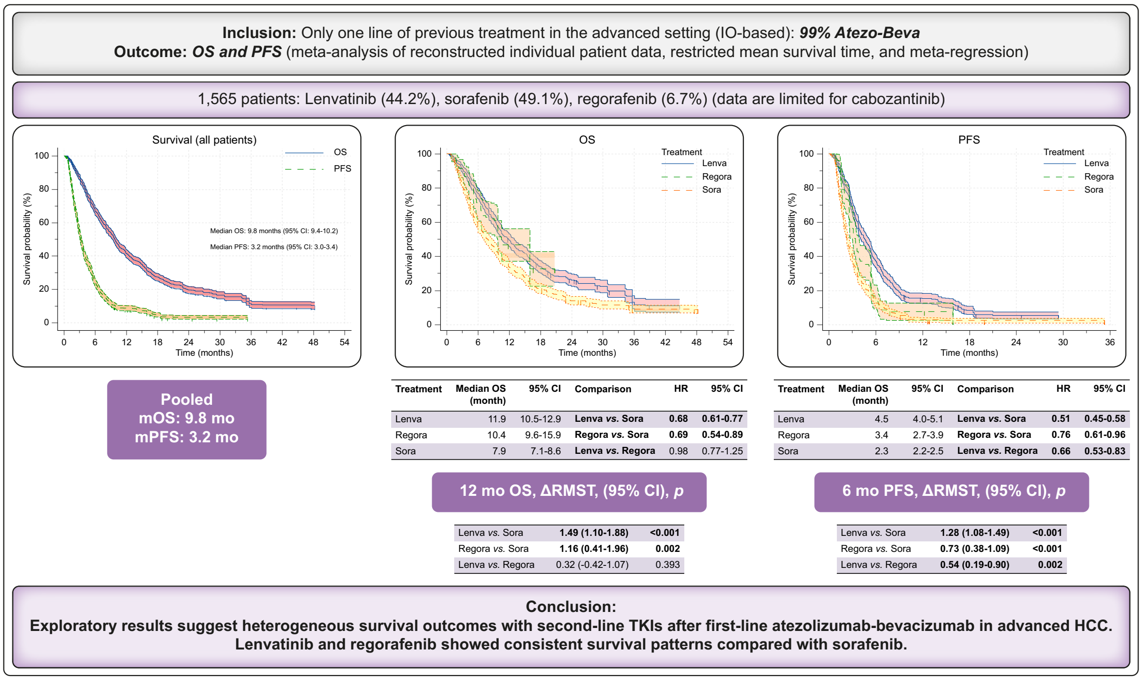

Highlights:

- Real-world evidence comparing 2L TKIs after IO-based 1L treatment in advanced HCC is limited.
- Reconstructed IPD meta-analysis allows time-restricted survival comparisons when proportional hazards are violated.
- Restricted mean survival time analyses showed heterogeneous survival outcomes across 2L TKIs after immunotherapy.
- Lenvatinib and regorafenib showed consistent survival patterns compared with sorafenib in pooled retrospective data.
- Findings are exploratory, prospective studies are needed to define optimal post-immunotherapy sequencing strategies.

Impact and implications:

This study addresses a critical evidence gap in advanced hepatocellular carcinoma by synthesizing available real-world data on second-line TKIs after IO-based first-line treatment, using reconstructed individual patient data and restricted mean survival time to accommodate non-proportional hazards and heterogeneous follow-up. The findings should not be interpreted as a recommendation for any specific TKI. Instead, they describe current survival patterns across heterogeneous retrospective cohorts and underscore the absence of robust comparative evidence in the post-immunotherapy setting. The results are relevant for clinicians and multidisciplinary teams caring for patients who progress after immunotherapy, as they may help contextualize expectations, support shared decision-making with patients and caregivers, and highlight areas of doubt in routine practice. This work highlights the unmet need for prospective randomized trials and high-quality real-world registries to define optimal treatment sequencing, inform regulatory decisions, and ensure equitable access to evidence-based therapies.

https://doi.org/10.1016/j.jhepr.2026.101893
© 2026 APHP. Published by Elsevier B.V. on behalf of European Association for the Study of the Liver (EASL). This is an open access article under the CC BY license (http://creativecommons.org/licenses/by/4.0/).

Second-line TKI after first-line immunotherapy-based treatment in advanced HCC: Reconstructed IPD meta-analysis

Erman Akkus[1,2], Christian Hobeika[3,4], Julien Edeline[5], Clémence Hollande[6,7], Manon Allaire[8,9], Giuliana Amaddeo[10], Hélène Regnault[10], Marie Lequoy[11,12], Jean Charles Nault[13], Mohamed Bouattour[6,7,*]

JHEP Reports **2026**. vol. 8 | 1–13

Check for updates

Background: The efficacy of second-line tyrosine kinase inhibitors (TKIs) after first-line immunotherapy-based (IO) treatment in advanced hepatocellular carcinoma (HCC) is not well-established.

Methods: A systematic search was conducted to identify studies reporting outcomes with second-line TKIs after progression on first-line IO-based treatment. This reconstructed individual patient data (IPD) meta-analysis used survival data reconstructed from published Kaplan–Meier curves. Studies presenting or combining third-line-or-beyond data were excluded. Overall survival (OS) (primary endpoint) and progression-free survival (PFS) were analyzed using restricted mean survival time (RMST), and random-effects univariable and adjusted meta-regression analyses were performed to account for heterogeneity and potential confounding.

Results: A total of 1,663 patients (16 studies) were included (sorafenib [n = 769], lenvatinib [n = 691], regorafenib [n = 105], and cabozantinib [n = 98]). Most patients received atezolizumab-bevacizumab in the first line. Cabozantinib was excluded from primary analyses as 83.7% of the data were derived from a single study, and baseline characteristics data were limited. The regorafenib group had significantly more Child–Pugh A and less macrovascular invasion. Median OS of all patients was 9.8 months (95% CI 9.4–10.2). The 12-month OS was significantly longer with lenvatinib or regorafenib compared with sorafenib (ΔRMST, 1.49 months [95% CI 1.10–1.88], p <0.001 and 1.16 months [95% CI 0.41–1.96], p = 0.002). The median PFS for all patients was 3.2 months (95% CI 3.0–3.4). The 6-month PFS with lenvatinib or regorafenib was significantly longer than with sorafenib (ΔRMST, 1.28 months [95% CI 1.08–1.49], p <0.001, and 0.73 months [95% CI 0.38–1.09], p <0.001). Meta-regression analyses suggested similar results.

Conclusions: In this reconstructed IPD meta-analysis of predominantly retrospective studies, survival outcomes with second-line TKIs after IO-based therapy were heterogeneous. Lenvatinib and regorafenib showed consistent survival compared with sorafenib; however, findings are exploratory, limited by the observational nature of the data and residual confounding. Prospective studies are needed to define optimal post-immunotherapy sequencing strategies.

Systematic Review Registration: This study was registered with PROSPERO (Protocol No.: CRD420251133124).

© 2026 APHP. Published by Elsevier B.V. on behalf of European Association for the Study of the Liver (EASL). This is an open access article under the CC BY license (http://creativecommons.org/licenses/by/4.0/).

Introduction

Hepatocellular carcinoma (HCC) ranks as the sixth most prevalent malignancy and the third leading cause of cancer-related death globally, with a 5-year survival rate of only 21%, making it one of the most fatal gastrointestinal cancers.[1,2] Historically, sorafenib had been the standard first-line systemic treatment for advanced disease not amenable to locoregional treatments.[3] In 2018, the REFLECT trial established lenvatinib as a non-inferiority alternative to sorafenib.[4] Several second-line therapies were subsequently validated, including regorafenib, cabozantinib, and ramucirumab, but they were all studied in patients previously treated with sorafenib.[5–7]

Over the past 5 years, the advent of immunotherapy-based (IO-based) treatments has reshaped the treatment paradigm of advanced HCC. Currently, recommended first-line treatment options include IO plus AntiVEGF, IO plus tyrosine kinase inhibitor (TKI), and IO-IO combinations.[8] This includes atezolizumab-bevacizumab,[9] camrelizumab-ricoceranib,[10] durvalumab-tremelimumab,[11] and nivolumab-ipilimumab.[12] All were evaluated against sorafenib as a reference comparator, except nivolumab-ipilimumab, which was tested against both

* Corresponding author. Address: AP-HP, Hôpital Beaujon, Liver cancer and innovative therapy, Clichy, France, 100 BLD Du général Leclerc, 92110 Clichy, France, INSERM U1149, Centre de Recherche sur l'Inflammation (CRI), Paris, France. Tel.: +33 1 40 87 56 14.
E-mail address: mohamed.bouattour@aphp.fr (M. Bouattour).
https://doi.org/10.1016/j.jhepr.2026.101893

sorafenib and lenvatinib in the control arm,[8] Importantly, the pivotal second-line TKI trials that precede these IO-based regimens are lacking, leaving an unmet need regarding the efficacy of TKIs after IO. Small prospective and retrospective studies have begun to address this question; however, their limited sample sizes, heterogeneous study designs, and non-uniform comparators preclude drawing robust results and conclusions.

Therefore, we conducted a systematic review and meta-analysis of available studies, reconstructing individual patient-level survival from published Kaplan–Meier curves. Our objective was to evaluate the efficacy of second-line TKIs following first-line IO-based treatment in advanced HCC.

Materials and methods

Literature search

The systematic review was carried out following the Preferred Reporting Items for Systematic Reviews and Meta-Analyses (PRISMA) guidelines.[13] A literature search (completed by 26 August 2025) was performed across MEDLINE using the specified search strategy of terms (second line [Title/Abstract]) AND (hepatocellular carcinoma [Title/Abstract]) between 2020 to present. In the SCOPUS database, '(second line) AND (hepatocellular carcinoma)' was searched in 'title, abstracts, keywords' with the refinements of medicine, article, and English options from 2020 to present. In the Web of Science database, the same strategy was searched with the refinements of article and English options. European Society of Medical Oncology (ESMO) and American Society of Clinical Oncology (ASCO) meeting abstracts were searched using the specified strategy of terms '(second line) AND (hepatocellular carcinoma)' via the search tools of official websites.

Eligibility criteria for the studies

Original studies presenting survival data (a Kaplan–Meier curve for overall survival [OS]) of a second-line TKI after first-line IO-based treatment in advanced HCC were included. Randomized controlled trials, phase trials, retrospective, and prospective observational studies were eligible. Meeting abstracts/presentations were also considered. Exclusion criteria were reviews, case reports, editorials, correspondences, letters, *in vitro* studies, book chapters, books, notes, short surveys, study protocols, and studies for which the original documents were not accessible. Non-English language publications and duplicated studies were also excluded. Studies that did not present second-line treatment, studies in which the first-line treatments were not IO-based, if locoregional therapies or chemotherapy were implemented in the second line, studies mixing non-IO-based treatments in the first line, studies combining second-line TKIs as a group and not presenting individually, and studies not presenting OS with a Kaplan–Meier curve were excluded.

Data extraction

Titles and abstracts of the publications retrieved through the database search were screened according to inclusion-exclusion criteria, and full-texts of eligible studies were included (Fig. S1). Kaplan-Meier curves of OS and progression-free survival (PFS) of a specific TKI were obtained from published texts/posters/presentations to reconstruct individual patient data. For every TKI treatment arm, the following baseline characteristics were recorded if available: author and year of the study, region, design, sample size, median age, type of first-line IO-based treatment, numbers and percentages of patients with Eastern Cooperative Oncology Group Performance Score (ECOG-PS) 0, Child-Pugh class A, Barcelona Clinic Liver Cancer (BCLC) Stage C, Albumin-Bilirubin (ALBI) grade 1, macrovascular invasion (MVI), and extrahepatic spread, response data (based on RECIST 1.1), and adverse events observed in >5% of patients, if available.

Outcome

The primary and secondary endpoints were OS and PFS, respectively. The OS and PFS were calculated from the start date of the second-line TKI. Pooled OS and PFS with second-line TKIs after first-line IO-based treatment were presented and compared across TKIs. As the proportional hazard assumptions were violated, the endpoints were assessed by restricted mean survival times (RMSTs). Twelve-month OS and 6-month PFS were selected as primary and secondary endpoints based on individual study follow-up durations and median survival estimates. Baseline characteristics were compared across TKIs to interpret survival results in line with any prognostic factor differences. Objective response rates (ORRs), disease control rates (DCRs), and adverse events observed in >5% of patients were pooled and compared, if data were available. To address sources of heterogeneity among included studies, univariable and study-level adjusted meta-regression analyses were performed for OS and PFS, including available study characteristics.

Because 83.7% of the cabozantinib data were derived from a single study, baseline characteristics data were limited, and overall study quality was assessed as low; therefore, cabozantinib was not included in any primary analyses. Cabozantinib results are presented separately in the Supplementary material as exploratory data.

To address possible overlapping populations, an additional sensitivity analysis was performed for the primary endpoint of OS, excluding multicountry, continental, or global datasets, and restricting the analysis to studies reporting single-center or single-country cohorts.

Risk of bias assessment

As the review and meta-analyses included and analyzed studies based on a TKI arm(s) in each, the ROB-ME,[14] which is a tool for assessing the risk of bias as a result of missing evidence in a meta-analysis, was utilized to assess the risk of bias in treatment arms. The ROB-ME tool contains four steps. Step 1 is selecting and defining the meta-analyses that will be assessed. Step 2 is completing the study matrix for missing results. Step 3 is to consider the potential for missing studies across the systematic review. Step 4 is assessing the risk of bias attributable to missing evidence in the meta-analysis. Additionally, Modified Cowley's criteria scoring was utilized, which is a risk of bias scoring system for single-arm trials.[15] It included a total of 13 criteria, each of which was scored as follows: 2 for satisfactory reporting, 1 for partial reporting, and 0 for no reporting. The total score of 24–26/26 is grouped as

'low risk of bias', 20–23/26 as 'moderate risk of bias', and <20/26 as 'high risk of bias'.

Statistical analyses

Individual patient survival data were reconstructed from published Kaplan–Meier curves using the IPDfromKM package and application[16] (Fig. S2). Reconstructed data were pooled to obtain and compare survivals across TKIs. Pooled results were presented as Kaplan–Meier curves and analyzed by Cox regression. The calculated hazard ratios (HRs) were presented with 95% CI values. The proportional hazard assumption was tested by Schoenfeld residuals.[17] As the proportional hazard assumptions were violated and follow-up durations varied across studies, time-restricted survival comparisons (12-month OS and 6-month PFS) based on median follow-up durations and median survivals were performed by the RMST method and presented as absolute difference in months.[18] The method computed the area under the Kaplan–Meier curve of pooled reconstructed individual patient data. The meta-analyses of baseline characteristics, response, and toxicities were performed using the proportion and inverse variance methods and the Freeman–Tukey transformation, and presented with the random-effects model.[19] Univariable and adjusted random-effects meta-regression analyses were performed for time-specific RMST of OS and PFS (12-month OS and 6-month PFS) using restricted maximum likelihood estimation. Treatment group and study-level characteristics were included as moderators.[20] In the meta-regression, for each study, RMST and its standard error were estimated from reconstructed individual patient data and entered into a study-level dataset together with treatment group and published study characteristics. Continuous variables were presented as median in the study data and median (95% CI) for pooled analyses. Categorical variables were presented as the percentage in the study data and the percentage (95% CI) for pooled analyses. Higgins' I-squared statistics (I^2) was used to quantify the degree of heterogeneity between the studies. All p values were based on a two-tailed test of significance (p = 0.05). The analyses and visualization were performed using R Version 4.4.2 (RStudio Inc., Boston, MA, USA), and MedCalc Statistical Software version 22.026 (MedCalc Software Ltd, Ostend, Belgium).

Results

Studies and treatment arms

A total of 16 studies[21–38] met the inclusion criteria (Fig. S1, PRISMA diagram). One study was phase II, one was a prospective observational study, and the remaining were retrospective observational studies. From the 16 studies, 29 TKI arms as second-line treatment were identified (13 lenvatinib, 9 sorafenib, 3 regorafenib, and 4 cabozantinib), and data were extracted and analyzed. The most common first-line treatment was atezolizumab-bevacizumab (Table 1). In total, 1,565 patients were included from the studies for the primary endpoint of OS (sorafenib [n = 769, 49.1%], lenvatinib [n = 691, 44.2%], and regorafenib [n = 105, 6.7%]). Data for cabozantinib were limited (n = 98), with 83.7% (82/98) of patient data derived from a single study, which had a low-quality score. Therefore, cabozantinib was not included in the primary analyses and is presented separately in the Supplementary material as exploratory data. Thirteen of the studies also provided PFS data, and 1,279 patients were included in the secondary endpoint of PFS analyses (sorafenib [n = 604, 47.2%], lenvatinib [n = 570, 44.6%], and regorafenib [n = 105, 8.2%]).

Risk of bias assessment results

Step 1 of the ROB-ME tool for risk of bias assessment was defined in relevant sections of this study. Step 2 (result matrix for the risk of bias assessment) is presented in Table S1. Step 3 was concluded as 'We were likely to have found all eligible studies regardless of their results'. In Step 4, the 'Risk of bias judgment' was 'Low' for OS, 'Some concerns' for PFS and baseline characteristics, as some studies did not provide data for the analyses, and 'high' for response and toxicities, as most of the studies did not provide toxicity data, and the data reporting was heterogeneous among the ones that reported. According to the modified Cowley's criteria, six treatment arms had 'low' risk of bias, 20 arms 'moderate', and three arms 'high' risk of bias (Table S1).

Baseline characteristics of TKI groups

The baseline characteristics of TKI arms from the included studies are presented in Table 1. Pooled baseline characteristics between TKI groups were compared to assist in interpreting survival results. Because of the limited data, baseline characteristics of cabozantinib were not included in the analyses. No significant differences were observed across lenvatinib, sorafenib, and regorafenib for ECOG-0 (p = 0.125), BCLC stage C (p = 0.994), ALBI grade 1 (p = 0.702), or extrahepatic spread (p = 0.416) (Figs. S3a,c,d,f). However, the regorafenib group included a significantly higher rate of Child-Pugh class A patients (97% *vs.* 86% and 84%, p = 0.047) and fewer with MVI (22% *vs.* 38% and 44%, p = 0.012), suggesting a possible better prognostic status of patients in this group (Fig. S3b and e).

Overall survival

A total of 1,565 patients receiving one of lenvatinib, sorafenib, or regorafenib at second line after progressing with a first-line IO-based treatment were analyzed for OS. The median OS for all patients was 9.8 months (95% CI 9.4–10.2) (Fig. 1). By agent, median OS was 11.9 months (95% CI 10.5–12.9) with lenvatinib, 7.9 months (95% CI 7.1–8.6) with sorafenib, and 10.4 months (95% CI 9.6–15.9) with regorafenib (Fig. 2A). The cross-TKI comparisons for OS with HRs were presented in Fig. 2A. Lenvatinib showed significantly better OS when compared with sorafenib (HR: 0.68, 95% CI 0.61-0.77). However, the proportional hazard assumption was violated by the Schoenfeld residual test (p <000.1) (Fig. S4).

As the proportional hazard assumption was violated, all studies had at least a 12-month follow-up for OS, the pooled median OS of all patients was 9.9 months, and the regorafenib arm had a maximum survival of 20 months; RMST for the 12th and 20th months were analyzed. 12-month OS was significantly longer with lenvatinib and regorafenib compared with sorafenib (ΔRMST, 1.49 months [95% CI 1.10–1.88], p <0.001 and 1.16 months [95% CI 0.41–1.96], p = 0.002, respectively). There was no difference between lenvatinib and regorafenib

Table 1. Characteristics of lenvatinib, sorafenib, regorafenib, and cabozantinib arms in included studies.

Author, year	Region	Design	Sample size	Age (med.)	Male, n, (%)	ECOG-PS 0, n (%)	1L treatment	CP-A n, (%)	BCLC-C, n (%)	ALBI, grade 1, n, (%)	MVI, n, (%)	Extrahepatic, n, (%)
**Lenva**												
**Chen *et al.*, 2022**[21]	Taiwan	Ret.	9	66	8 (89)	NA	Atezo-Beva	6 (67)	NA	1 (11)	9 (100)	5 (56)
Chon *et al.*, 2023[22]	Korea	Ret.	40	60	36 (90)	26 (65)	Atezo-Beva	37 (92.5)	36 (90)	NA	17 (52.1)	29 (72.5)
Decreacker *et al.*, 2025[23]	France	Ret.	35	NA	NA	NA	Atezo-Beva	NA	NA	NA	NA	NA
Hiraoka,2023[24]	Japan	Ret.	101	72	77 (76.2)	76 (75.2)	Atezo-Beva	82 (81.2)	65 (64.4)	24 (23.8)	30 (29.7)	45 (45.6)
Lee *et al.*, 2025[25]	Asia-Pacific	Ret.	154	61	128 (83.1)	77 (50)	Atezo-Beva	135 (87.6)	126 (81.1)	NA	42 (27.3)	106 (68.8)
Lombardi *et al.*, 2025[26,37]	Global	Pros.	125	61	103 (82.4)	69 (55.2)	Atezo-Beva	NA	56 (44.8)	92 (73.6)	NA	60 (48)
Muto *et al.*, 2023[27]	Japan	Ret.	20	70	17 (85)	12 (60)	Atezo-Beva	16 (80)	14 (70)	6 (30)	NA	9 (45)
Persano *et al.*, 2024[28]	Global	Ret.	86	NA	67 (77.9)	NA	Atezo-Beva	85 (98.8)	53 (61.6)	82 (95.3)	NA	NA
Falette-Puisieux *et al.*, 2023[29]	France	Ret.	8	65	7 (87.5)	0 (0)	Atezo-Beva	3 (37.5)	6 (75)	1 (12.5)	2 (25)	4 (50)
Qin *et al.*, 2022[30]	China	Ret.	20	NA	14 (70)	NA	Atezo-Beva (n = 7)<br>Pembro + Sora, (n = 3)<br>Camre + Apatinib (n = 10)	12 (60)	20 (100)	NA	NA	11 (55)
Yano *et al.*, 2023[31]	Japan	Ret.	24	NA	20 (83.3)	19 (79.2)	Atezo-Beva	16 (68)	16 (68)	NA	8 (33.3)	11 (45.8)
Yoo *et al.*, 2024[32,38]	Korea	Phase II	50	66	42 (82)	9 (18)	Atezo-Beva	50 (100)	38 (76)	NA	12 (24)	NA
Yoo *et al.*, 2021[33]	Korea, Hong-Kong, Singapore	Ret.	19	65	10 (52.6)	6 (31.6)	Atezo-Beva	19 (100)	19 (100)	NA	9 (47.4)	NA
**Sora**												
Chen *et al.*, 2022[21]	Taiwan	Ret.	19	68	18 (95)	NA	Atezo-Beva	15 (79)	NA	5 (26)	14 (74)	13 (68)
Chon *et al.*, 2023[22]	Korea	Ret.	86	63	75 (87.2)	43 (50)	Atezo-Beva	54 (62.8)	73 (84.9)	NA	41 (47.7)	58 (67.4)
Decreacker *et al.*, 2025[23]	France	Ret.	78	NA	NA	NA	Atezo-Beva	NA	NA	NA	NA	NA
Lee, 2025[25]	Asia-Pacific	Ret.	324	61	264 (81.5)	124 (38.3)	Atezo-Beva	235 (72.6)	246 (76.8)	NA	90 (27.8)	193 (59.6)
Lombardi *et al.*, 2025[26]	Global	Pros.	105	61	83 (79.8)	33 (31.4)	Atezo-Beva	NA	71 (67.6)	49 (47.6)	NA	47 (44.8)
Möhring *et al.*, 2025[34]	Europe	Ret.	36	NA	NA	NA	Atezo-Beva	NA	NA	NA	NA	NA
Persano *et al.*, 2024[28]	Global	Ret.	51	NA	44 (86.3)	NA	Atezo-Beva	48 (94.1)	34 (66.7)	49 (96.1)	NA	NA
Falette-Puisieux *et al.*, 2023[29]	France	Ret.	41	62	32 (78.1)	5 (12.2)	Atezo-Beva	34 (82.9)	39 (95.1)	6 (14.6)	20 (48.8)	34 (82.9)
Yoo *et al.*, 2021[33]	Korea, Hong-Kong, Singapore	Ret.	29	59	25 (86.2)	5 (17.2)	Atezo-Beva	29 (100)	19 (100)	NA	9 (31)	NA
**Regora**												
Cheon *et al.*, 2025[35]	Korea	Ret.	40	56	31 (77.5)	24 (60)	Atezo-Beva	40 (100)	39 (97.5)	17 (42.5)	10 (25)	34 (85)
Lee *et al.*, 2025[25]	Asia-Pacific	Ret.	36	56	28 (77.8)	15 (41.7)	Atezo-Beva	34 (94.5)	12 (33.3)	NA	5 (13.9)	11 (30.6)
Falette-Puisieux *et al.*, 2023[29]	France	Ret.	29	63	23 (79.3)	1 (3.4)	Atezo-Beva	27 (93.1)	27 (93.1)	6 (20.7)	8 (27.6)	25 (86.2)
**Cabo**												
Ahn *et al.*, 2025[36]	USA	Ret.	28	68	22 (78.6)	NA	Nivo (n = 20)<br>Pembro (n = 4)<br>Atezo (n = 2)<br>Nivo+ Ipi (n = 2)	NA	NA	NA	NA	NA
Ahn *et al.*, 2025[36]	USA	Ret.	54	63	45 (83.3)	NA	Atezo + Beva (n = 50)<br>Nivo + Sora (n = 2)<br>Nivo + Lenva (n = 2)	NA	NA	NA	NA	NA
Lee *et al.*, 2025[25]	Asia-Pacific	Ret.	12	61	11 (91.7)	2 (16.7)	Atezo-Beva	11 (91.6)	9 (75)	NA	2 (16.7)	8 (66.7)
Falette-Puisieux *et al.*, 2023[29]	France	Ret.	4	46	3 (75)	0 (0)	Atezo-Beva	4 (100)	4 (100)	2 (50)	1 (25)	3 (75)

1L, first-line; Atezo, atezolizumab; Atezo-Beva, atezolizumab-bevacizumab; BCLC: Barcelona Clinic Liver Cancer, Cabo, cabozantinib; Camre, camrelizumab; CP-A, Child–Pugh class A; ECOG-PS, European Cooperation Oncology Group performance score; Lenva, lenvatinib; Med, median; MVI, macrovascular invasion; NA, not available; Nivo, nivolumab; Nivo-Ipi, nivolumab-ipilimumab; Nivo-Lenva, nivolumab-lenvatinib; Nivo-Sora, nivolumab-sorafenib; Pembro, pembrolizumab; Pembro-Sora, pembrolizumab-sorafenib; Pros, prospective; Regora, regorafenib; Ret, retrospective; Sora, Sorafenib.

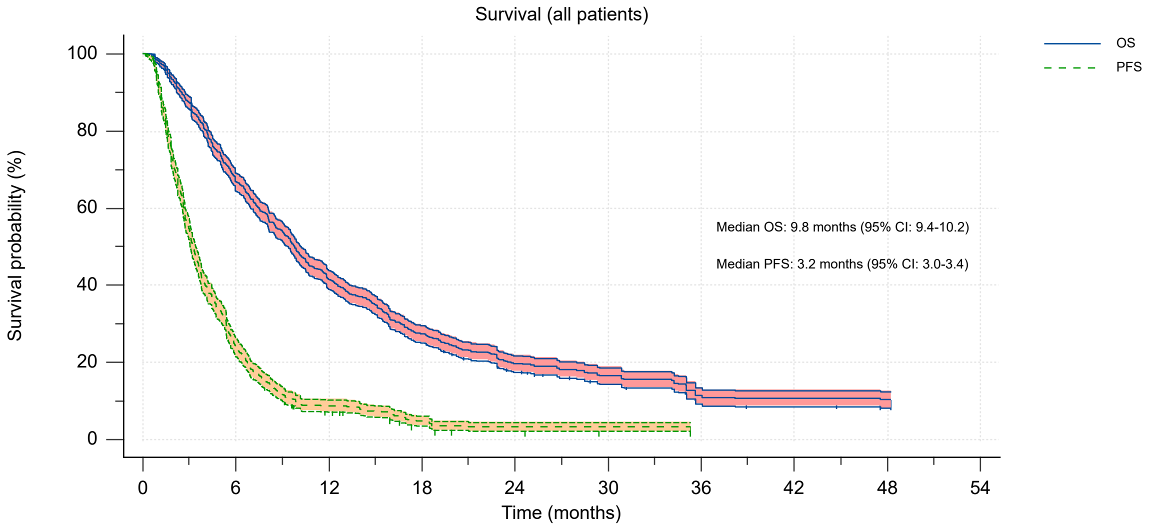

N° at risk										
Group: OS	1,565	1,046	615	393	190	121	61	44	22	0
Group: PFS	1,279	306	96	43	26	4	0	0	0	0

Fig. 1. Pooled OS and PFS with second-line TKIs (lenvatinib, sorafenib, and regorafenib) in advanced HCC. Reconstructed individual patient data were pooled and presented as Kaplan–Meier curves. HCC, hepatocellular carcinoma; OS, overall survival; PFS, progression-free survival; TKIs, tyrosine kinase inhibitors.

(ΔRMST, 0.32 months [95% CI -0.42 to 1.07], *p* = 0.393). The RMST of 20-month OS showed a similar pattern (Fig. 2B).

OS analyses that included cabozantinib data were provided in Figs. S5 and S6.

A sensitivity analysis for the primary endpoint of OS was performed for the risk of duplicate patients from multicountry studies and national studies. OS analyses were repeated after excluding multicountry studies and showed similar results (Fig. S9).

Progression-free survival

A total of 1,279 patients receiving one of lenvatinib, sorafenib, or regorafenib at second line after progressing with a first-line IO-based treatment were analyzed for PFS. The median PFS overall was 3.2 months (95% CI 3.0–3.4) (Fig. 1). Median PFS with lenvatinib, sorafenib, and regorafenib was 4.5 months (95% CI 4.0–5.1), 2.3 months (95% CI 2.2–2.5), and 3.4 months (95% CI 2.7–3.9), respectively (Fig. 3A). The cross-TKI comparisons for PFS with HRs were presented in Fig. 3A. Lenvatinib showed significantly longer PFS when compared with sorafenib (HR: 0.51, 95% CI 0.45–0.58). However, the proportional hazard assumption was violated by the Schoenfeld residual test (*p* <000.1) (Fig. S4).

As the proportional hazard assumption was violated and all studies had at least a 6-month follow-up for PFS, RMSTs for the 6th and 9th months were analyzed for PFS. The 6-month PFS with lenvatinib was significantly longer than with lenvatinib and regorafenib than sorafenib (ΔRMST, 1.28 months, 95% CI 1.08–1.49), *p* <0.001, and 0.73 months, 95% CI 0.38–1.09, *p* <0.001), and was longer with lenvatinib than with regorafenib (ΔRMST, 0.54 months, 95% CI 0.19–0.90, *p* = 0.002). The RMST of 9-month PFS showed a similar pattern (Fig. 3B). PFS analyses that included cabozantinib data were provided in Figs. S7 and S8.

Meta-regression analyses

Meta-regression analyses were performed to explore sources of heterogeneity and adjust for potential confounders for the primary (12-month RMST of OS) and secondary (6-month RMST of PFS) endpoints. In univariable analyses, treatment (sorafenib *vs.* lenvatinib), ECOG-0, ALBI-1, and study quality scores were identified as moderators of OS (Table 2). For PFS, only treatment (sorafenib *vs.* lenvatinib) and male sex were the moderators (Table 2). In study-level adjusted meta-regression models, the treatment effect remained associated with differences in RMST estimates after accounting for other individual covariants. Sorafenib showed a shorter OS and PFS pattern than lenvatinib, and regorafenib showed a similar OS and PFS pattern compared to lenvatinib in most of the analyses, adjusting for covariants (Fig. 4A,B).

Response

The response data of the TKI arms based on RECIST 1.1 are presented in Table 3. Not enough response data were available for cabozantinib. Therefore, ORR and DCR were pooled and compared among other TKIs. Sorafenib showed significantly lower ORR and DCR. The ORR with lenvatinib, regorafenib, and sorafenib was 14% (95% CI 8–21), 9% (95% CI 4–19), and 3% (95% CI 1–5), respectively (*p* <0.001) (Fig. 5A). The DCR with lenvatinib, regorafenib, and sorafenib was 70% (95% CI 56–82), 80% (95% CI 70–89), and 41% (95% CI 28–55), respectively (*p* <0.001) (Fig. 5B).

Adverse events

Safety reporting was inconsistent across studies. Less than half of the TKI arms (12/29) provided usable adverse event data, and definitions of toxicity (any grade *vs.* grade ≥3; Common Terminology Criteria for Adverse Events (CTCAE) version) were not

A

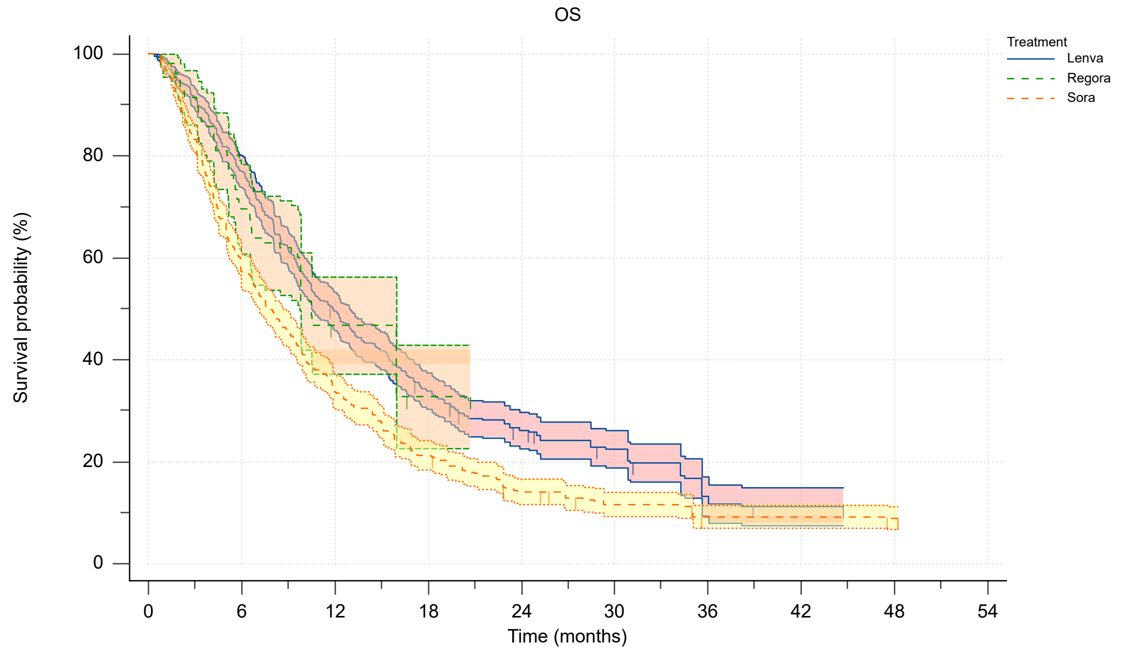

N° at risk										
Group: Lenva	691	532	326	221	95	58	26	9	0	0
Group: Regora	105	73	30	9	0	0	0	0	0	0
Group: Sora	769	441	259	163	95	63	35	35	22	0

Treatment	Median OS (month)	95% CI	Comparison	HR	95% CI
Lenva	11.9	10.5-12.9	**Lenva *vs.* Sora**	**0.68**	**0.61-0.77**
Regora	10.4	9.6-15.9	**Regora *vs.* Sora**	**0.69**	**0.54-0.89**
Sora	7.9	7.1-8.6	**Lenva *vs.* Regora**	0.98	0.77-1.25

B

Treatment	RMST 12 month OS (month difference, 95% CI)	*p*	RMST 20 month OS (month difference, 95% CI)	*p*
Lenva *vs.* Sora	**1.49 (1.10-1.88)**	**<0.001**	**2.58 (1.90-3.26)**	**<0.001**
Regora *vs.* Sora	**1.16 (0.41-1.96)**	**0.002**	**2.30 (0.90-3.70)**	**0.001**
Lenva *vs.* Regora	0.32 (-0.42-1.07)	0.393	0.27 (-1.12-1.68)	0.697

Fig. 2. Comparison of OS. (A) Comparison of OS across TKIs in the second-line treatment of advanced HCC, (B) RMST analyses for 12-month and 20-month OS. Reconstructed individual patient data were pooled and compared by Cox regression to obtain HRs and by RMST to obtain month differences at prespecified time points. 12-month OS by RMST was the primary endpoint of the study. Significance level is p <0.05. Values written in bold represent statistical significance. HR, hazard ratio; Lenva, lenvatinib; OS, overall survival; Regora, regorafenib; RMST, restricted mean survival time; Sora, sorafenib; TKIs, tyrosine kinase inhibitors.

uniform (Table S2). Among TKIs with sufficient data (lenvatinib, sorafenib, and regorafenib), the most common adverse events were hand-foot syndrome, fatigue, hypertension, and diarrhea, and were amenable to an analysis by pooling frequencies of any grade event reported. Pooled and comparative analyses are presented in Fig. S10. These findings should be interpreted with caution and considered as descriptive only, as adverse event data were limited by incomplete and heterogeneous adverse event reporting across studies.

Discussion

This meta-analysis evaluated TKIs in the second-line setting after first-line IO-based treatment, mainly atezolizumab-bevacizumab, in advanced HCC. Our findings suggest that different TKIs have heterogeneous survival outcomes in this setting, with a potential advantage of lenvatinib or regorafenib compared with sorafenib in the exploratory analyses.

Historically, TKIs such as lenvatinib, regorafenib, and cabozantinib were validated in the population of patients previously treated with sorafenib. In the first-line REFLECT trial, median PFS with lenvatinib and sorafenib was 7.4 *vs.* 3.7 months (p <0.001), favoring lenvatinib. Median OS was similar (13.6 *vs.* 12.3 months).[4] Although there is no robust prospective clinical trial data for lenvatinib after sorafenib, a 5.9-month OS was reported.[39] Regorafenib after sorafenib provided a median PFS of 3.1 months and OS of 10.6 months in the RESORCE trial.[5] The median PFS and OS with cabozantinib after sorafenib were 5.2 and 10.2 months in the CELESTIAL trial.[7] The shift toward IO-based treatments in first line creates an evidence gap, as the efficacy of these TKIs post-IO has not been systematically evaluated in prospective cohorts. Biologically, those treatments have a potential immunomodulatory

A

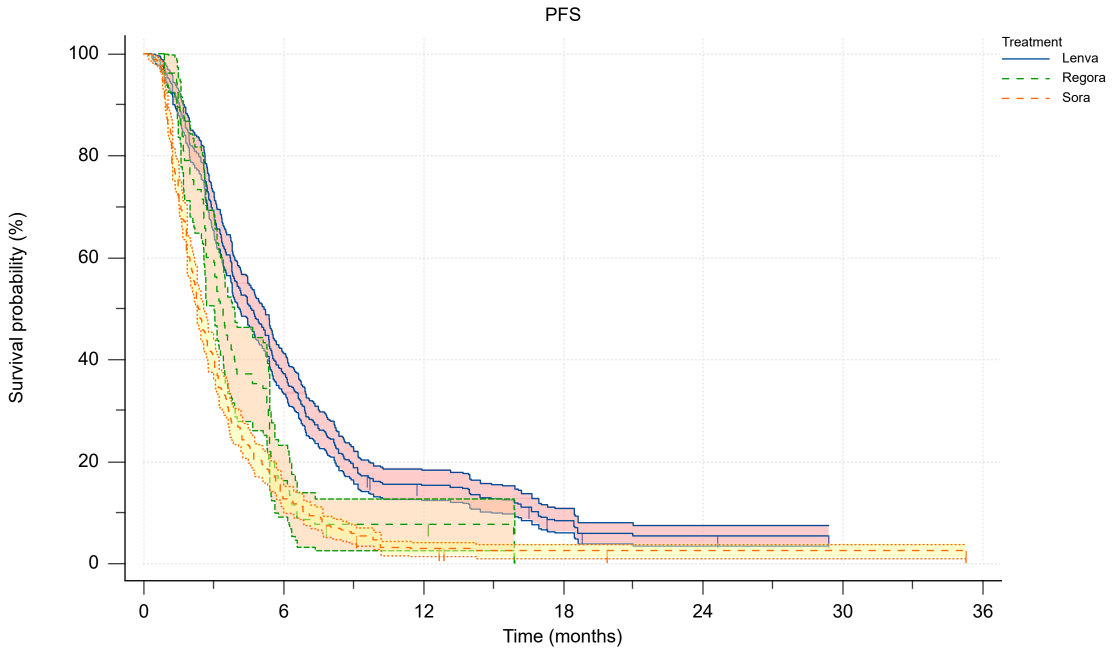

N° at risk							
Group: Lenva	570	213	75	37	22	0	0
Group: Regora	105	17	6	0	0	0	0
Group: Sora	604	76	15	6	4	4	0

Treatment	Median PFS (month)	95% CI	Comparison	HR	95% CI
Lenva	4.5	4.0-5.1	**Lenva *vs.* Sora**	**0.51**	**0.45-0.58**
Regora	3.4	2.7-3.9	**Regora *vs.* Sora**	**0.76**	**0.61-0.96**
Sora	2.3	2.2-2.5	**Lenva *vs.* Regora**	**0.66**	**0.53-0.83**

B

Treatment	RMST 6 month PFS (month difference, 95% CI)	*p*	RMST 9 month PFS (month difference, 95% CI)	*p*
Lenva *vs.* Sora	**1.28 (1.08-1.49)**	**<0.001**	**1.84 (1.54-2.13)**	**<0.001**
Regora *vs.* Sora	**0.73 (0.38-1.09)**	**<0.001**	**0.74 (0.28-1.19)**	**0.001**
Lenva *vs.* Regora	**0.54 (0.19-0.90)**	**0.002**	**1.10 (0.62-1.57)**	**<0.001**

Fig. 3. Comparison of PFS. (A) Comparison of PFS across TKIs in the second-line treatment of advanced HCC, (B) RMST analyses for 6-month and 9-month PFS. Reconstructed individual patient data were pooled and compared by Cox regression to obtain HRs and by RMST to obtain month differences at prespecified time points. 6-month PFS by RMST was the secondary endpoint of the study. Significance level is p <0.05. Values written in bold represent statistical significance. HR, hazard ratio; Lenva, Lenvatinib; PFS, progression-free survival; Regora, regorafenib; RMST, restricted mean survival time; Sora, sorafenib; TKIs, tyrosine kinase inhibitors.

effect on the tumor microenvironment.[40] Therefore, the efficacy of TKIs after IO-based treatment may differ from previously validated settings. For example, in our analysis, lenvatinib showed 11.9 months of OS at second line after IO-based first-line, which seems to be longer than after first-line sorafenib. However, PFS and OS in our analyses with regorafenib after IO-based treatments seemed to be similar to those in the RESORCE trial.

As the proportional hazard assumptions were violated, we used restricted RMST for our pooled analyses. Unlike HRs, RMST does not rely on the proportional hazards assumption and provides an absolute, time-based measure of treatment effect that remains valid in the presence of crossing or time-varying hazards. RMST can be understood as the average survival time gained within a clinically meaningful and prespecified time horizon. This interpretation aligns closely with clinical decision-making, as it expresses benefit in absolute time units rather than relative risk reductions. Based on follow-up durations in the included studies, RMST of 12-month OS and 6-month PFS were focused as endpoints.

In our pooled analyses, lenvatinib showed an average of 1.49 months of OS gain in the first year compared with sorafenib. Regorafenib also showed a 1.16-month gain, but there was no difference between lenvatinib and regorafenib. Moreover, lenvatinib showed 6-month PFS gain compared with both sorafenib and regorafenib, and regorafenib showed a gain compared to sorafenib. These findings should be interpreted with caution, as the regorafenib cohorts have baseline imbalances with more favorable prognostic characteristics, such as a higher proportion of Child–Pugh class A and lower MVI.

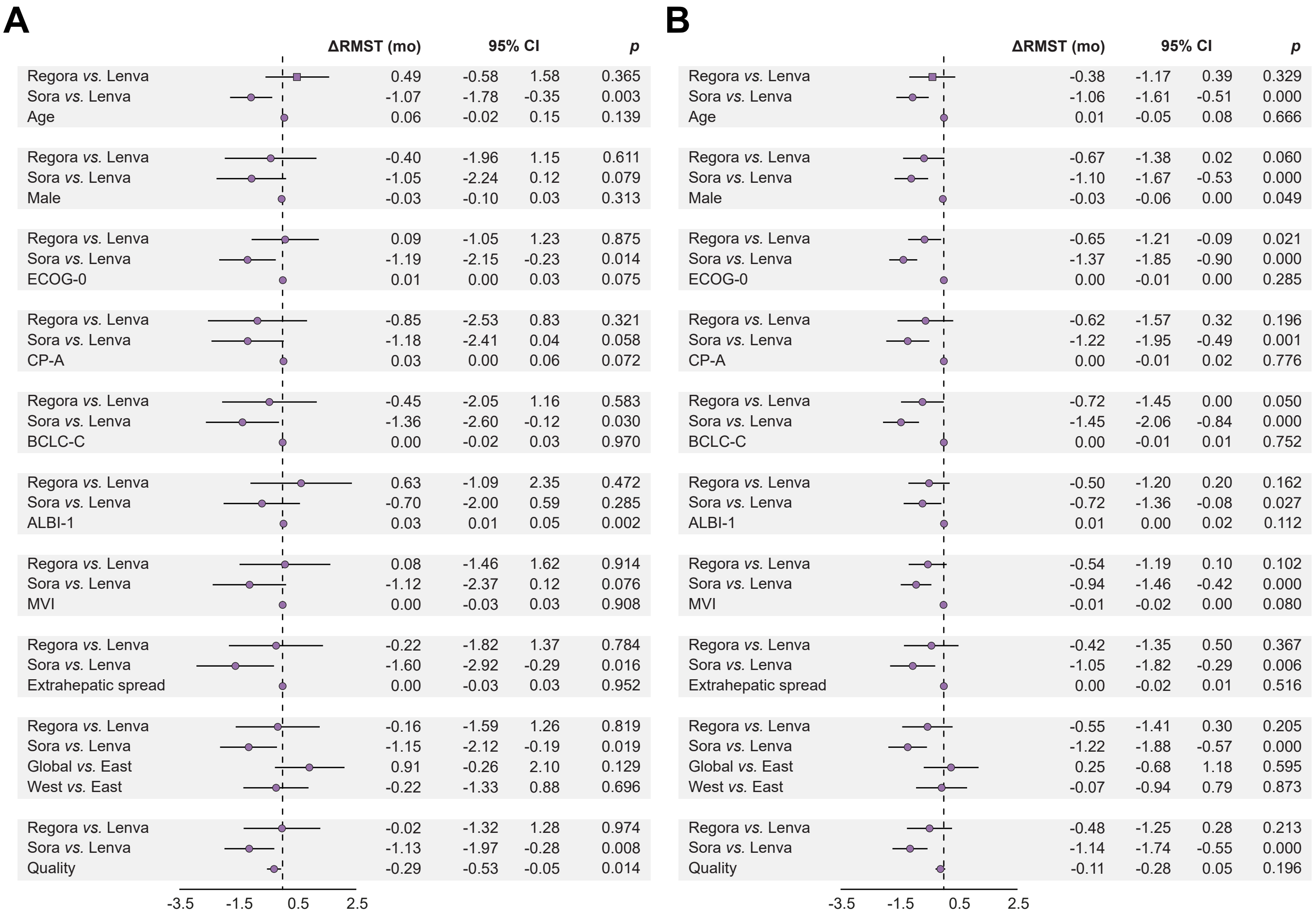

Fig. 4. Random-effects meta-regression of treatment effect adjusted by trial-level variables. (A) 12-month OS RMST and (B) 6-month PFS RMST. Analyses were performed using restricted maximum likelihood estimation. Treatment group and study-level characteristics were included as moderators. For each study, RMST and its standard error were estimated from reconstructed individual patient data and entered into a study-level dataset together with treatment group and published study characteristics. Significance level was $p < 0.05$. The variables except treatment and region were continuous variables. BCLC, Barcelona Clinic Liver Cancer; cont, continuous; CP-A, Child-Pugh class A; ECOG-PS, European Cooperation Oncology Group performance score; Lenva, lenvatinib; mo, month; MVI, macrovascular invasion; OS, overall survival; PFS, progression-free survival; RMST, restrictive-mean survival time; Regora, regorafenib; Sora, sorafenib.

Although we were not able to perform analyses with real individual patient-level data, these baseline imbalances should be considered when interpreting the survival results. Additionally, trial-level meta-regression analyses supported these pooled analyses, showing similar tendencies.

Although pooled baseline characteristics analyses and meta-regression analyses were performed, the heterogeneity and residual confounding cannot be eliminated because of the absence of true individual patient-level data. For example, the studies did not report the HCC etiology uniformly, precluding the proper analysis. However, the geographical region analysis, showing no association, may partly cover the potential etiological differences.

Our literature search revealed limited data on cabozantinib, with 83.7% of the data derived from a single study. This study showed strikingly unexpected prolonged survival (Ahn *et al.*).[36] However, this study used US claims data; baseline characteristics were not provided, and included non-standard first-line IO-based regimens. Therefore, cabozantinib was not included in the analyses. However, another phase II trial by Chan *et al.*[41] investigated cabozantinib in patients who received previous IO-based treatments. However, 42.6% of the patients received two prior lines of treatment in this study, including TKIs. A total of 23.4% of patients received IO in the second line. Median PFS and OS with cabozantinib were 4.3 months and 14.3 months, respectively. Thus, while post-IO cabozantinib remains a promising option, further prospective evaluation and more data that includes patients who received only one previous line of treatment is warranted.

Indeed, the LEAP-002 trial investigated pembrolizumab plus lenvatinib *vs*. lenvatinib in the first-line treatment of advanced HCC. Although the trial was negative and did not show a difference between arms, the lenvatinib arm showed a PFS of 8 months and an OS of 19 months,[42] which was better than the REFLECT trial. Similarly, in the control arm of the first-line CheckMate 9DW study, 85% of patients received lenvatinib, and median OS was 20.6 months.[12] Therefore, consistent with these results, our second-line analyses suggest a prominent activity of lenvatinib in advanced HCC. Moreover, a previously published simulation model based on data from phase

III randomized clinical trials to identify optimal risk–benefit sequential strategies in advanced HCC suggested that the sequence atezolizumab-bevacizumab followed by lenvatinib may be associated with a favorable outcome.[43]

Toxicity profiles are core determinants in treatment preferences, especially in previously treated patients. As the majority of the data in our analyses comes from retrospective studies, the toxicity data were very limited and inconclusive. The available data suggested the expected toxicity profiles of TKIs. Yet, analyses were performed with amenable data and suggested that lenvatinib is associated with less hand-foot syndrome but more diarrhea and hypertension in this setting. Proteinuria is also a concerning toxicity of lenvatinib. However, these toxicities were mainly grade 1 or 2, therefore manageable. Overall, toxicity analyses in our study should be assessed as descriptive rather than comparative, given the incomplete and heterogeneous reporting of adverse events across retrospective studies.

Beyond efficacy and safety profiles of TKIs after first-line IO-based therapy, treatment preference is also influenced by regulatory approval and real-world accessibility of TKIs. In most of the countries, such as Italy, Türkiye, and other European countries, sorafenib remains the only TKI approved in this setting, and other TKIs are frequently used off-label. These regulatory and reimbursement constraints may limit the applicability of emerging real-world evidence and stress the need for prospective data and regulatory reassessment to better align approved treatment options with evolving clinical practice.

Our study has several strengths. The treatment algorithm of advanced HCC rapidly evolved, and there is no prospective study comparing different TKIs in the second line after IO-based treatment. Moreover, over 1,600 patients were included for OS analyses and nearly 1,300 for PFS, the largest dataset available on this specific question. Our study provided an insight to guide practice, analyzing reconstructed individual patient data.

This study also has several limitations. First, this study was an aggregate-data meta-analysis based on reconstructed individual patient data, which is informative but not equivalent to a true individual data meta-analysis. Most of the included studies were retrospective, limiting the reliability of the data. Because the analysis relies on reconstructed data from largely retrospective cohort studies, it inherently lacks the ability to adjust for patient-level covariates. Second, there was missing data for the secondary endpoint PFS, baseline characteristics, and adverse events. The data for locoregional therapies before first-line treatment, and third-line or beyond treatment, TKI treatment duration, and dose reduction data were not available. Not all studies provided data for baseline characteristics, and there was considerable heterogeneity between studies. Although comparison of baseline characteristics of the groups and meta-regression analyses were informative, this cannot replace patient-level adjusted analyses, which are not possible in our study. Individual patient data from the original studies would provide adjusted analyses. Although network meta-analyses (NMA) could theoretically be considered, a valid anchored NMA was not feasible, since an anchored NMA requires randomization within trials. Pseudo-anchored or population-adjusted indirect comparison methods (*e.g.* Matching-Adjusted Indirect Comparison (MAIC)) would be

Table 2. Random-effects univariable meta-regression of RMST.

	12-month OS					6-month PFS				
Moderator	Number of arms	RMST difference (months)	95% CI	*p* value	I^2 (%)	Number of arms	RMST difference (months)	95% CI	*p* value	I^2 (%)
Treatment										
Regora *vs.* Lenva	25	-0.36	-1.80 to 1.08	0.621	86.1	20	-0.60	-1.38 to 0.16	0.124	88.3
Sora *vs.* Lenva		-1.13	-2.09 to -0.16	**0.021**			-1.22	-1.83 to -0.61	**<0.001**	
Median age (cont. variable)	18	0.04	-0.05 to 0.15	0.363	77.9	18	0.04	-0.03 to 0.12	0.264	91.7
Male % (cont. variable)	22	-0.05	-0.11 to 0.01	0.123	89.9	20	-0.04	-0.08 to 0.00	**0.037**	93
ECOG 0 % (cont variable)	17	0.02	0.00 to 0.04	**0.020**	81.7	17	0.00	-0.00 to 0.02	0.432	92.2
CP-A % (cont variable)	20	0.02	-0.00 to 0.06	0.105	89.9	18	0.00	-0.02 to 0.02	0.907	94.2
BCLC-C % (cont variable)	20	-0.00	-0.03 to 0.02	0.805	91.7	18	-0.00	-0.02 to 0.01	0.734	94.8
ALBI-G1 % (cont variable)	12	0.02	0.00 to 0.04	**0.005**	81.7	10	0.01	-0.00 to 0.03	0.115	80.3
MVI % (cont. variable)	16	-0.01	-0.04 to 0.02	0.431	85.2	16	-0.01	-0.03 to 0.00	0.084	89.2
Extrahepatic % (cont variable)	17	-0.00	-0.04 to 0.03	0.693	90.4	17	-0.01	-0.03 to 0.00	0.220	94.4
Region										
Global *vs.* East	25	0.68	-0.61 to 1.97	0.302	88.1	25	0.06	-1.17 to 1.29	0.922	94.4
West *vs.* East		-0.52	-1.70 to 0.66	0.391			-0.30	-1.39 to 0.78	0.583	
Quality score (cont. variable)	25	-0.27	-0.54 to 0.01	**0.041**	86.4	25	-0.16	-0.38 to 0.05	0.133	93.4

Analyses were performed using restricted maximum likelihood estimation. Treatment group and study-level characteristics were included as moderators. For each study, RMST and its standard error were estimated from reconstructed individual patient data and entered into a study-level dataset together with treatment group or published study characteristics. Significance level was $p < 0.05$. Values written in bold represent statistical significance. BCLC, Barcelona Clinic Liver Cancer; Cont, continuous; CP-A, Child–Pugh class A; ECOG-PS, European Cooperation Oncology Group performance score; Lenva, lenvatinib; MVI, macrovascular invasion; OS, overall survival; PFS, progression-free survival; Regora, regorafenib; RMST, restrictive-mean survival time; Sora, sorafenib.

Table 3. Efficacy data with tyrosine kinase inhibitors at second-line after first-line immunotherapy-based treatment in the included studies.

Study	Treatment	Sample size	ORR, n (%)	CR, n (%)	PR, n (%)	SD, n (%)	DCR, n (%)	PD, n (%)	Median PFS, mo	Median OS, mo
Chen *et al.*, 2022[21]	Lenva	9	1 (11)	NA	NA	NA	2 (22.2)	NA	2	3.8
Chon *et al.*, 2023[22]	Lenva	40	3 (7.5)	0 (0)	3 (7.5)	24 (60)	27 (67.5)	11 (27.5)	3.5	10.3
Decraecker *et al.*, 2025[23]	Lenva	35	NA	NA	NA	NA	NA	NA	NA	14
Hiraoka, 2023[24]	Lenva	101	11 (10.9)	1 (1)	10 (9.9)	36 (35.6)	47 (46.5)	24 (23.8)	4.4	15.7
Lee *et al.*, 2025[25]	Lenva	154	9 (5.9)	0 (0)	9 (5.9)	77 (50.3)	86 (56.2)	45 (29.4)	4	8
Lombardi *et al.*, 2025[26,37]	Lenva	125	NA	NA	NA	NA	NA	NA	5.5	11.9
Muto *et al.*, 2023[27]	Lenva	20	5 (25)	0	5 (25)	14 (70)	19 (95)	0 (0)	6	10.5
Persano *et al.*, 2024[28]	Lenva	86	NA	NA	NA	NA	NA	NA	NA	18.9
Falette-Puisieux *et al.*, 2023[29]	Lenva	8	NA	NA	NA	NA	NA	NA	4.4	5.7
Qin *et al.*, 2022[30]	Lenva	20	6 (30)	NA	NA	13 (65)	19 (95)	1 (5)	7.5	12.6
Yano *et al.*, 2023[31]	Lenva	24	8 (33.3)	0 (0)	8 (33.3)	10 (41.7)	18 (75)	5 (20.8)	4	15.3
Yoo *et al.*, 2024[32,38]	Lenva	50	6 (12)	0 (0)	6 (12)	36 (72)	42 (84)	6 (12)	5.4	8.6
Yoo *et al.*, 2021[33]	Lenva	19	3 (15.8)	0 (0)	3 (15.8)	9 (47.4)	12 (63.2)	6 (31.6)	6.1	16.6
Chen *et al.*, 2022[21]	Sora	19	0 (0)	NA	NA	NA	9 (47.4)	NA	2.6	8.3
Chon *et al.*, 2023[22]	Sora	86	5 (5.8)	0 (0)	5 (5.8)	16 (18.6)	21 (24.4)	47 (54.7)	1.8	5.6
Decreacker *et al.*, 2025[23]	Sora	78	NA	NA	NA	NA	NA	NA	NA	13.2
Lee *et al.*, 2025[25]	Sora	324	12 (3.7)	0 (0)	12 (3.7)	114 (35.2)	126 (38.9)	140 (43.2)	2.3	6.3
Lombardi *et al.*, 2025[26,37]	Sora	105	NA	NA	NA	NA	NA	NA	2.6	7.4
Möhring *et al.* 2025[34]	Sora	36	NA	NA	NA	NA	NA	NA	NA	7.1
Persano *et al.*, 2024[28]	Sora	51	NA	NA	NA	NA	NA	NA	NA	14.3
Falette-Puisieux *et al.*, 2023[29]	Sora	41	NA	NA	NA	NA	NA	NA	2.6	7
Yoo *et al.*, 2021[33]	Sora	29	0 (0)	0 (0)	0 (0)	18 (62)	18 (62)	8 (27.6)	2.5	11.2
Cheon *et al.*, 2025[35]	Regora	40	4 (10)	0 (0)	4 (10)	29 (72.5)	33 (82.5)	7 (17.5)	3.5	10.5
Lee *et al.*, 2025[35]	Regora	36	4 (10.1)	0 (0)	4 (10.1)	24 (66.7)	28 (77.8)	7 (19.4)	3.6	9.7
Falette-Puisieux *et al.*, 2023[29]	Regora	29	NA	NA	NA	NA	NA	NA	2.6	15.8
Ahn *et al.*, 2025[36]	Cabo	28	NA	NA	NA	NA	NA	NA	NA	37
Ahn *et al.*, 2025[36]	Cabo	54	NA	NA	NA	NA	NA	NA	NA	26
Lee *et al.*, 2025[25]	Cabo	12	0 (0)	0 (0)	0 (0)	10 (83.3)	10 (83.3)	1 (8.3)	5.4	11.2
Falette-Puisieux *et al.*, 2023[29]	Cabo	4	NA	NA	NA	NA	NA	NA	2.8	9.2

Cabo, cabozantinib; CR, complete response; DCR, disease control rate; Lenva, lenvatinib; mo, months; mR, modified RECIST; NA, not available; ORR, objective response rate; OS, overall survival; PD, progressive disease; PFS, progression-free survival; PR, partial response; R, RECIST 1.1; Regora, regorafenib; SD, stable disease; Sora, sorafenib.

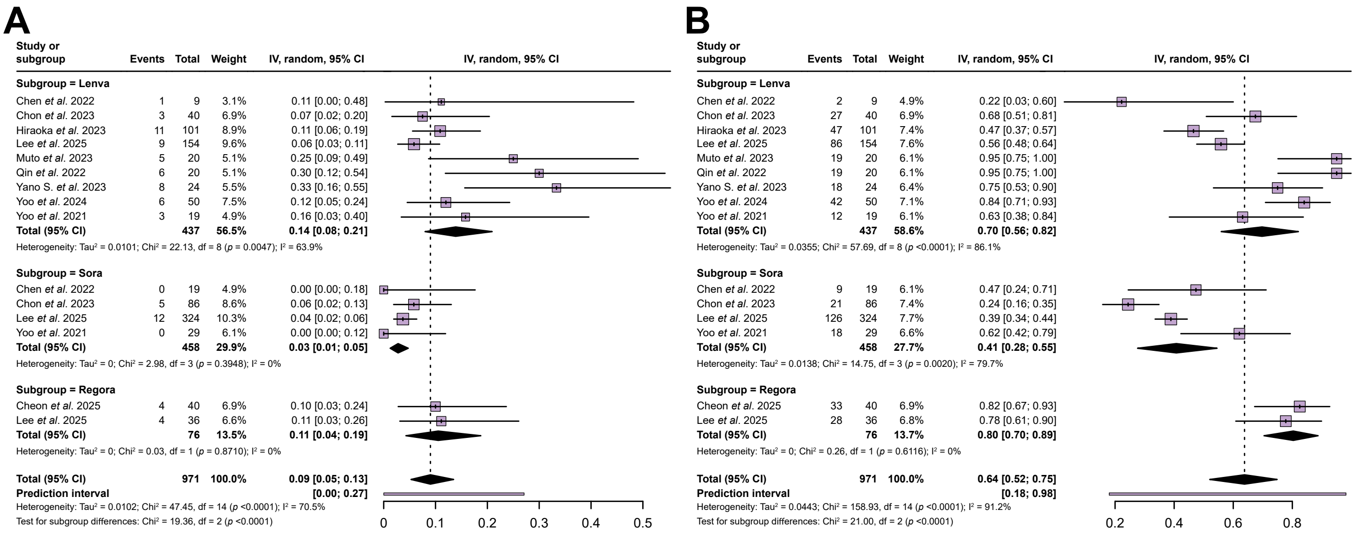

Fig. 5. Responses to lenvatinib (Lenva), sorafenib (Sora), and regorafenib (Regora). (A) ORR and (B) DCR with lenvatinib (Lenva), sorafenib (Sora), and regorafenib (Regora). Analyses were performed using proportion and inverse variance methods, and the Freeman–Tukey transformation, in random-effects models. Significance level was p <0.05. DCR, disease control rate; ORR, objective response rate.

possible; however, these rely on strong and unverifiable assumptions and could introduce additional bias due to incomplete adjustment and marked reductions in effective sample size. As another limitation, there was a potential risk of overlapping patient data if an institution contributed to the multicenter cohorts. However, sensitivity analyses excluding multicountry studies yielded results consistent with the primary analysis, supporting the robustness of the findings, yet do not eliminate the potential limitation completely. The majority of studies originated from Asia, which may limit the generalizability of the findings. Lastly, grading and reporting of toxicities in retrospective studies are not reliable, and few grade 3 and 4 toxicities were reported in the included studies.[44] Therefore, our analyses pooled any grade adverse events. However, in practice, grade 3 or 4 toxicities are more relevant. Finally, the atezolizumab-bevacizumab was the first-line treatment in most of the studies; therefore, the results of this study may be interpreted in this setting. More data are required after IO-IO combinations. Whether IO rechallenge instead of a TKI monotherapy would provide additional benefit in the second line is a future research topic.

Conclusions

In summary, this reconstructed IPD meta-analysis suggests heterogeneous survival outcomes with second-line TKIs after immunotherapy-based first-line treatment in advanced HCC. Across included retrospective studies, lenvatinib and regorafenib, showed consistent survival patterns compared with sorafenib, although these findings should be interpreted cautiously given baseline imbalances, heterogeneity, and the absence of patient-level adjustment. Data for cabozantinib remains limited and requires further investigation. Overall, these results are exploratory and hypothesis-generating; prospective studies and real-world registries are urgently needed to refine treatment sequencing and evaluate whether IO rechallenge, or novel combinations, may further improve outcomes.

Affiliations

[1]*Ankara University, Faculty of Medicine, Department of Medical Oncology, Ankara, Türkiye;* [2]*Cancer Research Institute, Ankara University, Ankara, Türkiye;* [3]*Université Paris Cité, INSERM, Centre de recherche sur l'inflammation, Paris, France;* [4]*Service de Chirurgie Hépato-Bilio-Pancréatique et Transplantation Hépatique, AP-HP, Hôpital Beaujon, Clichy, France;* [5]*INSERM, University of Rennes, Department of Medical Oncology, CLCC Eugène Marquis, COSS [(Chemistry Oncogenesis Stress Signaling)], Rennes, France;* [6]*AP-HP, Hôpital Beaujon, Liver Cancer and Innovative Therapy, Clichy, France;* [7]*INSERM U1149, Centre de Recherche sur l'Inflammation (CRI), Paris, France;* [8]*AP-HP Sorbonne Université, Hôpital Universitaire Pitié-Salpêtrière, Service d'Hépato-gastroentérologie, Paris, France;* [9]*INSERM UMR 1138, Centre de Recherche des Cordeliers, Paris, France;* [10]*Service d'Hépatologie, AP-HP, Hôpital Henri Mondor, Créteil, France;* [11]*AP-HP Sorbonne Université, Hôpital Universitaire Saint-Antoine, Service D'hépatologie, Paris, France;* [12]*INSERM UMRS 938 - Centre de Recherche Saint-Antoine (CRSA), Paris, France;* [13]*APHP, Hôpital Avicenne, Liver Unit, Hôpitaux Universitaires Paris-Seine-Saint-Denis, Université Paris Sorbonne Nord Bobigny, Paris, France*

Abbreviations

ASCO, American Society of Clinical Oncology; BCLC, Barcelona Clinic Liver Cancer; DCRs, disease control rates; ECOG-PS, Eastern Cooperative Oncology Group Performance Score; ESMO, European Society of Medical Oncology; HCC, hepatocellular carcinoma; HRs, hazard ratios; IO, immunotherapy; MVI, macrovascular invasion; NMA, network meta-analyses; ORRs, Objective response rates; OS, overall survival; PFS, progression-free survival; PRISMA, Preferred Reporting Items for Systematic Reviews and Meta-Analyses; RMSTs, restricted mean survival times; TKI, tyrosine kinase inhibitor.

Financial support

No financial support was received to produce this manuscript.

Conflicts of interest

MB declares consulting fees from Bayer, MSD, Sirtex Medical and Roche; advisory board fees from Bayer, MSD, Sirtex Medical, Eisai, AstraZeneca, Ipsen, Servier, Taiho, BMS and Terumo; and payment or honoraria for lectures from Bayer, Roche; MSD, Sirtex Medical, and AstraZeneca. JCN received research grants from Bayer and Ipsen. The remaining authors declare no conflicts of interest.
Please refer to the accompanying ICMJE disclosure forms for further details.

Authors' contributions

Conception or design of the work: EA, MB. Interpretation of data for the work: all authors. Drafting the work or reviewing it critically for important intellectual content: all authors. Final approval of the version to be published: all authors.

Data availability

All data analyzed in this study were derived from previously published articles, which are cited in the reference list.

Supplementary data

Supplementary data to this article can be found online at https://doi.org/10.1016/j.jhepr.2026.101893.

References

[1] Llovet JM, Kelley RK, Villanueva A, et al. Hepatocellular carcinoma. Nat Rev Dis Primers 2021;7:6.
[2] Siegel RL, Giaquinto AN, Jemal A. Cancer statistics, 2024. CA Cancer J Clin 2024;74:12–49.
[3] Llovet JM, Ricci S, Mazzaferro V, et al. Sorafenib in advanced hepatocellular carcinoma. N Engl J Med 2008;359:378–390.
[4] Kudo M, Finn RS, Qin S, et al. Lenvatinib versus sorafenib in first-line treatment of patients with unresectable hepatocellular carcinoma: a randomised phase 3 non-inferiority trial. Lancet 2018;391:1163–1173.
[5] Bruix J, Qin S, Merle P, et al. Regorafenib for patients with hepatocellular carcinoma who progressed on sorafenib treatment (RESORCE): a randomised, double-blind, placebo-controlled, phase 3 trial. Lancet 2017;389:56–66.
[6] Zhu AX, Kang YK, Yen CJ, et al. Ramucirumab after sorafenib in patients with advanced hepatocellular carcinoma and increased α-fetoprotein concentrations (REACH-2): a randomised, double-blind, placebo-controlled, phase 3 trial. Lancet Oncol 2019;20:282–296.
[7] Abou-Alfa GK, Meyer T, Cheng AL, et al. Cabozantinib in patients with advanced and progressing hepatocellular carcinoma. N Engl J Med 2018;379:54–63.
[8] Vogel A, Chan SL, Dawson LA, et al. Hepatocellular carcinoma: ESMO Clinical Practice Guideline for diagnosis, treatment and follow-up. Ann Oncol 2025;36:491–506.
[9] Cheng AL, Qin S, Ikeda M, et al. Updated efficacy and safety data from IMbrave150: atezolizumab plus bevacizumab vs. sorafenib for unresectable hepatocellular carcinoma. J Hepatol 2022;76:862–873.
[10] Qin S, Chan SL, Gu S, et al. Camrelizumab plus rivoceranib versus sorafenib as first-line therapy for unresectable hepatocellular carcinoma (CARES-310): a randomised, open-label, international phase 3 study. Lancet 2023;402:1133–1146.
[11] Sangro B, Chan SL, Kelley RK, et al. Four-year overall survival update from the phase III HIMALAYA study of tremelimumab plus durvalumab in unresectable hepatocellular carcinoma. Ann Oncol 2024;35:448–457.
[12] Yau T, Galle PR, Decaens T, et al. Nivolumab plus ipilimumab versus lenvatinib or sorafenib as first-line treatment for unresectable hepatocellular carcinoma (CheckMate 9DW): an open-label, randomised, phase 3 trial. Lancet 2025;405:1851–1864.
[13] Page MJ, McKenzie JE, Bossuyt PM, et al. The PRISMA 2020 statement: an updated guideline for reporting systematic reviews. BMJ 2021;372:n71.
[14] Page MJ, Sterne JAC, Boutron I, et al. ROB-ME: a tool for assessing risk of bias due to missing evidence in systematic reviews with meta-analysis. BMJ 2023;383:e076754.
[15] Alsinbili A. Assessing the risk of bias in single-arm trials for systematic reviews: moving towards a more reliable evaluation of clinical evidence. Future Healthc J 2023;10(Suppl. 3):26–27.
[16] Guyot P, Ades AE, Ouwens MJ, et al. Enhanced secondary analysis of survival data: reconstructing the data from published Kaplan-Meier survival curves. BMC Med Res Methodol 2012;12:9.
[17] Grambsch PM, Therneau TM. Proportional hazards tests and diagnostics based on weighted residuals. Biometrika 1994;81:515–526.
[18] Royston P, Parmar MK. Restricted mean survival time: an alternative to the hazard ratio for the design and analysis of randomized trials with a time-to-event outcome. BMC Med Res Methodol 2013;13:152.
[19] Freeman MF, Tukey JW. Transformations related to the angular and the square root. Ann Math Stat 1950;21:607–611. 5.
[20] Viechtbauer W. Conducting meta-analyses in R with the metafor package. J Stat Soft 2010;36:1–48.
[21] Chen CT, Feng YH, Yen CJ, et al. Prognosis and treatment pattern of advanced hepatocellular carcinoma after failure of first-line atezolizumab and bevacizumab treatment. Hepatol Int 2022;16:1199–1207.
[22] Chon YE, Kim DY, Kim MN, et al. Sorafenib vs. lenvatinib in advanced hepatocellular carcinoma after atezolizumab/bevacizumab failure: a real-world study. Clin Mol Hepatol 2024;30:345–359.
[23] Decraecker M, Edeline J, Thiam EM, et al. 153P Evolution of second-line practices in the era of immunotherapy: real-life data from the French prospective CHIEF cohort. Ann Oncol 2025;36:S65–S66.
[24] Hiraoka A, Kumada T, Tada T, et al. Lenvatinib as second-line treatment after atezolizumab plus bevacizumab for unresectable hepatocellular carcinoma: clinical results show importance of hepatic reserve function. Oncology 2023;101:624–633.
[25] Lee CK, Yoo C, Hong JY, et al. Real-world study of systemic treatment after first-line atezolizumab plus bevacizumab for hepatocellular carcinoma in Asia-Pacific countries. Liver Cancer 2025;14:127–141.
[26] Lombardi P, Yang H, Manfredi GF, et al. 175P Lenvatinib versus sorafenib as second-line treatment post atezolizumab plus bevacizumab for hepatocellular carcinoma: the LEVIATHAN study. Ann Oncol 2025;36:S73–S74.
[27] Muto H, Kuzuya T, Kawabe N, et al. Clinical outcomes with lenvatinib in patients previously treated with atezolizumab/bevacizumab for advanced hepatocellular carcinoma. Anticancer Res 2023;43:4673–4682.
[28] Persano M, Casadei-Gardini A, Tada T, et al. Lenvatinib versus sorafenib second-line therapy in patients with hepatocellular carcinoma progressed to atezolizumab plus bevacizumab: a retrospective real-world study. Oncology 2025;103:456–468.
[29] Falette-Puisieux M, Nault JC, Bouattour M, et al. Beyond atezolizumab plus bevacizumab in patients with advanced hepatocellular carcinoma: overall efficacy and safety of tyrosine kinase inhibitors in a real-world setting. Ther Adv Med Oncol 2023;15:17588359231189425.
[30] Qin HN, Ning Z, Sun R, et al. Lenvatinib as second-line treatment in patients with unresectable hepatocellular carcinoma: a retrospective analysis. Front Oncol 2022;12:1003426.
[31] Yano S, Kawaoka T, Yamasaki S, et al. Therapeutic efficacy and safety of lenvatinib after atezolizumab plus bevacizumab for unresectable hepatocellular carcinoma. Cancers (Basel) 2023;15:5406.
[32] Yoo C, Kim HD, Chon HJ, et al. LBA1 Multicenter phase II trial of lenvatinib in patients with advanced hepatocellular carcinoma after progression on first-line atezolizumab plus bevacizumab (KCSG HB23-04). Ann Oncol 2024;35:S1450.
[33] Yoo C, Kim JH, Ryu MH, et al. Clinical outcomes with multikinase inhibitors after progression on first-line atezolizumab plus bevacizumab in patients with advanced hepatocellular carcinoma: a multinational multicenter retrospective study. Liver Cancer 2021;10:107–114.
[34] Möhring C, Berger M, Sadeghlar F, et al. Evaluating sorafenib (SORA-2) as second-line treatment for unresectable hepatocellular carcinoma: a European retrospective multicenter study. Cancers 2025;17:972.
[35] Cheon J, Ryoo BY, Chon HJ, et al. Multicenter phase 2 trial of second-line regorafenib in patients with unresectable hepatocellular carcinoma after progression on atezolizumab plus bevacizumab. Liver Cancer 2025;14:446–455.
[36] Ahn DH, Park NJ, Locker M, et al. Real-world clinical outcomes of cabozantinib as a second-line treatment for advanced hepatocellular carcinoma: a retrospective US claims analysis. Oncologist 2025;30. oyaf252.
[37] Lombardi P, Kim JS, Manfredi GF, et al. Lenvatinib vs. sorafenib as second-line treatment post atezolizumab plus bevacizumab for hepatocellular carcinoma: the LEVIATHAN study. JHEP Rep 2025;7:101595.
[38] Kim H-D, Sym SJ, Chon HJ, et al. Multicenter single-arm phase II trial of lenvatinib in patients with advanced hepatocellular carcinoma after

progression on first-line atezolizumab plus bevacizumab. J Hepatol 2026;84:308–315.
[39] Hsieh MC, Su YL, Chen YY, et al. Lenvatinib acts as the salvage therapy for advanced hepatocellular carcinoma. Ann Oncol 2018;29. ix57.
[40] Chan LL, Kwong TT, Yau JCW, et al. Treatment for hepatocellular carcinoma after immunotherapy. Ann Hepatol 2025;30:101781.
[41] Chan SL, Ryoo BY, Mo F, et al. Multicentre phase II trial of cabozantinib in patients with hepatocellular carcinoma after immune checkpoint inhibitor treatment. J Hepatol 2024;81:258–264.
[42] Llovet JM, Kudo M, Merle P, et al. Lenvatinib plus pembrolizumab versus lenvatinib plus placebo for advanced hepatocellular carcinoma (LEAP-002): a randomised, double-blind, phase 3 trial. Lancet Oncol 2023;24:1399–1410.
[43] Cabibbo G, Reig M, Celsa C, et al. First-Line immune checkpoint inhibitor-based sequential therapies for advanced hepatocellular carcinoma: rationale for future trials. Liver Cancer 2022;11:75–84.
[44] Campani C, Pallas D, Sidali S, et al. Heterogeneity in adverse events related to atezolizumab-bevacizumab for hepatocellular carcinoma reported in real-world studies. JHEP Rep 2024;6:101190.

Keywords: Hepatocellular carcinoma; Tyrosine kinase inhibitor; Atezolizumab-bevacizumab; Lenvatinib; Regorafenib; Sorafenib.
Received 16 December 2025; received in revised form 29 April 2026; accepted 5 May 2026; Available online 12 May 2026

Journal of Hepatology, Volume *8*

Supplemental information

Second-line TKI after first-line immunotherapy-based treatment in advanced HCC: Reconstructed IPD meta-analysis

Erman Akkus, Christian Hobeika, Julien Edeline, Clémence Hollande, Manon Allaire, Giuliana Amaddeo, Hélène Regnault, Marie Lequoy, Jean Charles Nault, and Mohamed Bouattour

Second-line TKI after first-line immunotherapy-based treatment in advanced HCC: Reconstructed IPD meta-analysis

Erman Akkus, Christian Hobeika, Julien Edeline, Clémence Hollande, Manon Allaire, Giuliana Amaddeo, Hélène Regnault, Marie Lequoy, Jean Charles Nault, Mohamed Bouattour

Table of contents

Supplementary figures……………………………………………………………...2

Supplementary tables……………………………………………………………..19

Supplementary figures

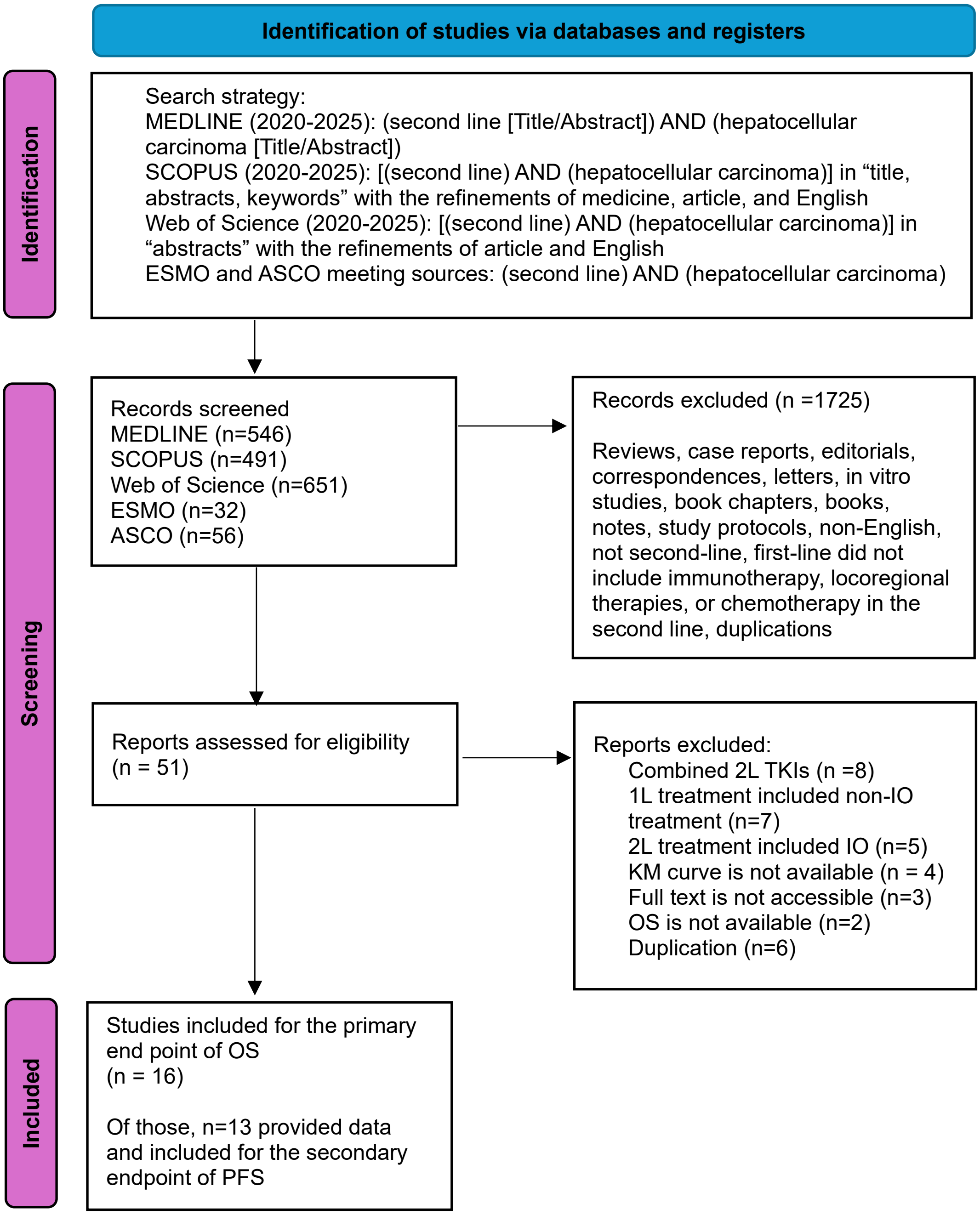

Fig. S1. PRISMA diagram of the study selection. ESMO: European Society of Medical Oncology, ASCO: American Society of Clinical Oncology, OS: Overall survival, PFS: Progression-free survival, TKI: tyrosine-kinase inhibitor, 1L: first-line, 2L: second-line, IO: immunotherapy, KM: Kaplan-Meier

Original OS graph: **Reconstructed OS graph:**

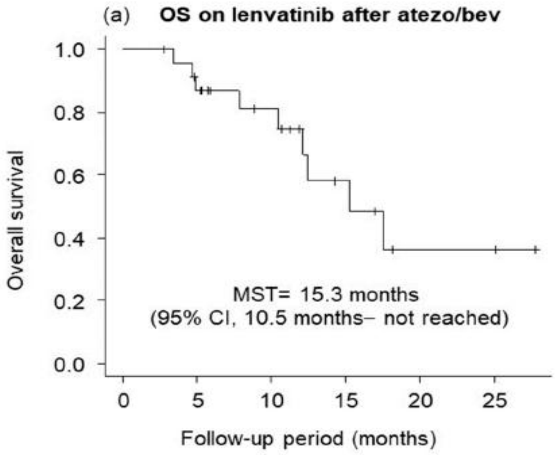

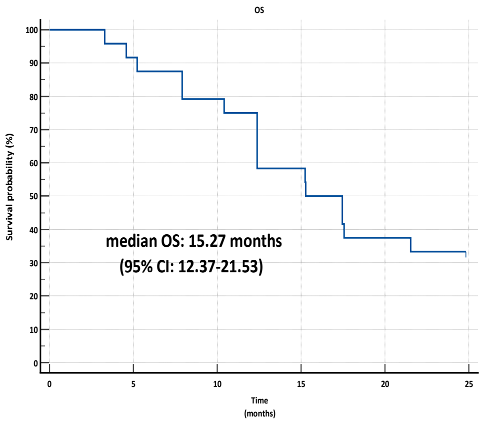

Fig. S2. Sample representation of reconstructed IPD data from published KM curves. Study: Yano S. et al, 2023, Lenvatinib

a) Percentage of patients with *ECOG PS-0.* *No difference.*

Study or Subgroup	Events	Total	Weight	IV, Random, 95% CI
**Subgroup = Lenva**				
Chon et al. 2023	26	40	5.9%	0.65 [0.48; 0.79]
Hiraoka et al. 2023	76	101	6.5%	0.75 [0.66; 0.83]
Lee et al. 2025	77	154	6.6%	0.50 [0.42; 0.58]
Lombardi et al. 2025	69	125	6.6%	0.55 [0.46; 0.64]
Muto et al. 2023	12	20	5.2%	0.60 [0.36; 0.81]
Piusieux et al. 2025	0	8	3.8%	0.00 [0.00; 0.37]
Yano S. et al. 2023	19	24	5.4%	0.79 [0.58; 0.93]
Yoo et al. 2024	9	50	6.1%	0.18 [0.09; 0.31]
Yoo et al. 2021	6	19	5.1%	0.32 [0.13; 0.57]
**Total (95% CI)**		**541**	**51.3%**	**0.49 [0.35; 0.64]**
Heterogeneity: $Tau^2 = 0.0389$; $Chi^2 = 77.11$, df = 8 ($P < 0.0001$); $I^2 = 89.6\%$				
**Subgroup = Sora**				
Chon et al. 2023	43	86	6.4%	0.50 [0.39; 0.61]
Lee et al. 2025	124	324	6.8%	0.38 [0.33; 0.44]
Lombardi et al. 2025	33	105	6.5%	0.31 [0.23; 0.41]
Piusieux et al. 2025	5	41	5.9%	0.12 [0.04; 0.26]
Yoo et al. 2021	5	29	5.6%	0.17 [0.06; 0.36]
**Total (95% CI)**		**585**	**31.3%**	**0.30 [0.20; 0.42]**
Heterogeneity: $Tau^2 = 0.0146$; $Chi^2 = 25.8$, df = 4 ($P < 0.0001$); $I^2 = 84.5\%$				
**Subgroup = Regora**				
Cheon et al. 2025	24	40	5.9%	0.60 [0.43; 0.75]
Lee et al. 2025	15	36	5.8%	0.42 [0.26; 0.59]
Piusieux et al. 2025	1	29	5.6%	0.03 [0.00; 0.18]
**Total (95% CI)**		**105**	**17.4%**	**0.32 [0.04; 0.70]**
Heterogeneity: $Tau^2 = 0.1022$; $Chi^2 = 30.79$, df = 2 ($P < 0.0001$); $I^2 = 93.5\%$				
**Total (95% CI)**		**1231**	**100.0%**	**0.40 [0.30; 0.50]**
**Prediction interval**				**[0.06; 0.81]**
Heterogeneity: $Tau^2 = 0.0368$; $Chi^2 = 176.39$, df = 16 ($P < 0.0001$); $I^2 = 90.9\%$				
Test for subgroup differences: $Chi^2 = 4.16$, df = 2 ($P = 0.1250$)				

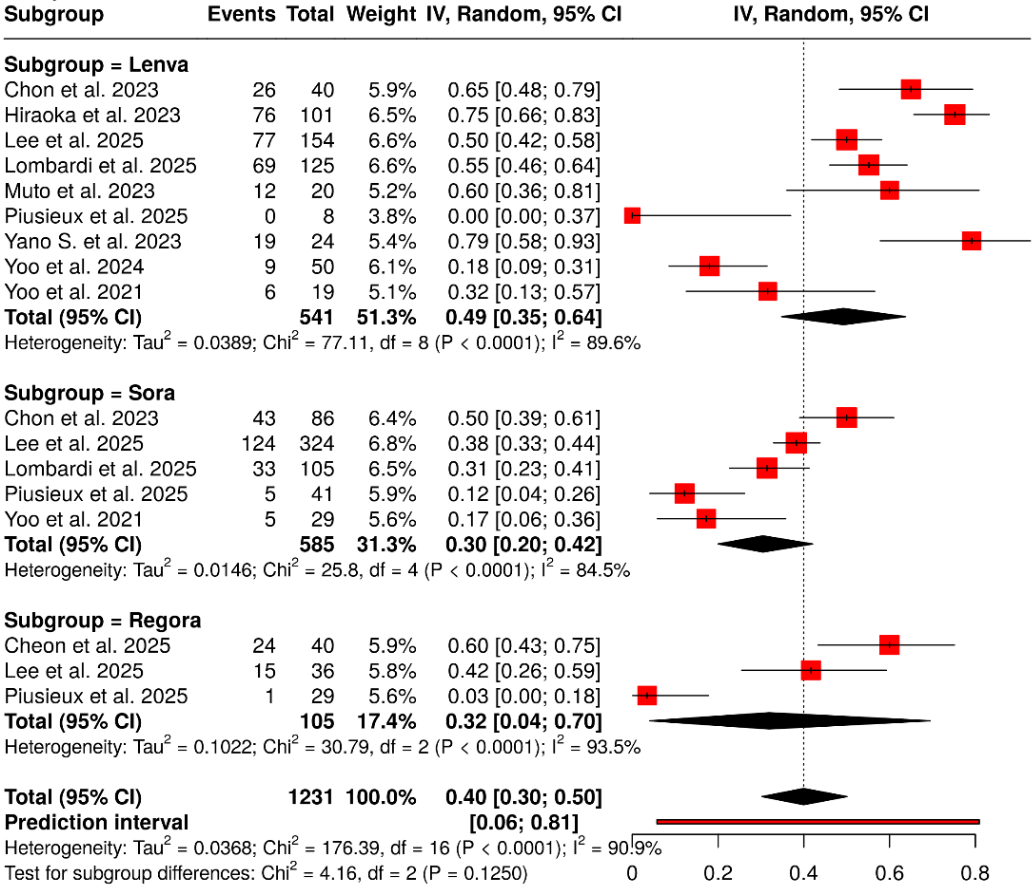

b) Percentage of patients with *Child-Pugh Class A.* *The regorafenib group had a significantly higher rate of CP-A*.

Study or Subgroup	Events	Total	Weight	IV, Random, 95% CI
**Subgroup = Lenva**				
Chen et al. 2022	6	9	3.5%	0.67 [0.30; 0.93]
Chon et al. 2023	37	40	5.2%	0.93 [0.80; 0.98]
Hiraoka et al. 2023	82	101	5.7%	0.81 [0.72; 0.88]
Lee et al. 2025	135	154	5.8%	0.88 [0.81; 0.92]
Muto et al. 2023	16	20	4.6%	0.80 [0.56; 0.94]
Persano et al. 2024	85	86	5.7%	0.99 [0.94; 1.00]
Piusieux et al. 2025	3	8	3.4%	0.38 [0.09; 0.76]
Qin et al. 2022	12	20	4.6%	0.60 [0.36; 0.81]
Yano S. et al. 2023	16	24	4.8%	0.67 [0.45; 0.84]
Yoo et al. 2024	50	50	5.4%	1.00 [0.93; 1.00]
Yoo et al. 2021	19	19	4.5%	1.00 [0.82; 1.00]
**Total (95% CI)**		**531**	**53.0%**	**0.86 [0.76; 0.94]**
Heterogeneity: Tau^2 = 0.0352; Chi^2 = 73.06, df = 10 (P < 0.0001); I^2 = 86.3%				
**Subgroup = Sora**				
Chen et al. 2022	15	19	4.5%	0.79 [0.54; 0.94]
Chon et al. 2023	54	86	5.7%	0.63 [0.52; 0.73]
Lee et al. 2025	235	324	6.0%	0.73 [0.67; 0.77]
Persano et al. 2024	48	51	5.4%	0.94 [0.84; 0.99]
Piusieux et al. 2025	34	41	5.2%	0.83 [0.68; 0.93]
Yoo et al. 2021	29	29	4.9%	1.00 [0.88; 1.00]
**Total (95% CI)**		**550**	**31.7%**	**0.84 [0.71; 0.94]**
Heterogeneity: Tau^2 = 0.0288; Chi^2 = 44.1, df = 5 (P < 0.0001); I^2 = 88.7%				
**Subgroup = Regora**				
Cheon et al. 2025	40	40	5.2%	1.00 [0.91; 1.00]
Lee et al. 2025	34	36	5.1%	0.94 [0.81; 0.99]
Piusieux et al. 2025	27	29	4.9%	0.93 [0.77; 0.99]
**Total (95% CI)**		**105**	**15.3%**	**0.97 [0.90; 1.00]**
Heterogeneity: Tau^2 = 0.0069; Chi^2 = 3.94, df = 2 (P = 0.1396); I^2 = 49.2%				
**Total (95% CI)**		**1186**	**100.0%**	**0.88 [0.80; 0.93]**
**Prediction interval**				**[0.50; 1.00]**
Heterogeneity: Tau^2 = 0.0358; Chi^2 = 170.09, df = 19 (P < 0.0001); I^2 = 88.8%				
Test for subgroup differences: Chi^2 = 6.10, df = 2 (P = 0.0473)				

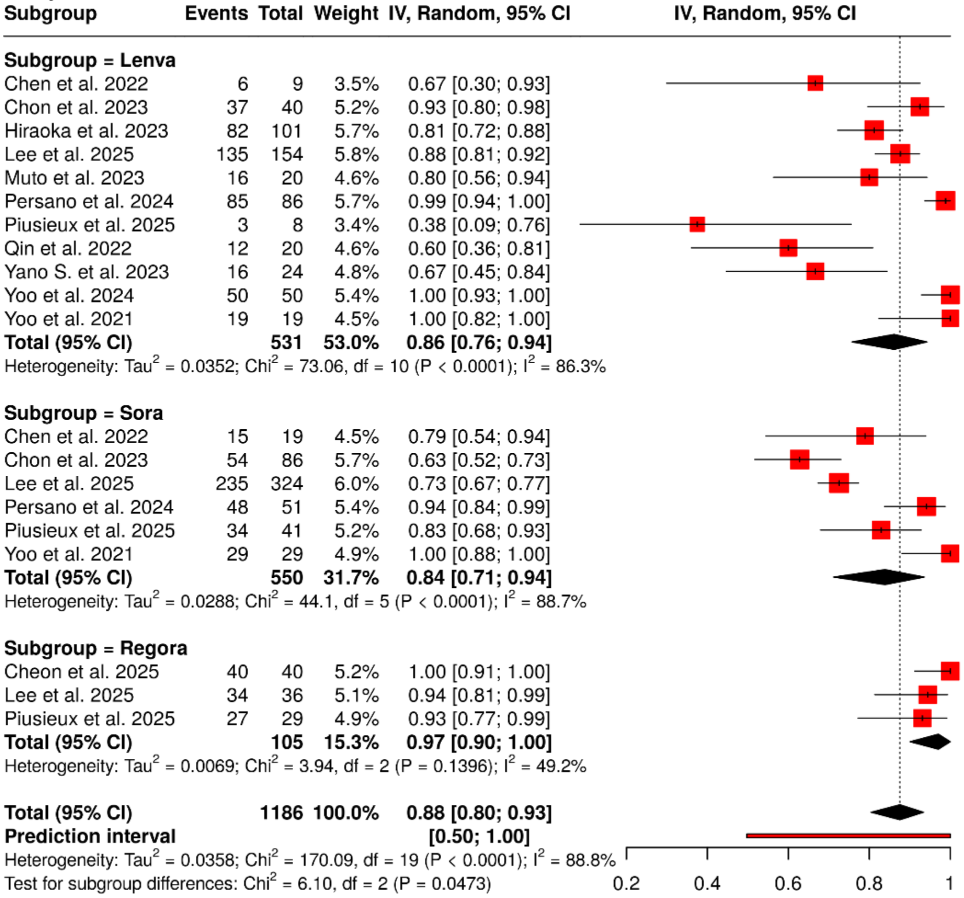

c) Percentage of patients with ***BCLC stage C.*** *No difference.*

Study or Subgroup	Events	Total	Weight	IV, Random, 95% CI
**Subgroup = Lenva**				
Chon et al. 2023	36	40	5.0%	0.90 [0.76; 0.97]
Hiraoka et al. 2023	65	101	5.6%	0.64 [0.54; 0.74]
Lee et al. 2025	126	154	5.7%	0.82 [0.75; 0.88]
Lombardi et al. 2025	56	125	5.7%	0.45 [0.36; 0.54]
Muto et al. 2023	14	20	4.3%	0.70 [0.46; 0.88]
Persano et al. 2024	53	86	5.5%	0.62 [0.51; 0.72]
Piusieux et al. 2025	6	8	3.1%	0.75 [0.35; 0.97]
Qin et al. 2022	20	20	4.3%	1.00 [0.83; 1.00]
Yano S. et al. 2023	16	24	4.5%	0.67 [0.45; 0.84]
Yoo et al. 2024	38	50	5.2%	0.76 [0.62; 0.87]
Yoo et al. 2021	19	19	4.3%	1.00 [0.82; 1.00]
**Total (95% CI)**		**647**	**53.3%**	**0.78 [0.66; 0.88]**
Heterogeneity: Tau^2 = 0.0381; Chi^2 = 94.53, df = 10 (P < 0.0001); I^2 = 89.4%				
**Subgroup = Sora**				
Chon et al. 2023	73	86	5.5%	0.85 [0.76; 0.92]
Lee et al. 2025	246	324	5.9%	0.76 [0.71; 0.80]
Lombardi et al. 2025	71	105	5.6%	0.68 [0.58; 0.76]
Persano et al. 2024	34	51	5.2%	0.67 [0.52; 0.79]
Piusieux et al. 2025	39	41	5.0%	0.95 [0.83; 0.99]
Yoo et al. 2021	19	29	4.7%	0.66 [0.46; 0.82]
**Total (95% CI)**		**636**	**32.0%**	**0.77 [0.68; 0.85]**
Heterogeneity: Tau^2 = 0.0107; Chi^2 = 23.68, df = 5 (P = 0.0003); I^2 = 78.9%				
**Subgroup = Regora**				
Cheon et al. 2025	39	40	5.0%	0.97 [0.87; 1.00]
Lee et al. 2025	12	36	4.9%	0.33 [0.19; 0.51]
Piusieux et al. 2025	27	29	4.7%	0.93 [0.77; 0.99]
**Total (95% CI)**		**105**	**14.7%**	**0.80 [0.33; 1.00]**
Heterogeneity: Tau^2 = 0.1710; Chi^2 = 50.18, df = 2 (P < 0.0001); I^2 = 96%				
**Total (95% CI)**		**1388**	**100.0%**	**0.78 [0.70; 0.85]**
**Prediction interval**				**[0.40; 1.00]**
Heterogeneity: Tau^2 = 0.0309; Chi^2 = 174.65, df = 19 (P < 0.0001); I^2 = 89.1%				
Test for subgroup differences: Chi^2 = 0.01, df = 2 (P = 0.9941)				

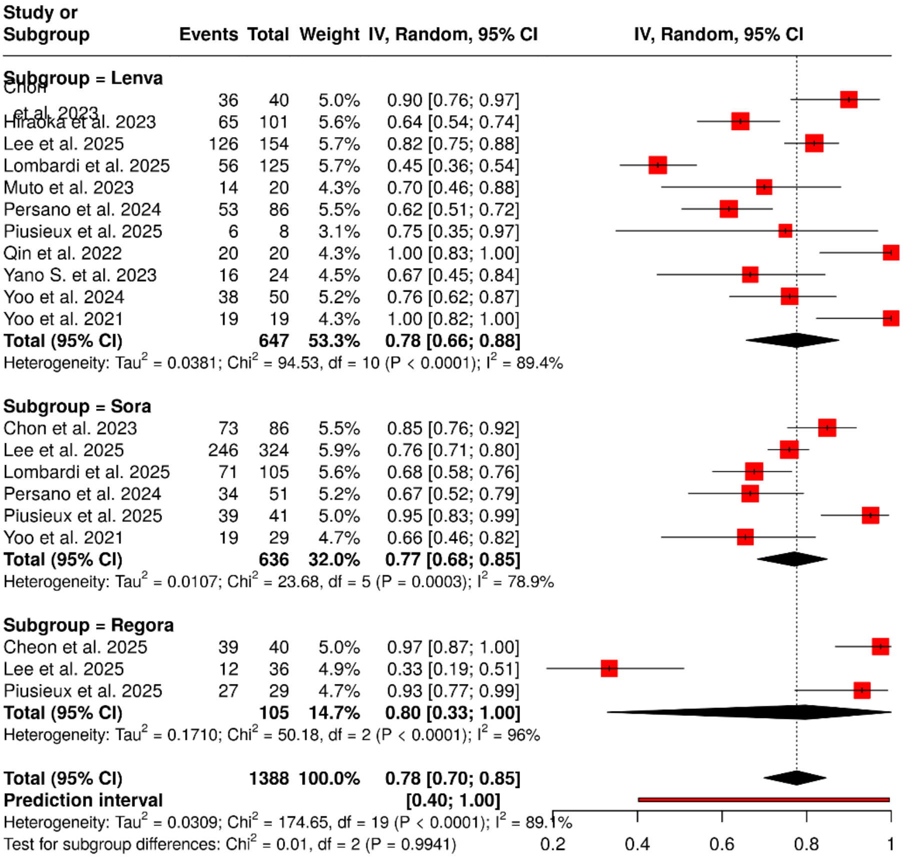

d) Percentage of patients with *ALBI grade 1. No difference.*

Study or Subgroup	Events	Total	Weight	IV, Random, 95% CI
**Subgroup = Lenva**				
Chen et al. 2022	1	9	7.4%	0.11 [0.00; 0.48]
Hiraoka et al. 2023	24	101	8.8%	0.24 [0.16; 0.33]
Lombardi et al. 2025	92	125	8.8%	0.74 [0.65; 0.81]
Muto et al. 2023	6	20	8.1%	0.30 [0.12; 0.54]
Persano et al. 2024	82	86	8.8%	0.95 [0.89; 0.99]
Piusieux et al. 2025	1	8	7.2%	0.12 [0.00; 0.53]
**Total (95% CI)**		**349**	**49.1%**	**0.44 [0.14; 0.77]**
Heterogeneity: $Tau^2 = 0.1545$; $Chi^2 = 162.59$, df = 5 ($P < 0.0001$); $I^2 = 96.9\%$				
**Subgroup = Sora**				
Chen et al. 2022	5	19	8.1%	0.26 [0.09; 0.51]
Lombardi et al. 2025	49	105	8.8%	0.47 [0.37; 0.57]
Persano et al. 2024	49	51	8.6%	0.96 [0.87; 1.00]
Piusieux et al. 2025	6	41	8.5%	0.15 [0.06; 0.29]
**Total (95% CI)**		**216**	**34.0%**	**0.48 [0.12; 0.85]**
Heterogeneity: $Tau^2 = 0.1571$; $Chi^2 = 94.21$, df = 3 ($P < 0.0001$); $I^2 = 96.8\%$				
**Subgroup = Regora**				
Cheon et al. 2025	17	40	8.5%	0.42 [0.27; 0.59]
Piusieux et al. 2025	6	29	8.4%	0.21 [0.08; 0.40]
**Total (95% CI)**		**69**	**16.9%**	**0.32 [0.13; 0.54]**
Heterogeneity: $Tau^2 = 0.0187$; $Chi^2 = 3.55$, df = 1 ($P = 0.0595$); $I^2 = 71.8\%$				
**Total (95% CI)**		**634**	**100.0%**	**0.44 [0.24; 0.64]**
**Prediction interval**				**[0.00; 1.00]**
Heterogeneity: $Tau^2 = 0.1206$; $Chi^2 = 280.17$, df = 11 ($P < 0.0001$); $I^2 = 96.1\%$				
Test for subgroup differences: $Chi^2 = 0.71$, df = 2 ($P = 0.7023$)				

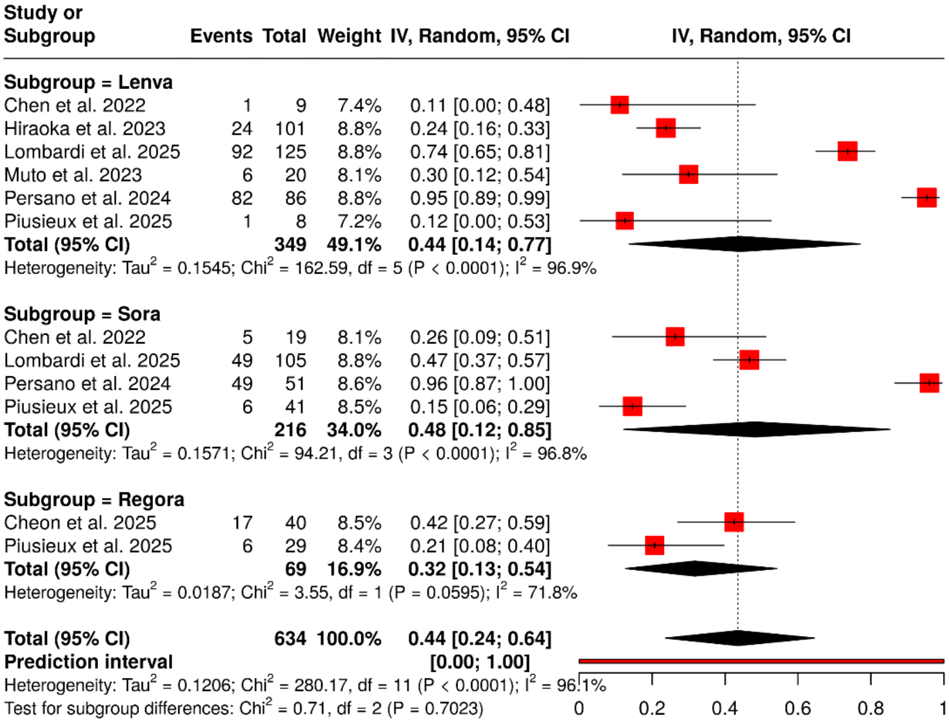

e) Percentage of patients with *macrovascular invasion (MVI)*. *The regorafenib group had significantly less MVI.*

Study or Subgroup	Events	Total	Weight	IV, Random, 95% CI
**Subgroup = Lenva**				
Chen et al. 2022	9	9	3.4%	1.00 [0.66; 1.00]
Chon et al. 2023	17	40	6.6%	0.42 [0.27; 0.59]
Hiraoka et al. 2023	30	101	7.9%	0.30 [0.21; 0.40]
Lee et al. 2025	42	154	8.3%	0.27 [0.20; 0.35]
Piusieux et al. 2025	2	8	3.2%	0.25 [0.03; 0.65]
Yano S. et al. 2023	8	24	5.6%	0.33 [0.16; 0.55]
Yoo et al. 2024	12	50	7.0%	0.24 [0.13; 0.38]
Yoo et al. 2021	9	19	5.1%	0.47 [0.24; 0.71]
**Total (95% CI)**		**405**	**47.0%**	**0.38 [0.27; 0.51]**
Heterogeneity: $Tau^2 = 0.0201$; $Chi^2 = 32.09$, df = 7 ($P < 0.0001$); $I^2 = 78.2\%$				
**Subgroup = Sora**				
Chen et al. 2022	14	19	5.1%	0.74 [0.49; 0.91]
Chon et al. 2023	41	86	7.7%	0.48 [0.37; 0.59]
Lee et al. 2025	90	324	8.7%	0.28 [0.23; 0.33]
Piusieux et al. 2025	20	41	6.6%	0.49 [0.33; 0.65]
Yoo et al. 2021	9	29	6.0%	0.31 [0.15; 0.51]
**Total (95% CI)**		**499**	**34.1%**	**0.44 [0.29; 0.59]**
Heterogeneity: $Tau^2 = 0.0227$; $Chi^2 = 28.55$, df = 4 ($P < 0.0001$); $I^2 = 86\%$				
**Subgroup = Regora**				
Cheon et al. 2025	10	40	6.6%	0.25 [0.13; 0.41]
Lee et al. 2025	5	36	6.4%	0.14 [0.05; 0.29]
Piusieux et al. 2025	8	29	6.0%	0.28 [0.13; 0.47]
**Total (95% CI)**		**105**	**18.9%**	**0.22 [0.14; 0.31]**
Heterogeneity: $Tau^2 = 0.0006$; $Chi^2 = 2.17$, df = 2 ($P = 0.3387$); $I^2 = 7.6\%$				
**Total (95% CI)**		**1009**	**100.0%**	**0.36 [0.29; 0.44]**
**Prediction interval**				**[0.12; 0.65]**
Heterogeneity: $Tau^2 = 0.0158$; $Chi^2 = 69.43$, df = 15 ($P < 0.0001$); $I^2 = 78.4\%$				
Test for subgroup differences: $Chi^2 = 8.75$, df = 2 ($P = 0.0126$)				

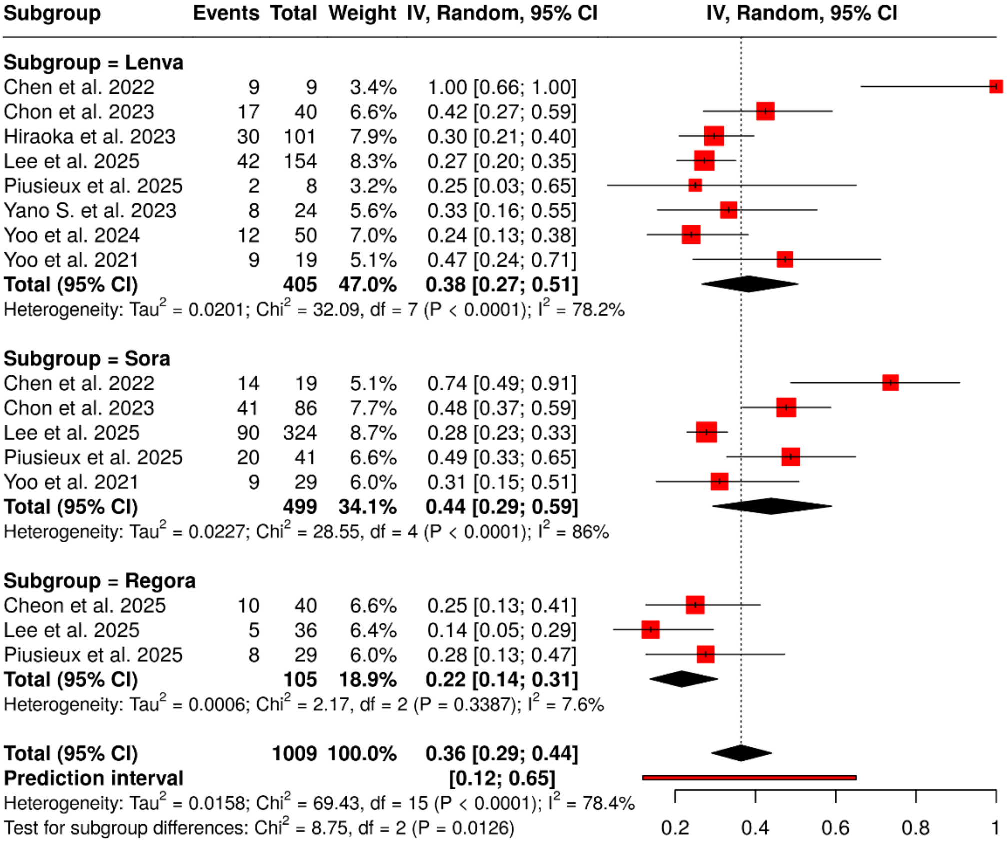

f) Percentage of patients with ***extrahepatic spread.*** *No difference.*

Study or Subgroup	Events	Total	Weight	IV, Random, 95% CI
**Subgroup = Lenva**				
Chen et al. 2022	5	9	3.2%	0.56 [0.21; 0.86]
Chon et al. 2023	29	40	6.1%	0.72 [0.56; 0.85]
Hiraoka et al. 2023	45	101	7.2%	0.45 [0.35; 0.55]
Lee et al. 2025	106	154	7.6%	0.69 [0.61; 0.76]
Lombardi et al. 2025	60	125	7.4%	0.48 [0.39; 0.57]
Muto et al. 2023	9	20	4.8%	0.45 [0.23; 0.68]
Piusieux et al. 2025	4	8	3.0%	0.50 [0.16; 0.84]
Qin et al. 2022	11	20	4.8%	0.55 [0.32; 0.77]
Yano S. et al. 2023	11	24	5.2%	0.46 [0.26; 0.67]
**Total (95% CI)**		**501**	**49.4%**	**0.55 [0.45; 0.64]**
Heterogeneity: $Tau^2 = 0.0111$; $Chi^2 = 25.82$, df = 8 (P = 0.0011); $I^2 = 69\%$				
**Subgroup = Sora**				
Chen et al. 2022	13	19	4.7%	0.68 [0.43; 0.87]
Chon et al. 2023	58	86	7.1%	0.67 [0.56; 0.77]
Lee et al. 2025	193	324	7.9%	0.60 [0.54; 0.65]
Lombardi et al. 2025	47	105	7.3%	0.45 [0.35; 0.55]
Piusieux et al. 2025	34	41	6.1%	0.83 [0.68; 0.93]
**Total (95% CI)**		**575**	**33.1%**	**0.64 [0.52; 0.75]**
Heterogeneity: $Tau^2 = 0.0129$; $Chi^2 = 22.57$, df = 4 (P = 0.0002); $I^2 = 82.3\%$				
**Subgroup = Regora**				
Cheon et al. 2025	34	40	6.1%	0.85 [0.70; 0.94]
Lee et al. 2025	11	36	5.9%	0.31 [0.16; 0.48]
Piusieux et al. 2025	25	29	5.5%	0.86 [0.68; 0.96]
**Total (95% CI)**		**105**	**17.5%**	**0.69 [0.31; 0.97]**
Heterogeneity: $Tau^2 = 0.1057$; $Chi^2 = 31.78$, df = 2 (P < 0.0001); $I^2 = 93.7\%$				
**Total (95% CI)**		**1181**	**100.0%**	**0.60 [0.53; 0.68]**
**Prediction interval**				**[0.31; 0.86]**
Heterogeneity: $Tau^2 = 0.0169$; $Chi^2 = 86.06$, df = 16 (P < 0.0001); $I^2 = 81.4\%$				
Test for subgroup differences: $Chi^2 = 1.75$, df = 2 (P = 0.4164)				

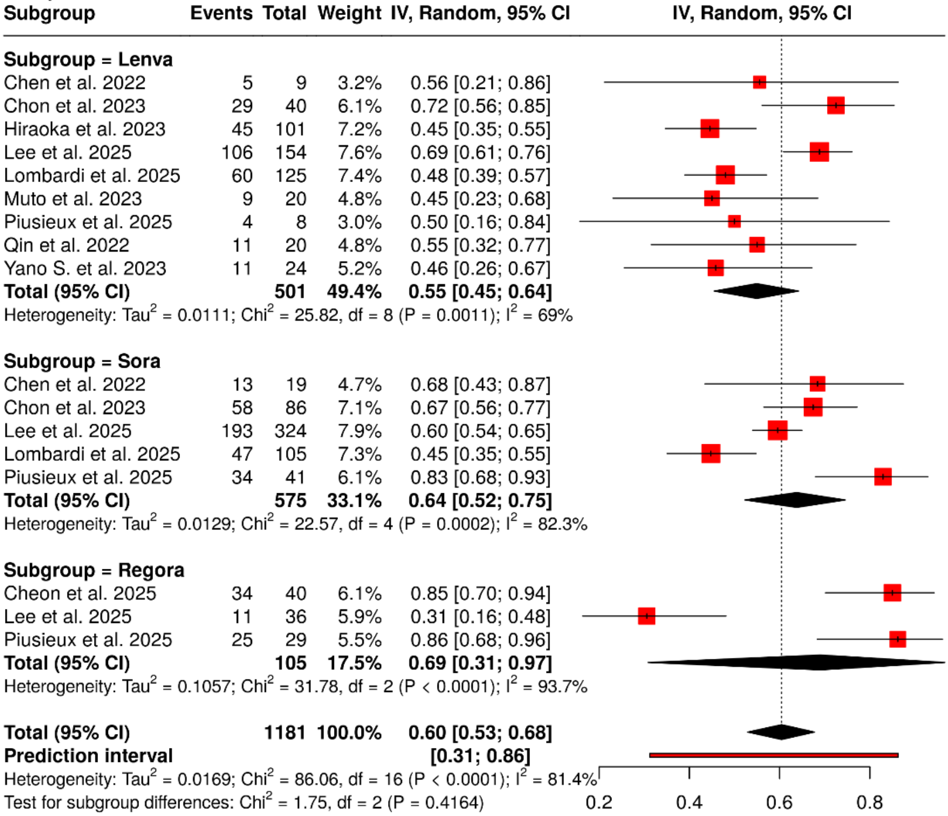

Fig. S3. Pooled baseline characteristics and comparison between Lenva (lenvatinib), Sora (sorafenib), and Regora (regorafenib). a*) European Cooperation Oncology Group performance score (ECOG-PS) 0, b) Child-Pugh (CP) A, c) Barcelona Clinic Liver Cancer (BCLC) C, d) ALBI grade 1, e) Macrovascular invasion (MVI), f) Extrahepatic spread*

OS

Chisq: 17.7

df: 2

p: 0.00014

PFS

Chisq: 28.8

df: 2

p: 5.7e-07

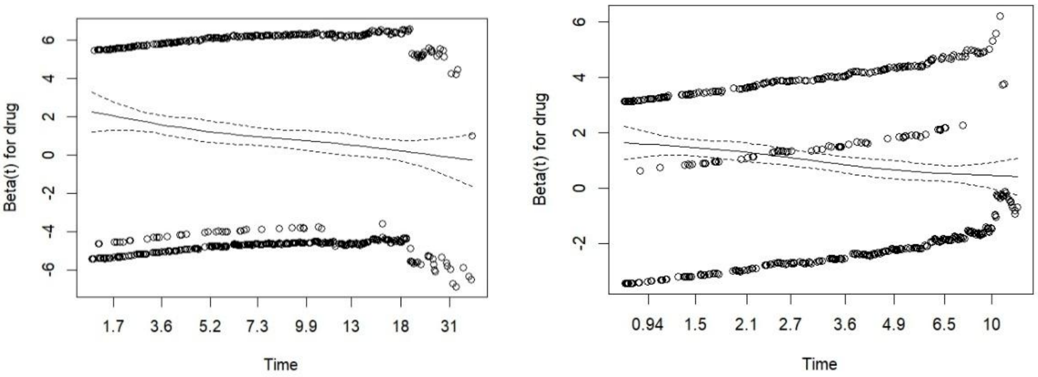

Fig. S4. Proportional hazards assumption, Schoenfeld residuals testing for OS and PFS among lenvatinib, sorafenib and regorafenib

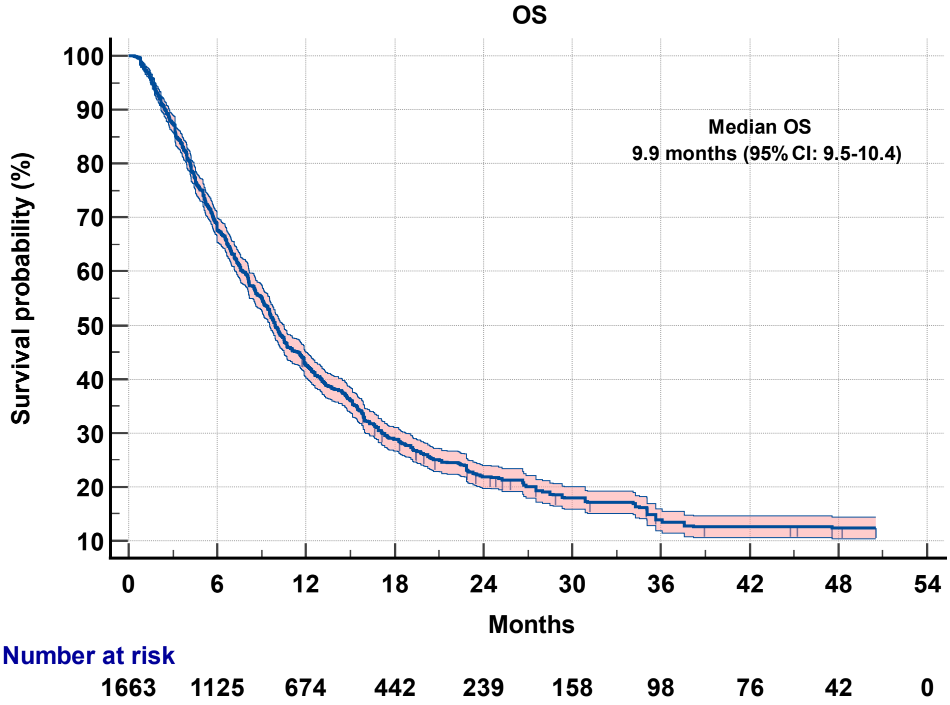

Fig. S5. Pooled overall survival (OS) with second-line tyrosine kinase inhibitors (TKIs) (lenvatinib, sorafenib, regorafenib, cabozantinib) in advanced hepatocellular carcinoma (HCC)

a)

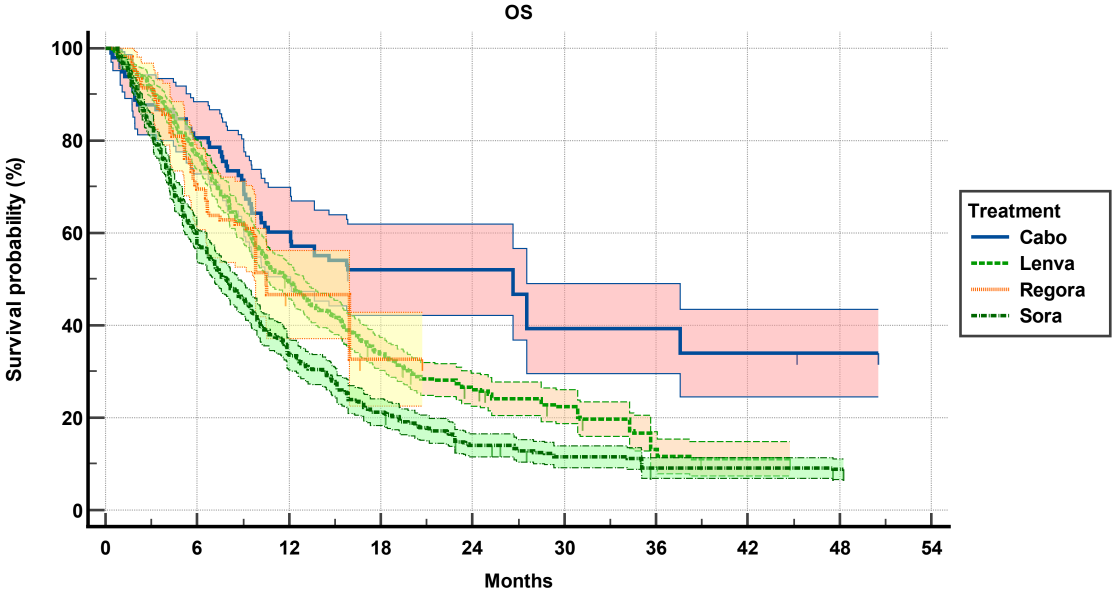

Number at risk

Group	0	6	12	18	24	30	36	42	48	54
Group: Cabo	98	79	59	49	49	37	37	32	20	0
Group: Lenva	691	532	326	221	95	58	26	9	0	0
Group: Regora	105	73	30	9	0	0	0	0	0	0
Group: Sora	769	441	259	163	95	63	35	35	22	0

Treatment	Median OS (months)	95% CI	Comparison	HR	95% CI
**Cabo**	26.6	10.6-27.5	**Lenva vs Sora**	**0.68**	**0.61-0.77**
**Lenva**	11.9	10.5-12.9	**Regora vs Sora**	**0.69**	**0.54-0.89**
**Regora**	10.4	9.6-15.9	**Cabo vs. Sora**	**0.42**	**0.34-0.51**
**Sora**	7.9	7.1-8.6	**Lenva vs. Regora**	0.98	0.77-1.25
**P<0.001**			**Lenva vs. Cabo**	**1.62**	**1.33-1.98**
			**Regora vs Cabo**	**1.65**	**1.23-2.21**

b)

Treatment	RMST 12-month OS (month difference, 95% CI)	*p*	RMST 20-month OS (month difference, 95% CI)	*p*
**Lenva vs Sora**	**1.49 (1.10-1.88)**	**<0.001**	**2.58 (1.90-3.26)**	**<0.001**
**Regora vs Sora**	**1.16 (0.41-1.96)**	**0.002**	**2.30 (0.90-3.70)**	**0.001**
**Cabo vs. Sora**	**1.88 (1.09-2.66)**	**<0.001**	**4.15 (2.67-5.62)**	**<0.001**
**Lenva vs. Regora**	0.32 (-0.42-1.07)	0.393	0.27 (-1.12-1.68)	0.697
**Lenva vs. Cabo**	-0.38 (-1.16- 0.39)	0.332	**-1.57 (-3.05- -0.08)**	**0.037**
**Regora vs Cabo**	-0.71 (-1.73-0.30)	0.169	-1.85 (-3.77- 0.07)	0.059

Fig. S6. a) Comparison of OS across TKIs in the second-line treatment of advanced HCC b) RMST analyses for 12-month and 20-month OS. OS: Overall survival, Cabo: cabozantinib, Lenva: Lenvatinib, Regora: Regorafenib, Sora: Sorafenib, CI: confidence interval, HR: Hazard ratio, RMST: Restricted mean survival time, TKIs: tyrosine kinase inhibitors

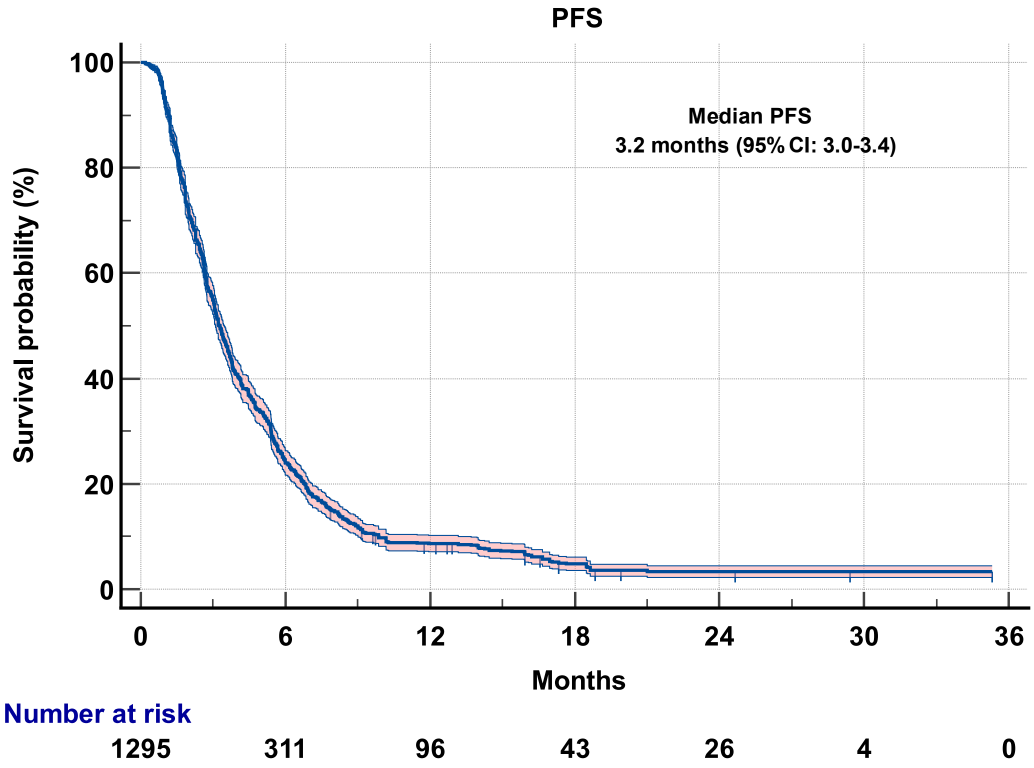

Fig. S7. Pooled progression-free survival (PFS) with second-line tyrosine kinase inhibitors (TKIs) in advanced hepatocellular carcinoma (HCC)

a)

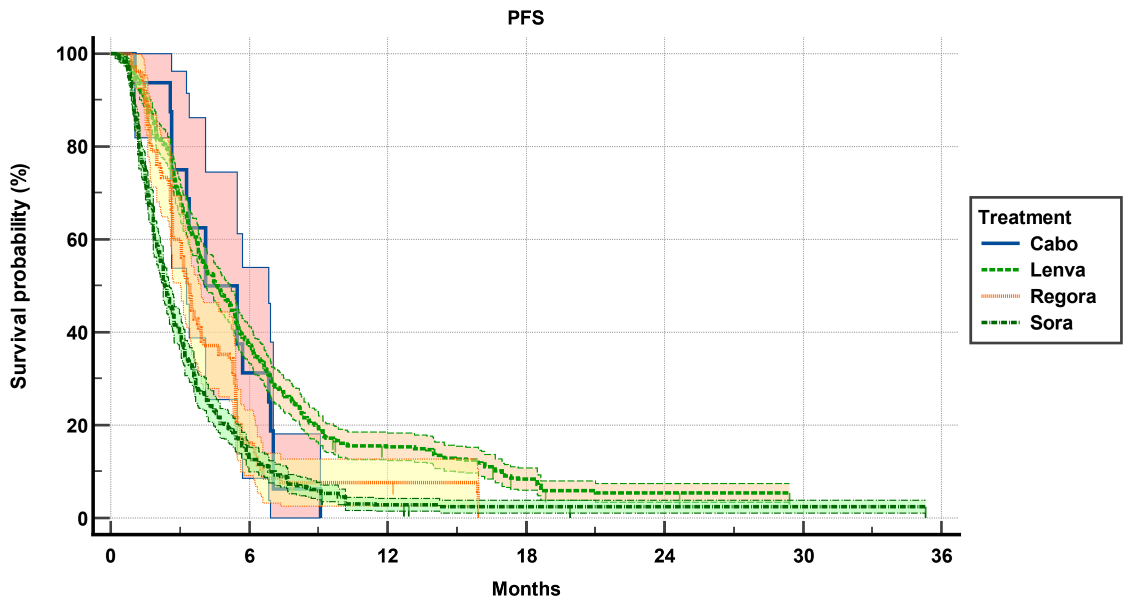

Number at risk

	0	6	12	18	24	30	36
Group: Cabo	16	5	0	0	0	0	0
Group: Lenva	570	213	75	37	22	0	0
Group: Regora	105	17	6	0	0	0	0
Group: Sora	604	76	15	6	4	4	0

Treatment	Median PFS (months)	95% CI	Comparison	HR	95% CI
**Cabo**	4.1	2.6-6.8	**Lenva vs Sora**	**0.51**	**0.45-0.58**
**Lenva**	4.5	4.0-5.1	**Regora vs Sora**	**0.76**	**0.61-0.96**
**Regora**	3.4	2.7-3.9	Cabo vs. Sora	0.63	0.39-1.02
**Sora**	2.3	2.2-2.5	**Lenva vs. Regora**	**0.66**	**0.53-0.83**
**P<0.001**			Lenva vs. Cabo	0.80	0.50-1.30
			Regora vs Cabo	1.21	0.72-2.01

b)

Treatment	RMST 6-month PFS (month difference, 95% CI)	*p*	RMST 9-month PFS (month difference, 95% CI)	*p*
**Lenva vs Sora**	**1.28 (1.08-1.49)**	**<0.001**	**1.84 (1.54-2.13)**	**<0.001**
**Regora vs Sora**	**0.73 (0.38-1.09)**	**<0.001**	**0.74 (0.28-1.19)**	**0.001**
**Cabo vs. Sora**	**1.48 (0.69-2.27)**	**<0.001**	**1.64 (0.59-2.69)**	**0.002**
**Lenva vs. Regora**	**0.54 (0.19-0.90)**	**0.002**	**1.10 (0.62-1.57)**	**<0.001**
**Lenva vs. Cabo**	-0.19 (-0.98- 0.58)	0.622	0.19 (-0.86-1.25)	0.718
**Regora vs Cabo**	-0.74 (-1.58-0.09)	0.080	-0.90 (-2.01- 0.20)	0.110

Fig. S8. a) Comparison of PFS across TKIs in the second-line treatment of advanced HCC b) RMST analyses for 6-month and 9-month PFS. PFS: Progression-free survival, Cabo: cabozantinib, Lenva: Lenvatinib, Regora: Regorafenib, Sora: Sorafenib, CI: confidence interval, HR: Hazard ratio, RMST: Restricted mean survival time, TKIs: tyrosine kinase inhibitors

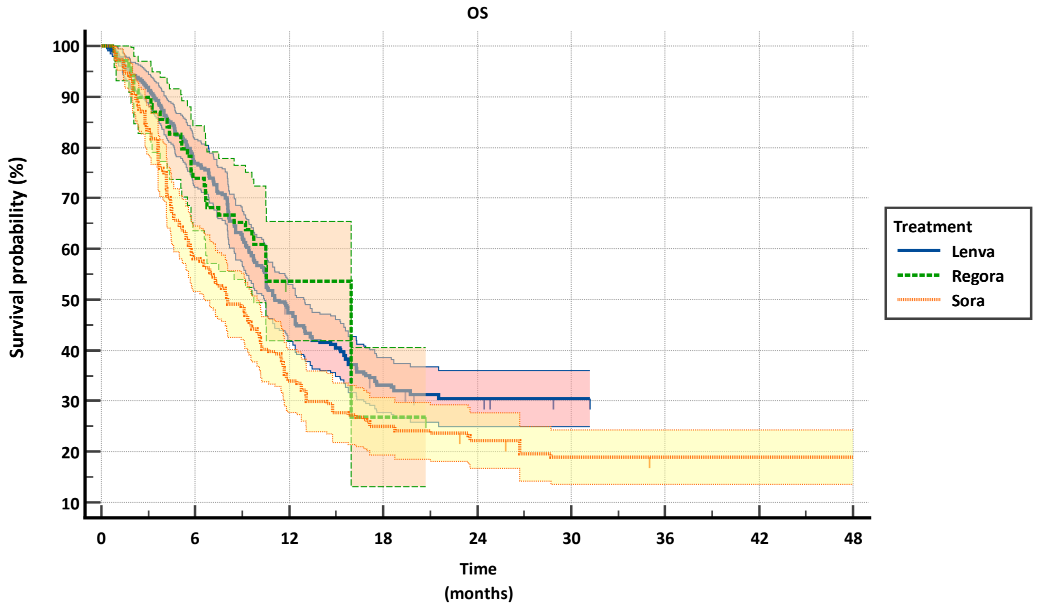

Number at risk

**Group: Lenva**								
307	236	130	89	38	12	0	0	0
**Group: Regora**								
69	51	18	9	0	0	0	0	0
**Group: Sora**								
224	130	76	56	45	29	22	22	22

Treatment	Median OS (months)	95% CI	Comparison	HR	95% CI
**Lenva**	11.0	10.3-12.9	**Lenva vs Sora**	**0.70**	**0.57-0.86**
**Regora**	15.9	NC	**Regora vs Sora**	**0.68**	**0.49-0.95**
**Sora**	8	6.5-9.9	**Lenva vs. Regora**	1.01	0.74-1.39

Treatment	RMST 12-month OS (month difference, 95% CI)	*p*	RMST 20-month OS (month difference, 95% CI)	*p*
**Lenva vs Sora**	**1.42 (0.77-2.07)**	**<0.001**	**2.24 (1.08-3.39)**	**<0.001**
**Regora vs Sora**	**1.39 (0.37-2.40)**	**0.007**	**2.38 (0.56-4.20)**	**0.010**
**Lenva vs. Regora**	0.02 (-0.92-0.98)	0.951	-0.14 (-1.89-1.60)	0.870

Fig. S9. OS sensitivity analyses by excluding multicountry studies. a) Comparison of OS across TKIs in the second-line treatment of advanced HCC b) RMST analyses for 12-month and 20-month OS. OS: Overall survival, Cabo: cabozantinib, Lenva: Lenvatinib, Regora: Regorafenib, Sora: Sorafenib, CI: confidence interval, HR: Hazard ratio, RMST: Restricted mean survival time, TKIs: tyrosine kinase inhibitors

a) Hand-foot syndrome (any grade)

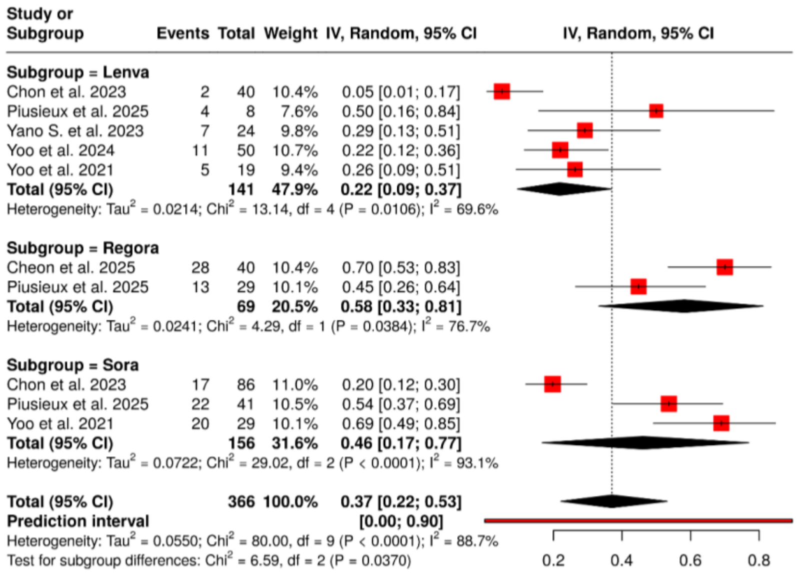

b) Fatigue (any grade)

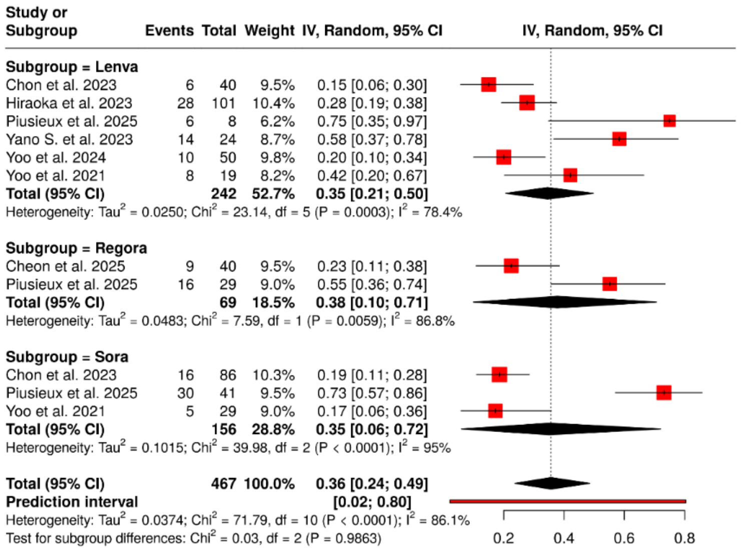

c) Hypertension (Any grade)

Study or Subgroup	Events	Total	Weight	IV, Random, 95% CI
**Subgroup = Lenva**				
Chon et al. 2023	17	40	9.5%	0.42 [0.27; 0.59]
Hiraoka et al. 2023	19	101	10.7%	0.19 [0.12; 0.28]
Piusieux et al. 2025	2	8	5.7%	0.25 [0.03; 0.65]
Yano S. et al. 2023	15	24	8.6%	0.62 [0.41; 0.81]
Yoo et al. 2024	11	50	9.9%	0.22 [0.12; 0.36]
Yoo et al. 2021	8	19	8.0%	0.42 [0.20; 0.67]
**Total (95% CI)**		**242**	**52.4%**	**0.34 [0.20; 0.49]**
Heterogeneity: $Tau^2 = 0.0243$; $Chi^2 = 22.65$, df = 5 (P = 0.0004); $I^2 = 77.9\%$				
**Subgroup = Regora**				
Cheon et al. 2025	6	40	9.5%	0.15 [0.06; 0.30]
Piusieux et al. 2025	1	29	9.0%	0.03 [0.00; 0.18]
**Total (95% CI)**		**69**	**18.5%**	**0.09 [0.01; 0.23]**
Heterogeneity: $Tau^2 = 0.0101$; $Chi^2 = 2.38$, df = 1 (P = 0.1226); $I^2 = 58.1\%$				
**Subgroup = Sora**				
Chon et al. 2023	5	86	10.5%	0.06 [0.02; 0.13]
Piusieux et al. 2025	7	41	9.6%	0.17 [0.07; 0.32]
Yoo et al. 2021	5	29	9.0%	0.17 [0.06; 0.36]
**Total (95% CI)**		**156**	**29.1%**	**0.12 [0.04; 0.22]**
Heterogeneity: $Tau^2 = 0.0089$; $Chi^2 = 5.32$, df = 2 (P = 0.0699); $I^2 = 62.4\%$				
**Total (95% CI)**		**467**	**100.0%**	**0.22 [0.13; 0.32]**
**Prediction interval**				**[0.00; 0.61]**
Heterogeneity: $Tau^2 = 0.0286$; $Chi^2 = 57.18$, df = 10 (P < 0.0001); $I^2 = 82.5\%$				
Test for subgroup differences: $Chi^2 = 9.30$, df = 2 (P = 0.0096)				

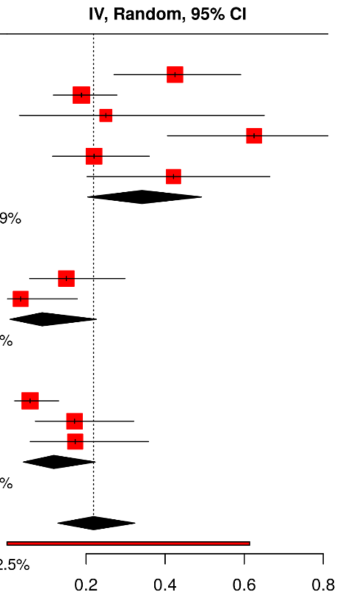

d) Diarrhea (Any grade)

Study or Subgroup	Events	Total	Weight	IV, Random, 95% CI
**Subgroup = Lenva**				
Chon et al. 2023	16	40	11.3%	0.40 [0.25; 0.57]
Piusieux et al. 2025	1	8	3.3%	0.12 [0.00; 0.53]
Yano S. et al. 2023	10	24	7.9%	0.42 [0.22; 0.63]
Yoo et al. 2024	16	50	12.9%	0.32 [0.20; 0.47]
Yoo et al. 2021	5	19	6.7%	0.26 [0.09; 0.51]
**Total (95% CI)**		**141**	**42.1%**	**0.34 [0.26; 0.42]**
Heterogeneity: $Tau^2 = 0$; $Chi^2 = 3.25$, df = 4 (P = 0.5174); $I^2 = 0\%$				
**Subgroup = Regora**				
Cheon et al. 2025	6	40	11.3%	0.15 [0.06; 0.30]
Piusieux et al. 2025	4	29	9.1%	0.14 [0.04; 0.32]
**Total (95% CI)**		**69**	**20.3%**	**0.14 [0.07; 0.24]**
Heterogeneity: $Tau^2 = 0$; $Chi^2 = 0.01$, df = 1 (P = 0.9204); $I^2 = 0\%$				
**Subgroup = Sora**				
Chon et al. 2023	20	86	17.1%	0.23 [0.15; 0.34]
Piusieux et al. 2025	10	41	11.4%	0.24 [0.12; 0.40]
Yoo et al. 2021	6	29	9.1%	0.21 [0.08; 0.40]
**Total (95% CI)**		**156**	**37.6%**	**0.23 [0.17; 0.30]**
Heterogeneity: $Tau^2 = 0$; $Chi^2 = 0.11$, df = 2 (P = 0.9474); $I^2 = 0\%$				
**Total (95% CI)**		**366**	**100.0%**	**0.25 [0.19; 0.31]**
**Prediction interval**				**[0.12; 0.40]**
Heterogeneity: $Tau^2 = 0.0035$; $Chi^2 = 13.49$, df = 9 (P = 0.1417); $I^2 = 33.3\%$				
Test for subgroup differences: $Chi^2 = 10.12$, df = 2 (P = 0.0063)				

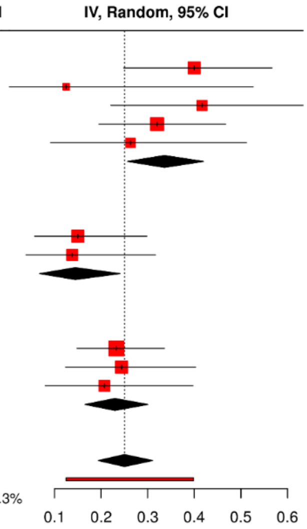

e) Proteinuria with lenvatinib (Any grade)

Study	Events	Total	Weight	IV, Random, 95% CI
Chon et al. 2023	23	40	24.5%	0.57 [0.41; 0.73]
Hiraoka et al. 2023	30	101	28.6%	0.30 [0.21; 0.40]
Yano S. et al. 2023	7	24	21.1%	0.29 [0.13; 0.51]
Yoo et al. 2024	10	50	25.7%	0.20 [0.10; 0.34]
**Total (95% CI)**		**215**	**100.0%**	**0.33 [0.19; 0.49]**
**Prediction interval**				**[0.00; 0.82]**

Heterogeneity: Tau^2 = 0.0194; Chi^2 = 14.42, df = 3 (P = 0.0024); I^2 = 79.2%

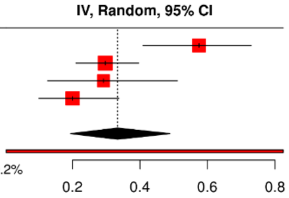

Fig. S10. Pooled and comparative analyses of adverse events.

Supplementary tables

Table S1. Risk of bias assessment of treatment arms (ROB-ME tool and modified Cowley's criteria scoring)

Study	Treatment	Number of participants analysed	Result available for OS	Result available for PFS	Result available for ECOG-0	Result available for CP-A	Result available for BCLC-C	Result available for ALBI Grade 1	Result available for MVI	Result available for extrahepatic spread	Result available for toxicities	Result available for response	Modified Cowley's criteria score	Risk group modified Cowley's criteria
**Chen et al, 2022**	Lenva	9	✓	✓	~	✓	~	✓	✓	✓	~	✓	21	Moderate
**Chon et al, 2023**	Lenva	40	✓	✓	✓	✓	✓	~	✓	✓	✓	✓	24	Low
**Decreacker et al, 2025**	Lenva	35	✓	~	~	~	~	~	~	~	~	~	22	Moderate
**Hiraoka,2023**	Lenva	101	✓	✓	✓	✓	✓	✓	✓	✓	✓	✓	22	Moderate
**Lee et al, 2025**	Lenva	154	✓	✓	✓	✓	✓	~	✓	✓	~	✓	22	Moderate
**Lombardi et al, 2025**	Lenva	125	✓	✓	✓	~	✓	✓	~	✓	~	~	21	Moderate
**Muto et al, 2023**	Lenva	20	✓	✓	✓	✓	✓	✓	~	✓	~	✓	21	Moderate
**Persano et al, 2024**	Lenva	86	✓	~	~	✓	✓	✓	~	~	~	✓	20	Moderate
**Piusieux et al, 2025**	Lenva	8	✓	✓	✓	✓	✓	✓	✓	✓	✓	~	23	Moderate
**Qin et al, 2022**	Lenva	20	✓	✓	~	✓	✓	~	~	✓	~	✓	19	High
**Yano S. et al, 2023**	Lenva	24	✓	✓	✓	✓	✓	~	✓	✓	✓	✓	22	Moderate
**Yoo et al, 2024**	Lenva	50	✓	✓	✓	✓	✓	~	✓	~	✓	✓	25	Low
**Yoo et al, 2021**	Lenva	19	✓	✓	✓	✓	✓	~	✓	~	✓	✓	25	Low
**Chen et al, 2022**	Sora	19	✓	✓	~	✓	~	~	✓	✓	~	✓	22	Moderate
**Chon et al, 2023**	Sora	86	✓	✓	✓	✓	✓	~	✓	✓	✓	✓	24	Low
**Decreacker et al, 2025**	Sora	78	✓	~	~	~	~	~	~	~	~	~	22	Moderate
**Lee et al, 2025**	Sora	324	✓	✓	✓	✓	✓	~	✓	✓	~	✓	22	Moderate
**Lombardi et al, 2025**	Sora	105	✓	✓	✓	~	✓	✓	~	✓	~	~	22	Moderate
**Möhring et al, 2025**	Sora	36	✓	~	~	~	~	~	~	~	~	~	20	Moderate
**Persano et al, 2024**	Sora	51	✓	~	~	✓	✓	✓	~	~	~	✓	20	Moderate
**Piusieux et al, 2025**	Sora	41	✓	✓	✓	✓	✓	✓	✓	✓	✓	~	23	Moderate
**Yoo et al, 2021**	Sora	29	✓	✓	✓	✓	✓	~	✓	~	✓	✓	25	Low
**Cheon et al., 2025**	Regora	40	✓	✓	✓	✓	✓	✓	✓	✓	✓	✓	25	Low
**Lee et al, 2025**	Regora	36	✓	✓	✓	✓	✓	~	✓	✓	~	✓	22	Moderate
**Piusieux et al, 2025**	Regora	29	✓	✓	✓	✓	✓	✓	✓	✓	✓	~	23	Moderate
**Ahn et al., 2025 (1L only IO)**	Cabo	28	✓	?	~	~	~	~	~	~	~	~	18	High
**Ahn et al., 2025 (1KL IO-antiVEGF)**	Cabo	54	✓	?	~	~	~	~	~	~	~	~	18	High
**Lee et al, 2025**	Cabo	12	✓	✓	✓	✓	✓	~	✓	✓	~	✓	22	Moderate
**Piusieux et al, 2025**	Cabo	4	✓	✓	✓	✓	✓	✓	✓	✓	✓	~	23	Moderate
Risk of bias judgment	-	-	***Low***	***Some concern***	***Some concern***	***Some concern***	***Some concern***	***Some concern***	***Some concern***	***Some concern***	***High***	***High***	**-**	**-**

✓: A study result is available for inclusion in the meta-analysis, ~No study result is available for inclusion in the meta-analysis, for a reason unrelated to the P value, magnitude or direction of the result, ?: Unclear whether an eligible study result was generated, X: No study result is available for inclusion in the meta-analysis, likely because of the P value, magnitude or direction of the result generated

OS: Overall survival, PFS: Progression-free survival, Lenva: Lenvatinib, Sora: Sorafenib, Regora: Regorafenib, Cabo: Cabozantinib, ECOG-PS: European Cooperation Oncology Group performance score, CP: Child-Pugh, c) BCLC: Barcelona Clinic Liver Cancer, MVI: Macrovascular invasion

Table S2. Adverse events (any grade, observed in >5% of the patients) with the second-line tyrosine kinase inhibitors (TKIs)

Study	Year	Treatment	Sample size	Hand-foot syndrome	Anorexia	Fatigue	Hyperbilirubinemia	AST increased	Nausea	Thrombocytopenia	Hypertension	Diarrhea	Proteinuria	Pruritus	ALT increased	Hypothyroidism	Anemia	Oral mucositis	Neutropenia	GI bleeding	Constipation
**Piusieux et al.**	2025	Cabo	4	1	NA	2	NA	NA	0	NA	1	1	NA	NA	NA	NA	NA	NA	NA	NA	NA
**Chon et al.**	2023	Lenva	40	2	16	6	16	20	10	20	17	16	23	4	15	14	10	6	4	3	3
**Hiraoka et al.**	2023	Lenva	101	NA	37	28	NA	NA	NA	NA	19	NA	30	NA	NA	NA	NA	NA	NA	NA	NA
**Piusieux et al.**	2025	Lenva	8	4	NA	6	NA	NA	2	NA	2	1	NA	NA	NA	NA	NA	NA	NA	NA	NA
**Yano S. et al.**	2023	Lenva	24	7	11	14	NA	NA		2	15	10	7	NA	NA	5	NA	NA	NA	NA	NA
**Yoo et al.**	2024	Lenva	50	11	15	10	7	5	10	5	11	16	10	6	NA	15	NA	NA	NA	NA	NA
**Yoo et al.**	2021	Lenva	19	5	4	8	2	NA		3	8	5	NA	NA	NA	NA	2	NA	NA	NA	NA
**Cheon et al.**	2025	Regora	40	28	10	9	9	8	7	7	6	6	6	5	5	3	NA	NA	NA	NA	NA
**Piusieux et al.**	2025	Regora	29	13	NA	16	NA	NA	1	NA	1	4	NA	NA	NA	NA	NA	NA	NA	NA	NA
**Chon et al.**	2023	Sora	86	17	17	16	47	45	11	19	5	20	3	6	22	1	8	6	5	3	1
**Piusieux et al.**	2025	Sora	41	22	NA	30	NA	NA	3	NA	7	10	NA	NA	NA	NA	NA	NA	NA	NA	NA
**Yoo et al.**	2021	Sora	29	20	2	5	4	NA		6	5	6	NA	NA	NA	NA	1	NA	NA	NA	NA

NA: Not available, Cabo: Cabozantinib, Lenva: Lenvatinib, Regora: Regorafenib, Sora: Sorafenib
